# Hecatomb: an integrated software platform for viral metagenomics

**DOI:** 10.1093/gigascience/giae020

**Published:** 2024-06-04

**Authors:** Michael J Roach, Sarah J Beecroft, Kathie A Mihindukulasuriya, Leran Wang, Anne Paredes, Luis Alberto Chica Cárdenas, Kara Henry-Cocks, Lais Farias Oliveira Lima, Elizabeth A Dinsdale, Robert A Edwards, Scott A Handley

**Affiliations:** Flinders Accelerator for Microbiome Exploration, Flinders University, Adelaide, SA, Australia; Adelaide Centre for Epigenetics, University of Adelaide, Adelaide, SA, 5005, Australia; South Australian Immunogenomics Cancer Institute, University of Adelaide, Adelaide, SA, 5005, Australia; Harry Perkins Institute of Medical Research, Perth, WA, 6009, Australia; Department of Pathology & Immunology, Washington University School of Medicine, St. Louis, MO, 63110, USA; The Edison Family Center for Genome Sciences & Systems Biology, Washington University School of Medicine, St. Louis, MO, 63110, USA; Department of Pathology & Immunology, Washington University School of Medicine, St. Louis, MO, 63110, USA; The Edison Family Center for Genome Sciences & Systems Biology, Washington University School of Medicine, St. Louis, MO, 63110, USA; Department of Pathology & Immunology, Washington University School of Medicine, St. Louis, MO, 63110, USA; Department of Pathology & Immunology, Washington University School of Medicine, St. Louis, MO, 63110, USA; The Edison Family Center for Genome Sciences & Systems Biology, Washington University School of Medicine, St. Louis, MO, 63110, USA; Flinders Accelerator for Microbiome Exploration, Flinders University, Adelaide, SA, Australia; Biology Department, San Diego State University, San Diego, CA, 92182, USA; Flinders Accelerator for Microbiome Exploration, Flinders University, Adelaide, SA, Australia; Flinders Accelerator for Microbiome Exploration, Flinders University, Adelaide, SA, Australia; Department of Pathology & Immunology, Washington University School of Medicine, St. Louis, MO, 63110, USA; The Edison Family Center for Genome Sciences & Systems Biology, Washington University School of Medicine, St. Louis, MO, 63110, USA

**Keywords:** virome, virus discovery, bioinformatic workflow, viral metagenomics

## Abstract

**Background:**

Modern sequencing technologies offer extraordinary opportunities for virus discovery and virome analysis. Annotation of viral sequences from metagenomic data requires a complex series of steps to ensure accurate annotation of individual reads and assembled contigs. In addition, varying study designs will require project-specific statistical analyses.

**Findings:**

Here we introduce Hecatomb, a bioinformatic platform coordinating commonly used tasks required for virome analysis. Hecatomb means “a great sacrifice.” In this setting, Hecatomb is “sacrificing” false-positive viral annotations using extensive quality control and tiered-database searches. Hecatomb processes metagenomic data obtained from both short- and long-read sequencing technologies, providing annotations to individual sequences and assembled contigs. Results are provided in commonly used data formats useful for downstream analysis. Here we demonstrate the functionality of Hecatomb through the reanalysis of a primate enteric and a novel coral reef virome.

**Conclusion:**

Hecatomb provides an integrated platform to manage many commonly used steps for virome characterization, including rigorous quality control, host removal, and both read- and contig-based analysis. Each step is managed using the Snakemake workflow manager with dependency management using Conda. Hecatomb outputs several tables properly formatted for immediate use within popular data analysis and visualization tools, enabling effective data interpretation for a variety of study designs. Hecatomb is hosted on GitHub (github.com/shandley/hecatomb) and is available for installation from Bioconda and PyPI.

## Background

Viruses are also the most dominant entity on the planet with current global estimates as high as 10^31^ viral particles [1], and they are omnipresent in all cellular life forms [2]. As such, they exert significant influence on their surroundings. Metagenomic sequencing offers a powerful tool for studying viral diversity in both host-associated and environmental systems [3–13]. However, there are currently many challenges associated with viral metagenomics. While viruses are the most abundant and diverse biological entity on the planet, they represent a minority of reference genomes in GenBank, largely due to difficulties associated with studying them [14]. There is a vast amount of sequence information that remains taxonomically or functionally ill-defined. These sequences are regularly referred to as “viral dark matter” and pose a significant barrier to annotating viral sequences from metagenomic data (reviewed in [15]). In addition, researchers must contend with how to annotate viral sequences from viruses with either RNA- or DNA-based genomes. Our ability to successfully annotate metagenomic data as “viral” is directly impacted by the size and diversity of the reference database and the sensitivity of our search algorithms. Larger, more diverse reference databases can improve viral sequence annotation but are less conducive to high-sensitivity search algorithms required to identify distant sequence similarity. This dichotomy can force researchers to choose between optimal databases and search algorithms.

Another challenge to the interpretation of reference-based sequence annotation is that viral metagenomes are often plagued with false-positive classifications [16–18]. Viruses share regions of sequence similarity with all other domains of life, including “stolen” genes incorporated from their hosts’ genomes and repetitive or low-complexity insertion elements or transposons. These sequences are present in many reference databases and can result in false classifications due to shared sequence similarity across taxonomies. The presence of false-positive classifications may influence data interpretation. For instance, the misclassification of viral sequences in clinical samples could lead to incorrect hypotheses about virus–disease associations or patient diagnosis. Similarly, an increased false-positive rate in any environment could lead to overestimates of species diversity. Highly curated databases may alleviate false positives, but they require tremendous resources and time. Likewise, they risk missing newly discovered viruses that have yet to make their way through the curation process. Thus, it is important for bioinformatic tools to provide a system to classify the quality of similarity-based annotations in light of imperfect databases.

Numerous bioinformatics tools exist for identifying viral sequences from metagenomic data [19–44]. However, many of these are lacking in features or fail to offer researchers an end-to-end solution to manage the myriad tasks required of virome analysis (e.g., quality control, host removal, assembly). For example, few tools are designed for read-based annotation of viral sequences and none are currently maintained [45, 46]. Individual read annotations are valuable as detecting a small number of viral reads could signify the presence of a virus. This is true for viral sequences both closely related to and divergent from reference viral sequences. Detection of divergent viral sequences requires sensitive alignment-based tools such as BLAST, DIAMOND, or MMSeqs2 [47–49]. Alternative approaches, such as those that use *k*-mer distances, have also been implemented [50–53]. The *k*-mer–based algorithms are fast but limited in their ability to annotate viral sequences divergent from those in reference databases. Therefore, sensitive alignment-based approaches are preferred for detecting divergent or novel viruses.

Alignment-based approaches are computationally more expensive than *k*-mer–based approaches but can be effectively implemented using a tiered database query approach [45,54]. In the tiered approach, initial queries are made against small virus-only sequence databases. Subsequent secondary cross-checking against reference databases representing sequences from all domains of life is required to remove false-positive viral annotations. In addition, queries against both amino acid and nucleotide databases may be of interest. Reference sequences from viral taxa may only be represented in one or the other database types, and amino acid databases will not include noncoding viral sequences. Tiered alignment-based queries across multiple databases require a number of steps and produce a series of disconnected outputs. A robust system to monitor and manage these steps and coordinate the outputs into a tractable framework would permit researchers to focus on making biological insights instead of on job and file management.

While read-based annotation can provide sensitive and specific detection of viral sequences within a metagenome, metagenome assembled contigs can provide additional layers of information such as gene content, gene order, and metabolic pathway prediction. Many of the tools used to assemble viral contigs are the same ones used for assembling bacterial contigs [55–58]. More recently, metavirome-specific assembly tools have begun to emerge with promising results [59,60]. Virome metagenomics is evolving beyond binning with algorithms specifically designed to resolve complete genomes from metavirome assemblies [61]. Similar to the requirements of read-based annotations, assembly requires multiple steps to ensure high-quality data for downstream analysis.

Once contigs are obtained, they can be further analyzed using one of many established virome analysis tools [23,28,31,43]. Each of these tools provides distinct information about the viral content of a metagenome. For example, VirSorter2, Cenote-Taker 2, and geNomad use customized classifiers and curated hidden Markov models (HMMs) to estimate the “viralness” of metagenome assembled contigs. This is useful for separating viral from nonviral contigs, but additional steps are required to assign taxonomic lineages. vContact2 can assign genus-level taxonomy using gene-sharing networks to prokaryotic but not eukaryotic viruses. VIBRANT uses deep learning neural networks to classify prokaryotic viral contigs. Numerous additional tools are also available for researchers to mine taxonomic and functional information from a metagenome [62]. This complex array of tools for virome interrogation provides a number of opportunities for virome researchers. However, each of them is dependent on the generation of high-quality input assembly data from a wide range of experimental systems, and they vary greatly in terms of useability and support. A common workflow that takes inputs from both short and long reads and emphasizes rigorous quality control to remove nonbiological contamination and host from a variety of library types and study designs would ensure researchers provide the highest quality of data to each of these tools regardless of their experimental system.

While several options are available for virome analysis using read- or contig-based approaches, integrating these results with study data is critical for making biological insights. Project-specific statistical analyses are often required. For example, statistical models to test if a pathogenic virus is associated with a disease using sparse read-based results will differ wildly from a study analyzing bacteriophage ecology. Fortunately, the vast majority of these disparate statistical approaches are available as R software packages [63]. In addition, principles guided by the popular tidyverse and ggplot2 packages are familiar to many researchers [64,65]. These principles have been successfully applied to the analysis of bacterial microbiome data in software suites such as PhyloSeq and microViz but have yet to be applied to the analysis of virome data [66,67].

Here we present Hecatomb, a bioinformatics platform designed to address the above issues. Hecatomb supports the analysis of both long- and short-read technologies and various library types. The pipeline performs rigorous quality control followed by tiered alignment-based taxonomic assignment using MMseqs2 [45]. Hecatomb also performs metagenomic assembly and annotation. Each step is managed using the Sankemake workflow manager with dependency management using Conda [68,69]. Hecatomb outputs several tables properly formatted for immediate use within popular data analysis and visualization tools enabling effective data interpretation for a variety of study designs.

## Implementation

**Figure 1:**
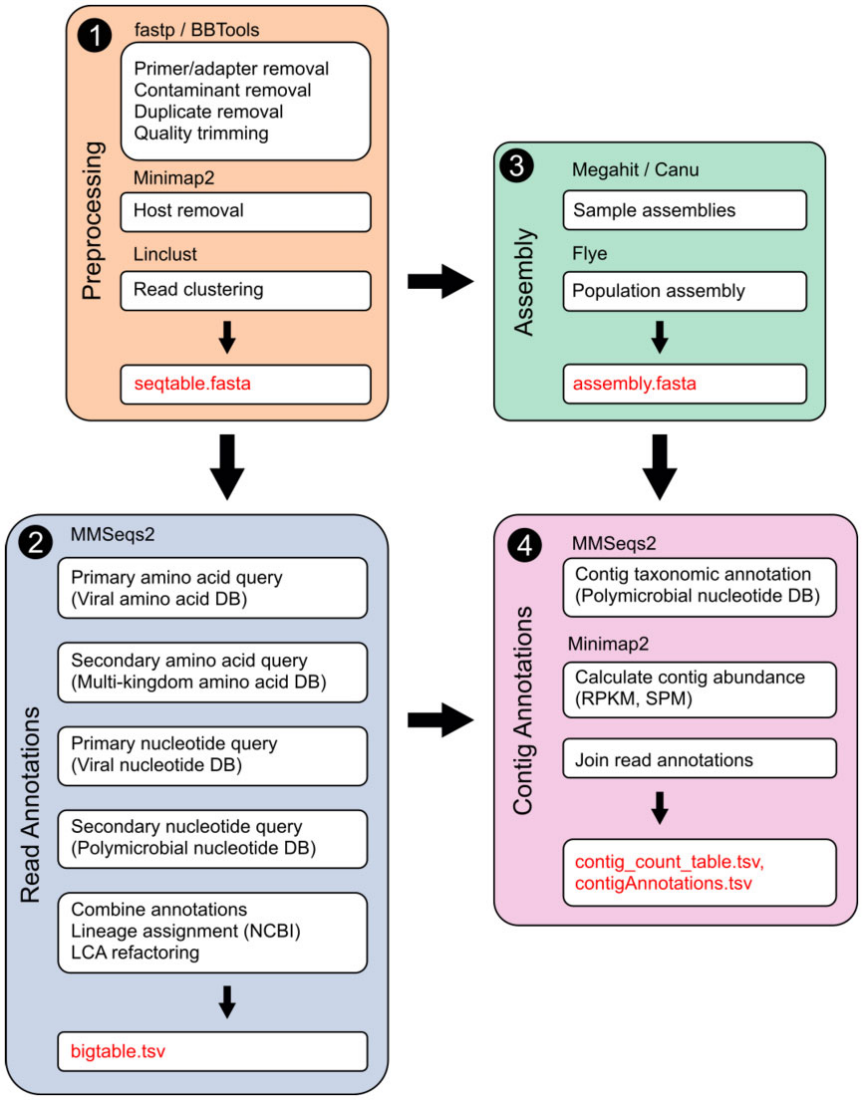
Hecatomb pipeline and implementation. The Hecatomb pipeline is divided into 4 modules. (1) Reads for each sample undergo preprocessing and clustering (*orange*); (2) clustered reads undergo annotation using viral and multikingdom protein databases, and clustered reads not annotated by the protein search are annotated using viral and multikingdom nucleotide databases (*blue*); (3) quality trimmed reads for each sample undergo assembly, and assemblies for each sample are coalesced into a single assembly (*green*); and (4) read-based annotations are combined with the assembly to provide contig annotations (*pink*). The assembly stages—*green* and *pink*–can optionally be skipped.

Hecatomb (RRID: SCR_025002) serves as an end-to-end pipeline by processing raw sequencing reads (single or paired end, long or short reads from Illumina, MGI, PacBio, or Oxford Nanopore platforms) through 4 key modules (Fig. 1). In this way, it was designed to address many of the useability and functionality issues that are present in other software that we summarize in Supplementary Fig. S1.

### Module 1: Sequence quality control and host removal

Module 1 (preprocessing) removes nonbiological contaminants (i.e., primers, adapters) as well as common laboratory contaminants cataloged in NCBI’s UniVec database [70]. The user can select to use fastp for contaminant removal from standard library preps or BBTools for more complicated library preparation strategies such as the round A/B library, which converts RNA into cDNA using a tagged-randomized primer and second-strand DNA synthesis; the resulting dsDNA is subjected to 10–40 rounds of PCR amplification. This process generates DNA/cDNA libraries for single- and double-stranded RNA and DNA viruses [71–73]. Low-quality sequences are also trimmed or removed prior to host removal.

Module 1 has an option to remove host sequences (e.g., mouse, human) using Minimap2 [74]. This is optional as it may not be applicable to all samples (e.g., water, air, soil). Hecatomb comes packaged with several commonly used host-reference genomes, which have been masked of potential viral sequence. This masking is designed to minimize the inadvertent removal of viral sequences that may have similarities to the host sequence. Masked reference genomes were generated as follows: (i) all viral genomes from the National Center for Biotechnology Information (NCBI) viral assembly database [75] were downloaded and computationally “shredded” into short fragments with an average length of 85 bases sharing a 30-base overlap using shred.sh from the BBTools suite [73]. Shredded viral sequences were then mapped (minimum identity of 90% and at most 2 insertions or deletions) and masked from host-reference genomes using BBmap requiring a [73]. Precomputed masked reference genomes for the following host genomes are available in Hecatomb: human, mouse, rat, camel, *Caenorhabditis elegans*, dog, cow, macaque, mosquito, pig, rat, and tick are available within Hecatomb. A command is provided to generate new masked genomes for additional hosts not included with Hecatomb.

Sequences free of contamination and host are clustered using the nucleotide version of Linclust packaged with MMSeqs2 [49,76]. Clustering reduces the number of sequences requiring taxonomic classification to a single, representative sequence, thus greatly reducing the computational requirements for read-based annotation in Module 2. Sequences are clustered requiring a minimum sequence identity of 97% and 80% alignment coverage of target sequence to the representative sequence (–min-seq-id 0.97 -c 0.8 –cov-mode 1). The size of each cluster per sample is maintained in the final output table (seqtable.fasta). This information serves as a “count table” for each sequence, and values are provided as both raw and counts normalized to library size.

### Module 2: Read-based annotation

**Figure 2:**
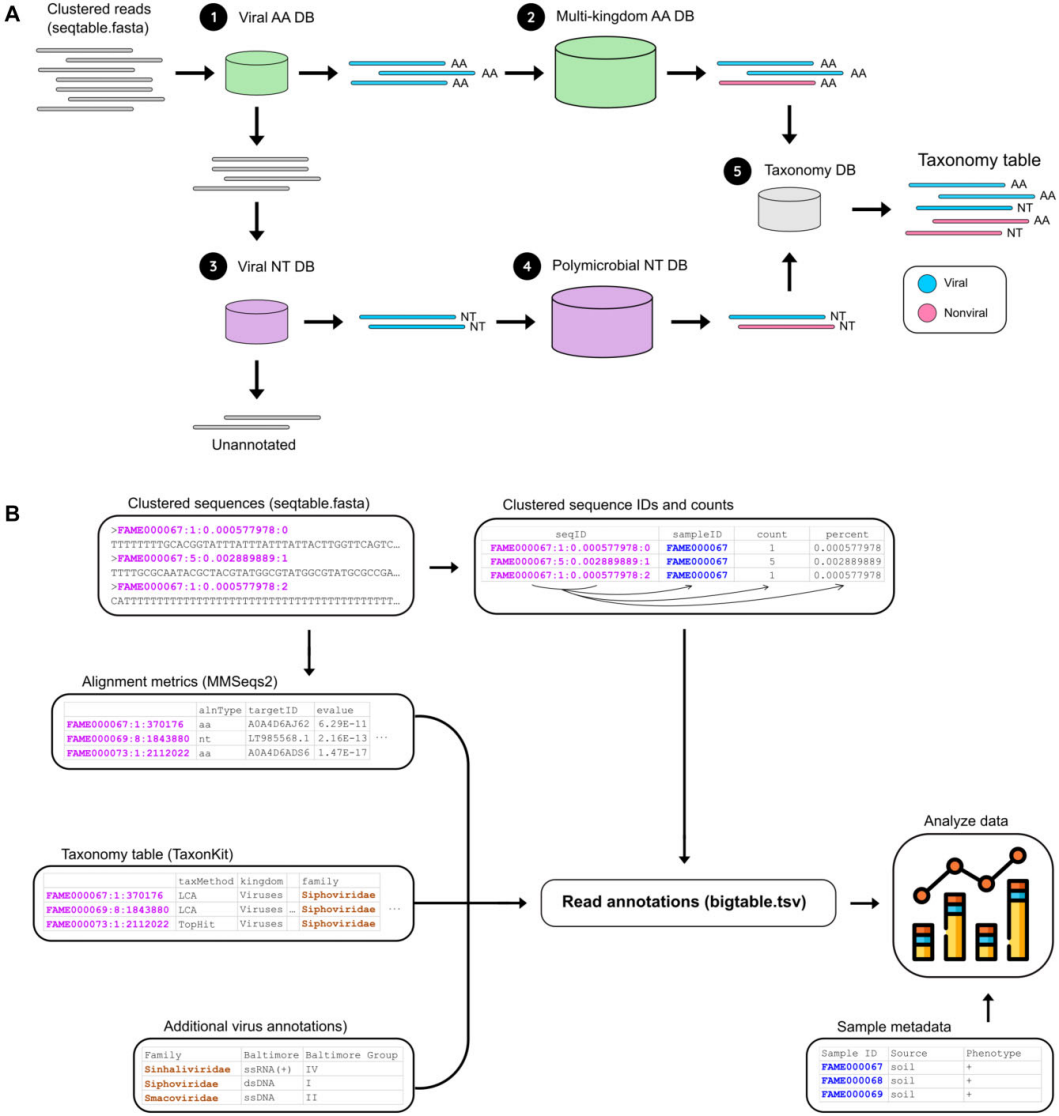
Read-based annotation. (A) Tiered annotation strategy. All alignments are completed using MMSeqs2. (1) High-quality representative sequences are queried against a viral amino acid (aa, *green*) sequence database. (2) Potentially viral sequences are subjected to a secondary, confirmatory query against a multikingdom amino acid sequence database. (3) Representative sequences that do not match a known viral amino acid sequence are subjected to an untranslated query to a viral nucleic acid sequence database (nt, *purple*), (4) followed by a secondary, confirmatory query against a polymicrobial nucleotide database (5). Sequences that have been classified as either viral (*blue*) or nonviral (*pink*) in either the translated (aa database) or untranslated (nt database) queries are combined into a final taxonomy table. (B) Read annotation data structure. (1) Read annotations are generated using the clustered sequences (seqtable.fasta). (2) The clustered sequence IDs are unpacked to yield the sample ID, the number of reads that sequence represents, and the percentage of host-removed reads that sequence represents. (3) The alignment metrics from the annotation module are joined into the read annotations using the sequence ID as the primary key. (4) Taxonomic annotations are calculated and joined into the read annotations again using the sequence ID. (5) ICTV viral classifications are joined into the read annotations by the taxonomic family annotation. (6) Sample metadata can be joined into the read annotation table using the sample ID as the primary key. (7) The read annotation table with sample metadata can be quickly and easily analyzed.

Taxonomic and functional annotation is provided to reads in seqtable.fasta using a tiered approach (Fig. 2A). All queries are carried out using MMseqs2 [49]. Queries against amino acid databases are performed using MMSeqs2 6-frame translation. Each read in seqtable.fasta is first queried against all viral (taxonomy id: 10239) amino acid sequences in UniProtKB clustered at 99% identity using Linclust (Viral AA DB) [76,77]. Sequences annotated as virus are subsequently queried against UniClust50 (Multi-kingdom AA DB) to remove false-positive annotations [78]. UniProt functional annotations are also applied when available.

Reads not identified as viral using queries against amino acid databases are queried against nucleotide databases (Fig. 2A). Similar to the translated queries, each read is first queried against a virus-only reference sequence database (Virus NT DB). This database consists of all viral sequences in GenBank (taxonomy id: 10239) clustered at 100% identity using Linclust [49,76]. Sequences annotated as virus are subsequently queried against a customized nucleotide database (Polymicrobial NT DB) containing the Virus NT DB and representative RefSeq genomes from bacteria (1 per genus, *n* = 14,933), archaea (*n* = 511), fungi (*n* = 423), protozoa (*n* = 90), and plant (n = 145) genomes. These reference genomes represent a genomic “polymicrobial” community and cover a large amount of microbial sequence space. This allows for the removal of false-positive annotations from the first query using a relatively small reference database.

Taxonomic annotations are augmented using a modified version of the 2b lowest common ancestor (2b-LCA) algorithm described in [79]. The 2b-LCA algorithm provides conservative taxonomic assignments toward lower nodes of the tree when similarity is found across a heterogeneous collection of taxonomies. However, the LCA algorithm fails when crossing higher taxonomic ranks. For example, sequences with similarity to both bacterial and viral taxa have an LCA of “root” in the NCBI tree, while viruses from distinct viral domains (e.g., bacteriophage and vertebrate viruses) are assigned to “virus root.” Hecatomb will identify these instances and augment the annotations by reverting to the top-hit annotation. Each instance of this is flagged in the final output table so researchers can choose to include or exclude these from downstream analysis. This approach provides additional information about sequences with ambiguous taxonomic assignments instead of just leaving them as “root” or “virus root” and simply discarding them.

Sequence annotations from queries against both amino acid and nucleotide databases are combined into 1 table and assigned updated taxonomies using the most recent version of NCBI’s Taxonomy Database using TaxonKit [80,81] (Fig. 2A). This taxonomy table contains full Linnaean taxonomic lineages (Kingdom, Phylum, Class, Order, Family, Genus, and Species), alignment type used for annotation (translated [aa, amino acid database] or untranslated [nt, nucleotide database]) and LCA augmentation information. Due to Hecatomb keeping track of individual sequence IDs throughout this process, it is then possible to combine these read-based taxonomic assignments to other data generated by Hecatomb or external data resources (Fig. 2B). By default, Hecatomb will combine MMSeqs2 alignment information (e.g., target/query ID, e-value, percent identity, alignment length, etc.) and count table information gathered during the clustering process. As an example of combining data to external resources, Hecatomb will provide Baltimore virus type information (both Baltimore class and group). Baltimore is a classification system that places viruses into 1 of 7 groups depending on a combination of their nucleic acid (DNA or RNA), strandedness (single-stranded or double-stranded), sense, and method of replication. This could easily be extended to a variety of external data resources. Together, these disparate data tables are collected into Hecatomb’s bigtable.tsv. As Hecatomb tracks sample identifiers, it is then possible to combine the bigtable with sample data. All of these data are formatted to make them easily importable into commonly used data analysis tools for statistical and graphical analysis.

### Module 3: Assembly

**Figure 3:**
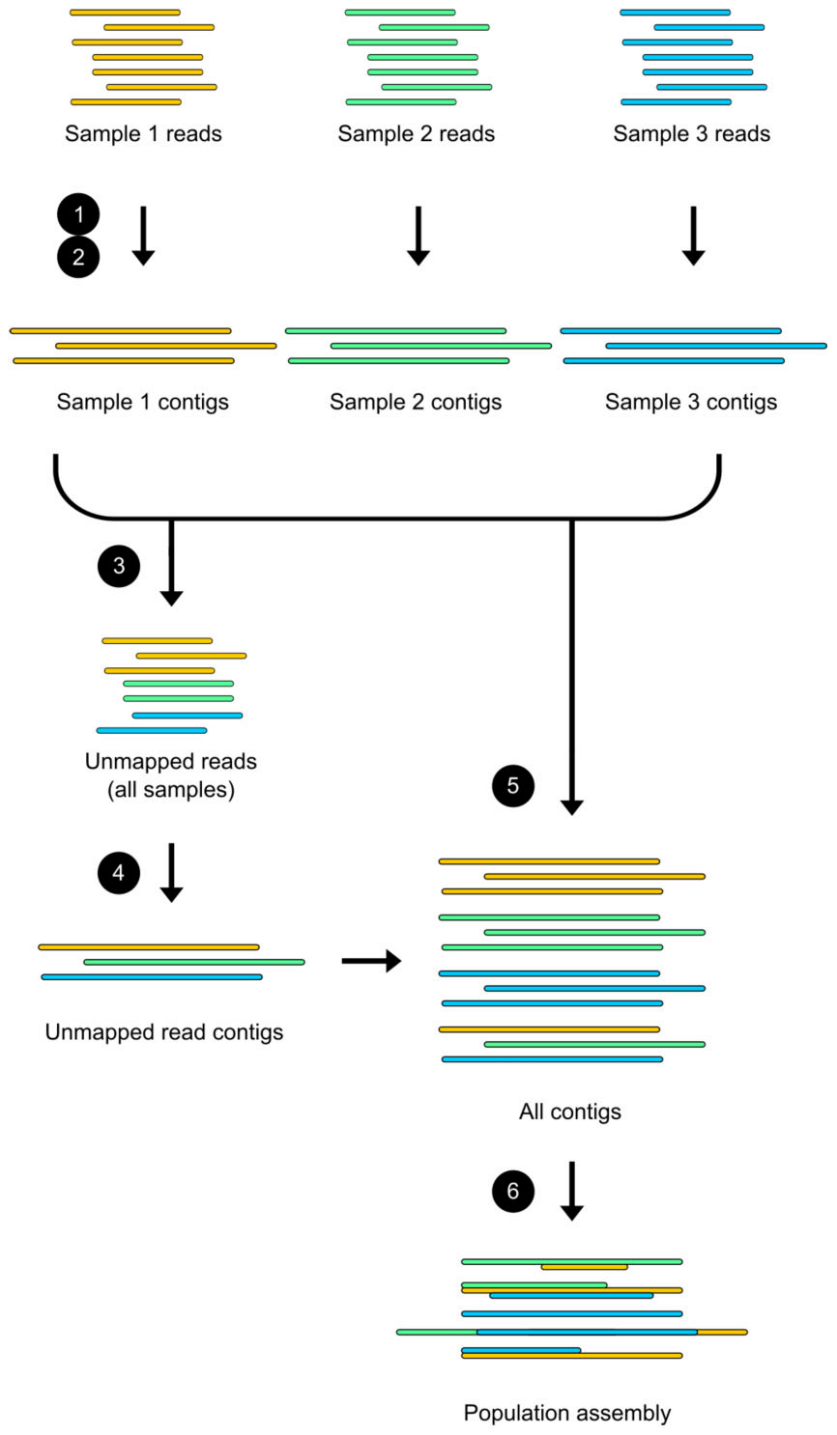
Viral metagenome merged assembly. (1) High-quality *k*-mer–normalized sequences from individual samples are assembled using either MEGAHIT or Canu. (2) The sequences for each sample are mapped to their respective assemblies. (3) The unmapped reads from all samples are pooled together. (4) The pooled unmapped reads are assembled using either MEGAHIT or Canu. (5) The contigs from all sample assemblies and the unmapped reads assembly are combined together. (6) Overlapping contigs are joined together using Flye using the subassemblies algorithm.

By default, Hecatomb performs an assembly for individual samples using MEGAHIT for short reads or Canu for long reads (Fig. 3) [82,83]. Individual sample assemblies are then merged into a population assembly using Flye [84].

Per sample contig abundances are calculated by mapping individual sample reads to the population assembly using BBMap [85]. Read counts are reported normalized to library size and contig length using a variety of measures (reads per kilobase million [RPKM], fragments per kilobase million [FPKM], and sequences per million [SPM]). SPM is the same calculation as used for transcripts per kilobase million (TPM) except that the sequences are not assumed to be transcripts [86,87]. Additional contig properties (e.g., length, GC content, coverage percentage) are combined with taxonomic assignments and sample abundance estimates into a final table (contig_count_table.tsv).

Options to skip the assembly step and to perform a cross-assembly are available for the user. Cross-assembly assembles all reads from all samples simultaneously (skipping the individual sample assemblies). This can result in better-quality assemblies but is computationally expensive for larger datasets and may not be an option for many users.

### Module 4: Contig-based annotation

Module 4 provides taxonomic annotations to contigs. Taxonomy is assigned to all contigs in the population assembly using MMseqs2 [49]. Each contig is queried against the same polymicrobial nucleotide database (Polymicrobial NT DB) used for read-based annotation (contigAnnotations.tsv). Additionally, information obtained from both the read-based annotation and assembly modules (Fig. 1) is combined. Read mapping information (start, stop, mapping quality, etc.) is maintained during the sample abundance estimation (mapping with BBMap) performed as part of Module 3 [85]. This mapping information is combined with read-based annotations (bigtable.tsv) to generate a new table combining read-based taxonomic information across each contig (contigSeqTable.tsv).

### Installation and dependency management

Hecatomb is hosted on GitHub [88] and is available for installation from Bioconda [89] and the Python Package Index [90], easing installation for individual users with a single command. Hecatomb makes liberal use of Conda environments to ensure portability, ease of installation, and proper versioning of software dependencies (Supplementary Fig. S2). All required and optional software dependencies are summarized in Supplementary Table S1 [49,71,74,81–85,91–94]. Hecatomb and Conda handle the installation of all dependencies. Conda environments for jobs are created automatically by Snakemake. The use of isolated Conda environments for Hecatomb minimizes package version conflicts, minimizes overhead when rebuilding environments for updated dependencies, and allows maintenance and customization of different Hecatomb versions.

While Hecatomb is a Snakemake pipeline, it uses the Snaketool command line interface to make running the pipeline as simple as possible [95]. Snaketool populates required file paths and configuration files, allowing Hecatomb to be configured and run with a simple command, and it offers a convenient way to modify parameters and customize options.

### High-performance computing deployment

Hecatomb can be deployed on a high-performance computing (HPC) cluster and can utilize Snakemake profiles for cluster job schedulers (e.g., Slurm, SGE, etc.). Snakemake uses profiles to submit pipeline jobs to the job scheduler and monitor their progress. Profiles can be created manually, but Hecatomb has been designed for compatibility with the official Cookiecutter [96] profiles for Snakemake [97] and comes with a preinstalled Slurm example profile.

### Customization

Hecatomb comes precompiled with many predefined settings regarding individual process options. These settings are highly customizable through the inclusion of a Snakemake YAML file. This file provides a single-source solution to user customization. Settings such as the quality threshold used for read trimming in Module 1 or the length of contig to maintain in Module 3 can easily be adjusted per an individual user or project needs.

## Application

### Hecatomb accelerates profiling of viral metagenomes

We reanalyzed a previously published dataset of 95 stool samples collected from simian immunodeficiency virus (SIV)–infected rhesus macaques (*Macaca mulatta*) (NCBI BioProject accession: PRJEB9503) [5]. Sequences were generated using the Illumina MiSeq 2 × 250 paired-end protocol using round A/B libraries (DNA and cDNA to enable detection of both RNA and DNA viruses) from virus-like particle preparations. These data contain sequences from a variety of RNA and DNA vertebrate viruses. The original study also identified a statistically significant difference in the abundance of several enteric viruses in SIV-infected animals compared to uninfected animals. These data included samples ranging from 768,268 to 4,229,134 raw input reads with a duplication rate at 97% identity ranging from 3.98% to 56.06% (Supplementary Table S2).

**Figure 4:**
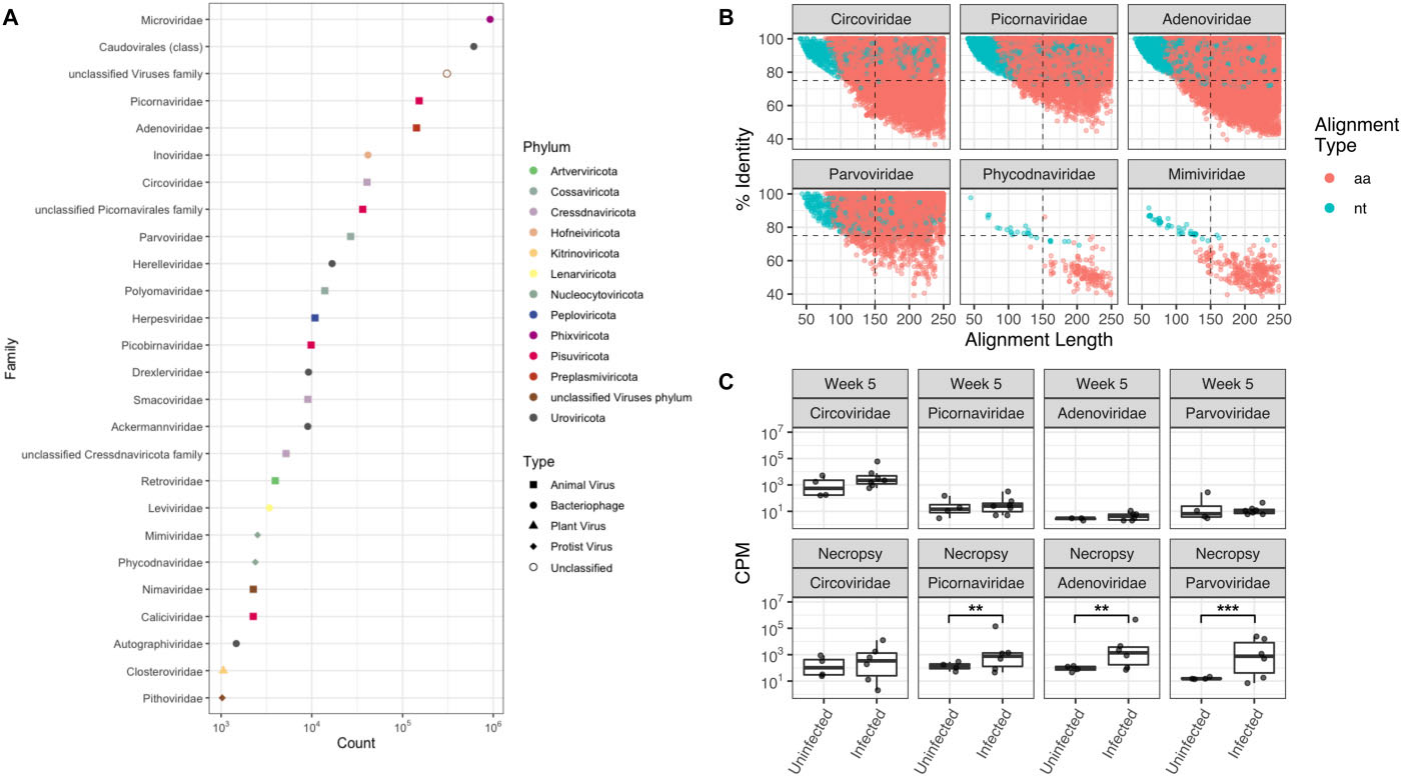
Reanalysis of rhesus macaque stool viromes. (A) Abundance of reads classified by viral phylum (color) and type (shape) from 2,394,740 input sequences (all annotated sequences from the entire study). Phyla represented by fewer than 1,000 reads were excluded. (B) Percent identity and alignment lengths of all sequences classified for the 4 animal viruses identified in the previous study and 2 viruses of protists. Horizontal (70% identity) and vertical (150-base alignment length) dashed lines indicate a user-defined quadrant space. Each point represents an individual sequence colored by classification method (aa = translated search to an amino acid database, nt = classified via an untranslated search to a nucleotide database). Panels A and B represent data obtained from all 95 samples in the study. (C) Comparison of the number of sequences in SIV-infected and uninfected samples 5 weeks postinfection with SIV and at the time of necropsy. Significance determined by the Wilcoxon signed-rank test. *P ≤ 0.05, **P ≤ 0.01, ***P ≤ 0.001, ^****^P ≤ 0.0001. CPM: counts per million.

We first assessed Hecatomb’s overall ability to detect diverse viral sequences. Hecatomb was executed using the round A/B preprocessing module and default parameters. Hecatomb classified sequences into phylogenetically diverse viral groups (Fig. 4A). Of the 2,394,740 reads annotated by Hecatomb as viral, 1,989,352 (83%) were annotated using queries against protein (aa) databases and 405,388 (17%) annotated using queries against nucleotide databases (nt). Bacteriophages from the family Microviridae and the order Caudovirales were highly abundant. Sequences belonging to a diverse set of viruses associated with infection of plants and protists were also detected (Supplementary Fig. S3). Similar to the original study, Hecatomb identified a large number of sequences belonging to the Picornaviridae and Adenoviridae. Sequences from these viral families were found to be more abundant in SIV-infected macaques when compared to uninfected animals in the original study (Fig. 4C).

Hecatomb collects and organizes alignment statistics (e.g., e-values, percent identity, alignment length, etc.) generated for each sequence annotation in Module 2. These data can be useful for assessing the prevalence and quality of viral annotations within a study. As an example, we examined the percent identity and alignment lengths of every read assigned taxonomy to the 4 families of viral enteropathogens identified in the original study (Circoviridae, Picornaviridae, Adenoviridae, and Parvoviridae) (Fig. 4B). Hecatomb annotated sequences for these 4 viral families using both translated queries to amino acid (aa) databases and untranslated queries to nucleotide (nt) databases. Quadrants were applied to visualize low- and high-identity and short- and long-alignment lengths for every annotated sequence. Sequences in the upper 2 quadrants are highly similar to sequences in the reference databases over short (upper left, quartile 1 [Q1]) or long (upper right, Q2) alignment lengths, while sequences in the lower 2 quadrants have low similarity over short (lower left, Q3) or long (lower right, Q4) alignment lengths. For this analysis, we arbitrarily selected 70% identity to represent the cutoff between low and high identity for translated (aa database) and 90% identity for untranslated (nt database) alignments. These values are adjustable and could be customized for each study and viral family of interest. Using this framework, it is clear that a majority of sequences are high identity (both short and long alignments) to sequences in both the aa and nt reference databases for the 4 families of enteropathogenic viruses.

In contrast, there were also a large number of sequences classified due to similarity to reference sequences from viruses of protists (Fig. 4B). Viruses in the family Mimiviridae infect *Acanthamoeba*, and those in the family Phycodnaviridae infect algae, and both are dsDNA viruses with large genomes [98]. While it is conceivable that these viruses may exist in the stool samples of rhesus macaques via water or food, using the quadrant framework, there is little evidence of high-identity alignments to any sequence in either the aa or nt databases (Fig. 4B, Supplementary Fig. S4). Hecatomb does not automatically remove sequences from these families due to their presence in environmental datasets. There is evidence for short and long low-identity alignments (quadrant 4) to both Phycodnaviridae and Mimiviridae reference sequences. Thus, these sequences should be analyzed using additional metrics (i.e., E-values, abundance across samples, etc.) to determine if these represent potentially novel viral sequences. This would not have been possible using stringent E-value filtering prior to data analysis.

### Reevaluation of existing environmental metagenomic datasets

We assessed Hecatomb’s ability to analyze nonhuman-associated viromes by processing a previously studied coral reef dataset (NCBI BioProject accession: PRJNA595374, SRA study number SRP237459) [99,100]. The dataset consists of whole-genome shotgun (WGS) metagenomic sequencing of both seawater and coral mucus from inner and outer sections of a Bermuda reef system. The original studies only considered bacterial metagenome-assembled genomes, which makes it an excellent candidate for generating new biological insights by characterizing the viruses of this previously published dataset. The original study identified statistically significant differences in bacterial compositions between the coral mucus and seawater microbiomes and the coral mucus microbiomes from the inner and outer reefs. All analysis was performed using output from the contig annotations provided by Hecatomb’s modules 3 and 4.

**Figure 5:**
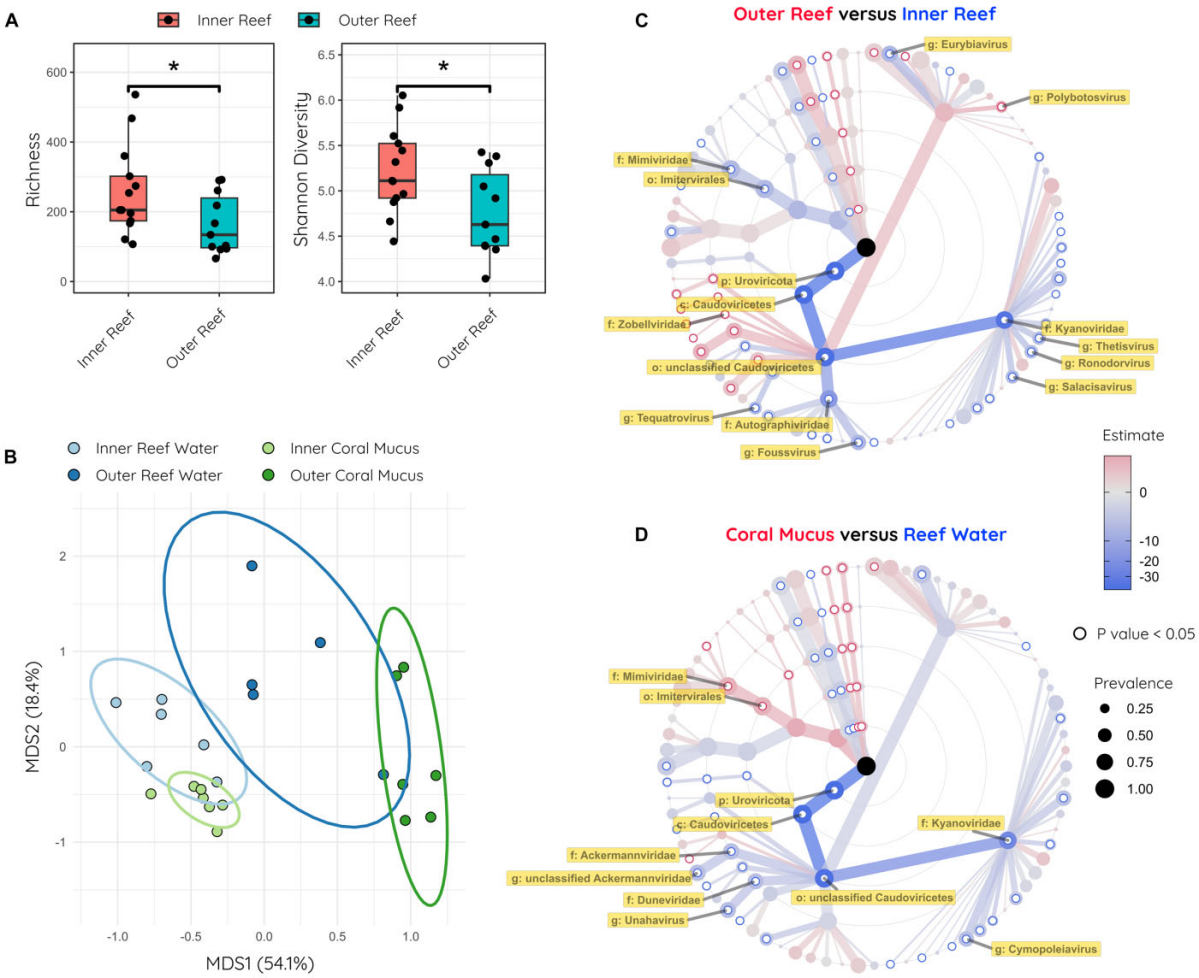
Reanalysis of coral reef metagenomes. (A) Viral species richness and Shannon diversity boxplots of inner and outer reef samples, colored by sample type. Significance (*P* < 0.05) is indicated. (B) Principal coordinates analysis (PCoA) of viral genera abundance. Inner and outer reef water samples are colored light blue and dark blue, respectively. Inner and outer coral mucus samples are colored light and dark green, respectively. Permutational multivariate analysis of variance (PERMANOVA) identified nonhomogenous distributions of inner versus outer reef samples (*P* = 0.001) and coral mucus versus reef water samples (*P* = 0.015). Ellipses for sample groups are drawn at 85% confidence levels for multivariate t-distribution. (C) Dendrogram of most prevalent viral taxa (>10% of samples). Linear regression (LR) models were generated for all taxa for the outer reef samples compared with the inner reef samples. Nodes are weighted by prevalence, and nodes and edges are colored by the LR model estimate coefficients. Significant LR models (*P* < 0.05) are indicated, and taxa with absolute coefficients greater than 3 are labeled. Nodes colored red are elevated in outer reef samples, whereas nodes colored blue are elevated in inner reef samples. (D) Same as for Fig. 5C except LR models are calculated for coral mucus samples compared with reef water samples. Nodes colored red are elevated in outer reef samples, whereas nodes colored blue are elevated in inner reef samples.

We first tested for differences in viral species alpha diversity using both Shannon diversity and richness comparing both inner and outer reef samples and coral mucus and reef water samples (Fig. 5). Both Shannon diversity and richness were significantly higher for inner reef samples compared to outer reef samples (Fig. 5A). Shannon diversity and richness were not significantly different between coral mucus and reef water. This result is in contrast with the bacterial diversity and richness metrics being similar across all samples as reported in the original study [99].

Next, we compared the viral compositions of these coral reef samples using beta diversity. Principal coordinate analysis (PCoA) of Bray–Curtis dissimilarity of viral genera, and permutational multivariate analysis of variance (PERMANOVA) showed nonhomogenous distributions between inner and outer reef samples (*P* = 0.001) as well as between coral mucus and reef water samples (*P* = 0.015) (Fig. 5B). There is a strong separation of inner and outer reef samples along the x-axis and a weaker separation of reef water and coral mucus samples along the y-axis; this is the same trend observed in the original study for the bacterial compositions.

Linear regression (LR) characterizes the viral taxa that are driving the differences between inner and outer reefs, as well as between coral mucus and reef water samples. The R package microViz will calculate LR models at all taxa and taxon levels for the various sample groups. We calculated LR models to the genus level and generated tree plots for all taxa with prevalence greater than 10% of samples, colored by the (LM) estimate coefficients, and weighted by prevalence between the inner and outer reef samples (Fig. 5C) and between the coral mucus and reef water samples (Fig. 5D). These analyses indicate that inner reef samples have significantly higher relative abundances of many viral taxa, including several Caudoviricete*s* taxa and especially the Kyanoviridae family, which consist of various Synechococcus phages and Cyanophages (Fig. 5C). Conversely, far fewer viral taxa were more abundant in outer reef samples. Reef water samples contained elevated abundances of many viral taxa compared to coral mucus samples, with the main exception of the family of giant viruses Mimiviridae (Fig. 5D).

### Accelerated discovery of novel viruses

**Figure 6:**
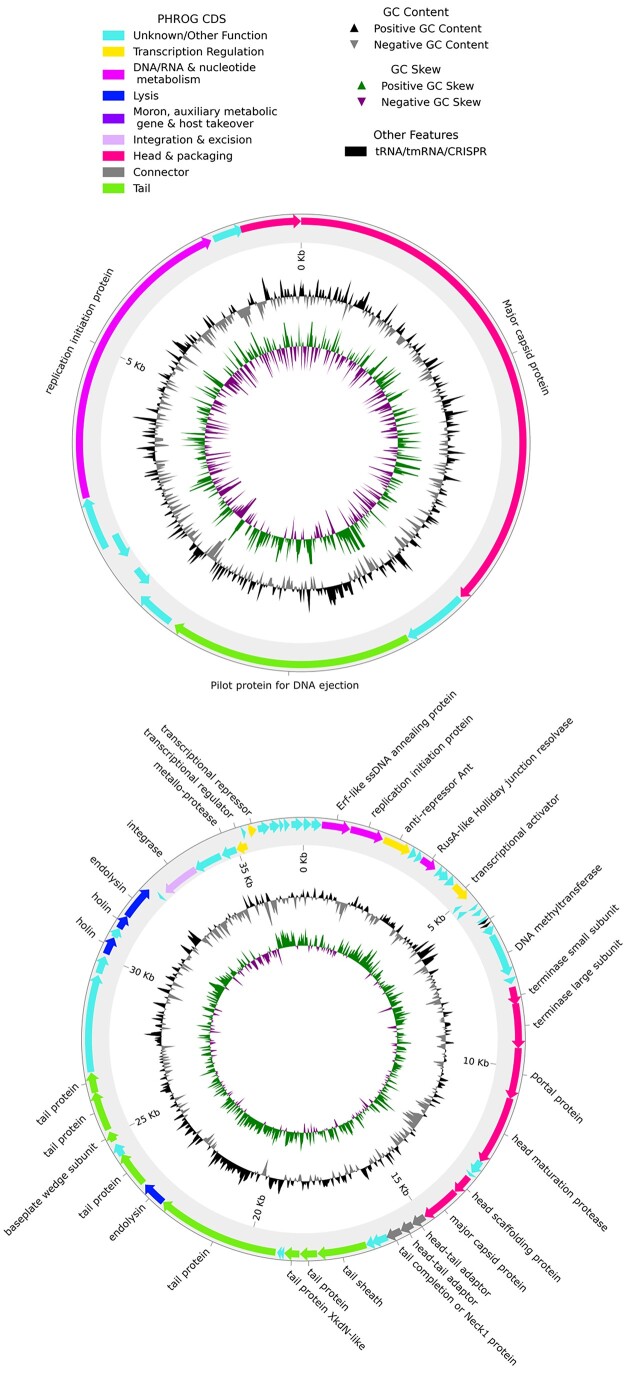
Circos plots of complete bacteriophage genomes. Circos plots were generated for all novel bacteriophage genomes using Phrokka’s pharokka_plotter.py script (circos plots available at 10.5281/zenodo.6388251). (A) Circos plot for uncultured Microviridae phage 977C1. (B) Circos plot for uncultured Caudoviricetes phage 1112C1.

Hecatomb retains the assembly graph as well as the assembly itself, which downstream tools can utilize to resolve metagenome-assembled genomes. There was strong evidence for the presence of novel bacteriophage within the SIV-macaque dataset in the form of many high-quality but low-identity alignments to known reference viruses (Supplementary Fig. S5). We therefore processed the assembly graph with Phables and identified 127 probable complete phage genomes [61]. Phables bins fragmented assemblies into complete genomes. These genomes were assessed with CheckV, which determined that 121 of them were high-quality complete phage genomes [101]. We assigned taxonomy using MMSeqs2 with the Hecatomb primary nucleotide databases (Supplementary Table S3) [49]. Lastly, the genomes were annotated using Pharokka [102]. Of the 121 genomes, 98 were Microviridae (Supplementary Table S3). Of these Microviridae, 96 exhibited the hallmark replication initiation protein followed by a major capsid protein, and a further 55 had the hallmark minor tail or pilot tail spike protein (Fig. 6A) while the other 41 contained hypothetical proteins where the tail spike protein would be. There were 10 Caudoviricetes, 2 Cressdnaviricota genomes, and 8 that had hits to known phages with no taxonomic information. There were 13 cases where 2 genomes were resolved from the same assembly graph element and likely represent quasi-species that can occur, for instance, through recombination events [103,104]. We also processed the coral dataset using the same method. Synteny was also conserved in these larger phages; for instance, Caudoviricetes phage 1112C1 arranged its capsid and tail proteins together and exhibited the conserved layout described in [105] (Fig. 6B). In the coral dataset, we identified 3 complete Caudoviricetes phage genomes (Supplementary Table S3). The number of samples in this study was much lower and likely impacted the number of recovered viral genomes. The recovered viral genomes across both studies are novel, with only 18 aligning to a known phage with an identity higher than 90% (Supplementary Table S3). This demonstrates Hecatomb’s utility for data-mining published environmental metagenome projects to generate novel viral genomes.

### Analysis of an *in silico* set of viral genomes

To determine the ability of Hecatomb to accurately identify viral sequences in a mixed metagenome, we generated a mock *in silico* data set. Canonical viral genomes from each genomic type (single- and double-stranded DNA and RNA genomes and 1 retrovirus) were downloaded from NCBI (Table 1). For each genome, we simulated 1 × 10^4^ 250 base-pair sequences using the Illumina error-model model available in InSilicoSeq [106]. Next, we generated 1 × 10^6^ sequences from a collection of 1,520 cultivated human gut bacterial genomes [102] using the same Illumina error model. Simulated sequences from viruses and bacteria were combined to simulate a mixed virus–bacteria environment and processed through Hecatomb using the default settings. Hecatomb assigned the correct taxonomy to 99.64–99.65% of the simulated viral reads with a sensitivity ranging from 0.98–1 and a false-positive rate ranging from 8.8 × 10^−4^ to 1.35 × 10^−3^ (Table 1).

**Table 1.** Analysis of an *in silico* generated mock viral–bacterial community

Virus	NCBI accession	Genome type	Genome length (bp)	True positive	False positive	Sensitivity	False-positive rate
**Human gammaherpesvirus 4**	NC_007605.1	dsDNA	171,823	9,910	1,345	0.99	1.35 × 10^−3^
**Human parvovirus B19**	NC_000883.2	ssDNA	5,596	9,964	873	1	8.8 × 10^−4^
**Rotavirus**	NC_011507:0-10	dsRNA	18,562	9,856	134	0.99	1.35 × 10^−4^
**Rabies**	NC_001542.1	ssRNA	11,932	9,932	654	0.99	6.6 × 10^−4^
**Human immunodeficiency virus 1**	NC_001802.1	retrovirus	9,181	9,876	564	0.99	5.69 × 10^−4^
**Crassphage**	NC_055760	dsDNA	94,878	9,765	4578	0.98	4.58 × 10^−3^

## Discussion

Virome sequencing is the premier approach to evaluate the viral content of both host-derived and environmental samples. It is useful for determining what types of viruses are present in individual samples and how virome compositions compare between sample groups. This information forms the foundations for answering a wide array of interesting biological questions. For example, virome composition has recently been analyzed as an indicator of the microbial impacts of climate change [107,108]. Virome sequencing was also critical for the discovery and characterization of SARS-CoV-2 in 2019 [109]. Effective characterization of virome sequencing data requires rigorous and integrated software platforms to facilitate and accelerate virus discovery and virome compositional analysis. Given these tools, researchers will be better prepared to assess how viruses are associated with some of the most important challenges to human life today.

All virome studies are dependent on effective computational tools to identify and classify viral reads or assembled contigs within a metagenome. Viral metagenomics is often dependent on identifying sequence similarity against reference sequence databases, either directly via homology-based searchers or using machine learning techniques that have been trained on reference databases to identify features unique to viral sequences (reviewed in [110]). Homology-based searches can take a “brute-force” approach, wherein all unclassified sequences are queried against a comprehensive, multikingdom reference sequence database (e.g., NCBI nt or nr). This approach relies on the search algorithm (e.g., BLAST, DIAMOND [48]) to pick the best or lowest-common ancestor of a group of hits to provide a final taxonomic assignment to an unknown query sequence. This approach is slow and requires significant computational resources, which is why Hecatomb takes an alternate approach. First, it captures all “potentially viral” sequences initially querying a small viral sequence database. The “potentially viral” sequences typically represent a fraction of the full metagenomic data, making subsequent computation more tractable. To confirm viral taxonomic assignment, potentially viral sequences are cross-checked against a curated small transkingdom reference database containing genomic representatives from all kingdoms of life. Hecatomb completes this iterative search approach using translated searches against amino acid databases as well as untranslated searches against nucleotide databases, combining the results of each to ensure detection of viral sequences is database independent. This iterative search strategy uses database orders of magnitude smaller than comprehensive, multikingdom databases (such as NCBI’s nt and nr) increasing computational efficiency without limiting viral detection.

Hecatomb’s design philosophy recognizes that there are no “perfect” databases or search algorithms. Both the brute-force and iterative search approaches against comprehensive or curated databases will result in different rates of true/false positives/negatives. Instead, Hecatomb relies on providing a compiled and rich set of data for search result evaluation. We used this strategy to reassess the virome composition of SIV-infected and uninfected rhesus macaques [5]. The original study used an iterative approach but relied on comprehensive, transkingdom databases (NCBI nt and nr) and identified associations between 4 families of animal viruses (Circoviridae, Picornaviridae, Adenoviridae, and Parvoviridae) and SIV infection. The new Hecatomb transkingdom database is 6 orders of magnitude smaller than GenBank nt (5.0 × 10^6^ versus 1.3 × 10^12^), which results in a significant reduction in computational time and resources. Hecatomb identified the same 4 viral families and their relationship to SIV-mediated disease. Similar to our analysis of these samples using Hecatomb, the original study also classified a number of sequences to the Mimiviridae and Phycodnaviridae. Statistical comparison of these sequences between groups (e.g., SIV infected vs. uninfected) did not reveal any significant associations, and thus they were not discussed further. However, new evaluation of results from Hecatomb indicates that there were likely false-positive classifications reported in the original analysis. Lastly, we identified 121 novel complete phage genomes in this dataset. The majority of these genomes were Microviridae, which was the most abundant family by read abundance in the dataset. Recovering these genomes with a provisional taxonomic classification using Hecatomb’s annotations is simple, fast, and scalable. This method is not a replacement for culturing and characterizing viruses in a lab environment. However, these *in silico* methods are essential when considering the scale of the viral dark matter problem. This reanalysis highlights how coordinated data such as alignment statistics and taxonomy can be powerful tools for virome evaluation and novel virus discovery.

Hecatomb was able to effectively evaluate the viromes of environmental (nonhost-associated) viromes. Leveraging the R packages phyloseq and microViz allowed us to quickly and easily complete the analysis in approximately 200 lines of code [66,67]. This analysis was primarily designed to identify compositional changes in viromes between reef types (inner or outer) and within coral mucosa and the surrounding water from a previously published metagenomic data set [99,100]. The original study identified elevated levels of Pelagibacter, Synechococcus, and unclassified Rickettsiales in inner reef samples compared to outer reef samples. Indeed, we found Synechococcus phages and Cyanophages were important for distinguishing the highly fluctuating inner reef system from the thermally stable outer reef. The authors showed that the bacterial microbiomes were unique for inner and outer reefs for both coral and mucus samples. We applied the same method on the viral compositions and confirmed that the viromes for these 4 sample types are also all unique.

Interestingly, viral species richness and diversity were significantly elevated in inner reef samples, whereas the original study found these to be similar in terms of bacterial composition. Viral activity is an important vehicle for nutrient cycling, which was thought to be much higher in the inner reef based on the metabolic profiles of these samples [99]. There were many viral taxa that were more abundant in the inner reef samples and few that were elevated in the outer reef. Similarly, many viruses were more abundant in reef water samples than coral mucosa, with the main exception of the giant viruses from the family Mimiviridae. It would be interesting to elucidate whether the coral mucus is impacting viral infectivity directly or if the phages are switching to a lysogenic rather than lytic life cycle in this environment.

Corals occasionally shed their mucosa. Shedding occurs far more frequently in the inner reef due to stressors such as thermal fluctuation and sedimentation from surface runoff. The increased flux of nutrients and microbes from corals to the surrounding reef water may be contributing to increased microbial and viral activity in inner reef samples compared to the outer reef. Different concentrations of microbes might also be having an impact. The outer reef systems are subject to upwelling, resulting in greater exchange of water with the open ocean, which is probably flushing microbes and viruses from the environment. Unfortunately, it is not possible to infer microbial concentrations from WGS sequencing. The reanalysis of this coral dataset has generated many new hypotheses about viral host interactions within a coral reef system.

### Potential implications

Virome analysis is complex and requires efficient computational tools to generate analyst-friendly results. Hecatomb provides a comprehensive and computationally efficient solution for both read- and assembly-based viral annotation, virome analysis, and novel virus discovery. The pipeline is delivered with a convenient and easy-to-use front end and is compatible with different sequencing technologies. Hecatomb’s comprehensive collection of data throughout the pipeline’s execution, in particular the collection of alignment statistics, empowers the identification and interrogation of viral taxonomic assignments. We demonstrate Hecatomb’s utility for rapid processing and analysis of viral metagenomes with a well-studied validation gut viral metagenome dataset. We also demonstrate its utility for mining regular metagenome samples for virome analysis by analyzing an existing environmental dataset. Virome analysis is an evolving field requiring novel approaches for comprehensive characterization. For example, none of the described workflows account for spliced coding sequences (CDS), hampering the automation of viral genome annotation. The modularity of Hecatomb’s approach should enable easy integration of novel methods as they emerge.

### Methods

All commands used for analyzing the Hecatomb annotations are available as a gist on GitHub [111].

#### Reevaluation of the SIV dataset

We reanalyzed a previously published data set of 95 samples obtained from stool samples collected from SIV-infected rhesus macaques (*Macaca mulatta*) (NCBI BioProject accession: PRJEB9503) [5]. Sequence data were generated using the Illumina MiSeq 2 × 250 paired-end protocol on libraries of total nucleic acid (DNA and cDNA to enable detection of both RNA and DNA viruses). Hecatomb was executed using the round A/B preprocessing module and otherwise default parameters. Data were analyzed in R with Tidyverse [64]; commands are available in the above GitHub gist.

#### Reevaluation of coral microbiomes

We reanalyzed a coral reef dataset (NCBI BioProject accession: PRJNA595374, SRA study number SRP237459) [99,100] of WGS metagenomic sequencing (Illumina MiSeq, paired 2 × 250) of both seawater and coral mucus from inner and outer sections of a Bermuda reef system. Hecatomb was run with fast search parameters, cross-assembly, and otherwise default parameters. Data were analyzed in R with PhyloSeq [66] and MicroViz [67]; commands are available in the above GitHub gist.

#### Identification of phage genomes from Hecatomb assemblies

To identify complete phage genomes, the assembly graphs created by Hecatomb were processed with Phables [61]. The predicted phage genomes were assessed with CheckV [101]. High-quality and complete phage genomes were assigned provisional taxonomic annotations using MMSeqs2 with the Hecatomb viral amino acid database (easy taxonomy) and viral nucleotide database (easy-search plus TaxonKit). Lastly, the genomes were annotated with Pharokka [102].

## Supplementary Material

giae020_GIGA-D-23-00206_Original_Submission

giae020_GIGA-D-23-00206_Revision_1

giae020_GIGA-D-23-00206_Revision_2

giae020_Response_to_Reviewer_Comments_Original_Submission

giae020_Response_to_Reviewer_Comments_Revision_1

giae020_Reviewer_1_Report_Original_Submission Arvind Varsani -- 9/8/2023 Reviewed

giae020_Reviewer_2_Report_Original_Submission Satoshi Hiraoka -- 9/10/2023 Reviewed

giae020_Reviewer_2_Report_Revision_1 Satoshi Hiraoka -- 2/2/2024 Reviewed

giae020_Supplemental_Files

## Data Availability

The reanalysis with Hecatomb utilized preexisting datasets, which are available under the NCBI BioProject accessions PRJEB9503 for the macaque SIV dataset [5] and PRJNA595374 (SRA: SRA study number SRP237459) for the coral reef dataset [99,100]. Accessions for novel phage genomes identified in this study are available in Supplementary Table S3. An archival copy of the code and supporting data is also available via the *GigaScience* repository, GigaDB [113]. Hecatomb is registered on WorkflowHub [114].

## Additional Files

**Supplementary Fig. S1**. Features and useability of popular viral metagenomics software as of March 2023. “Packaged install” indicates that the software is installable from any package manager or Apptainer. Software is considered “recently updated” if it has been updated within the last 12 months. Software has been selected from a community-driven compilation of viral bioinformatics tools (an archived version is available at 10.5281/zenodo.6388251).

**Supplementary Fig. S2**. Implementation of Hecatomb using Snaketool, Snakemake, and Conda. Hecatomb takes in command line arguments, data, and configuration parameters and outputs both results for analysis and run information. Hecatomb interacts with the job scheduler in high-performance computing (HPC) environments. Hecatomb distributes individual tasks to the job queue. Command line arguments, *gray*; files, *yellow*; Conda environments, *blue*; scripts/programs, *green*; workload manager, *pink*.

**Supplementary Fig. S3**. Taxonomic subsets of virus types. Viral families present in the 95-sample SIV reanalysis study (A) plant viruses and (B) protist viruses (C) is supposed to be (B).

**Supplementary Fig. S4**. Sequence per quadrant evaluation. Percentage of reads per quadrant in Fig. 5. (A) Translated (aa reference database) and (B) untranslated (nt reference database).

**Supplementary Fig. S5**. Alignments of bacteriophage sequences from rhesus macaque stool viromes. Alignment lengths and percent identities are shown in separate plots for the bacteriophage orders Caudovirales, Levivirales, Petitvirales, and Tubulavirales. Alignments are colored by viral family.

## Availability of Source Code and Requirements

Project name: Hecatomb

Project homepage: github.com/shandley/hecatomb [88]

Project documentation: hecatomb.readthedocs.io [112]

Operating system: Linux

Programming language: Python

Other requirements: Conda or pip

License: MIT

RRID: SCR_025002

Bio.tools ID: hecatomb

Restrictions to use by nonacademics: None

## Abbreviations

AIDS: acquired immunodeficiency syndrome; ANOVA: analysis of variance; CPM: counts per million; FPKM: fragments per kilobase million; HPC: high-performance computing; ICTV: International Committee on Taxonomy of Viruses; LCA: lowest common ancestor; NCBI: National Center for Biotechnology Information; PERMANOVA: permutational analysis of variance; PCoA: principal coordinate analysis; RPKM: reads per kilobase million; SIMPER: similarity percentage; SIV: simian immunodeficiency virus; SPM: sequences per million; WGS: whole-genome shotgun.
