# Supplementary material for: Hecatomb: an integrated software platform for viral metagenomics
Source: Gigascience. 2024 Jun 4;13:giae020. doi: 10.1093/gigascience/giae020 (PMC11148595; doi:10.1093/gigascience/giae020)
Supplement: giae020_GIGA-D-23-00206_Revision_2 [file giae020_giga-d-23-00206_revision_2.pdf]

GigaScience

Hecatomb: An Integrated Software Platform for Viral Metagenomics

--Manuscript Draft--

**Manuscript Number:**	GIGA-D-23-00206R2
**Full Title:**	Hecatomb: An Integrated Software Platform for Viral Metagenomics
**Article Type:**	Technical Note
**Funding Information:**	National Institute of Diabetes and Digestive and Kidney Diseases (DK116713) — Dr. Scott Handley<br>Division of Intramural Research, National Institute of Allergy and Infectious Diseases (AI151810) — Dr. Scott Handley
**Abstract:**	Background<br>Modern sequencing technologies offer extraordinary opportunities for virus discovery and virome analysis. Annotation of viral sequences from metagenomic data requires a complex series of steps to ensure accurate annotation of individual reads and assembled contigs. In addition, varying study designs will require project specific statistical analyses.<br>Findings<br>Here we introduce Hecatomb, a bioinformatic platform coordinating commonly used tasks required for virome analysis. Hecatomb processes metagenomic data obtained from both short and long read sequencing technologies, providing annotations to individual sequences and assembled contigs. Results are provided in commonly used data formats useful for downstream analysis. Here we demonstrate the functionality of Hecatomb through the reanalysis of a primate enteric and a novel coral reef virome.<br>Conclusion<br>Hecatomb provides an integrated platform to manage many commonly used steps for virome characterization including rigorous quality control, host removal and both read- and contig-based analysis. Each step is managed using the Sankemake workflow manager with dependency management using Conda. Hecatomb outputs several tables properly formatted for immediate use within popular data analysis and visualization tools enabling effective data interpretation for a variety of study designs. Hecatomb is hosted on GitHub at github.com/shandley/hecatomb and is available for installation from the Bioconda at anaconda.org/bioconda/hecatomb and pypi.org/project/hecatomb/.
**Corresponding Author:**	Scott Handley, Ph.D.<br>Washington University in St Louis School of Medicine<br>St. Louis, MO UNITED STATES
**Corresponding Author Secondary Information:**	
**Corresponding Author's Institution:**	Washington University in St Louis School of Medicine
**Corresponding Author's Secondary Institution:**	
**First Author:**	Scott Handley, Ph.D.
**First Author Secondary Information:**	
**Order of Authors:**	Scott Handley, Ph.D.
	Michael J Roach
	Sarah J Beecroft
	Kathie A Mihindukulasuriya
	Leran Wang
	Anne Paredes
	Luis Alberto Chica Cardenas
	Kara Henry-Cocks

Powered by Editorial Manager® and ProduXion Manager® from Aries Systems Corporation

	Lais Farias Oliveira Lima
	Elizabeth A Dinsdale
	Robert A Edwards
**Order of Authors Secondary Information:**	
**Response to Reviewers:**	Please address the following points:<br><br>1) Please include a citation to your new GigaDB dataset (including the DOI link) to your reference list, and cite this in the data availability section.<br><br>The citation is:<br><br>[xx] Roach MJ; Beecroft SJ; Mihindukulasuriya KA; Wang L; Paredes A; Chica Cardenas LA et al. Supporting data for "Hecatomb: An Integrated Software Platform for Viral Metagenomics" GigaScience Database. 2024 http://dx.doi.org/10.5524/102506<br><br>In the data availability section, please write something along the lines, "An archival copy of the code and supporting data is also available via the GigaScience repository, GigaDB [xx]".<br><br>-- DONE<br><br><br>2) Please register your new software application in the bio.tools and SciCrunch.org databases to receive RRID (Research Resource Identification Initiative ID) and biotoolsID identifiers, and include these in your manuscript (in the "availability" section, below the "licence " line). This will facilitate tracking, reproducibility and re-use of your tool.<br><br>-- DONE<br><br>3) Please move in-text URLs of code and data sources to the bibliography and cite them by number in the text (similar to the GigaDB reference) - please refer to our citation guidelines. - e.g. lines 131, 216, 217, 233, 234<br><br>-- DONE<br><br>4) please add ORCIDs to the title page. We have the following on our files:<br>ORCID iDs: Scott Handley [0000-0002-2143-6570]; Michael J Roach [0000-0003-1488-5148]; Sarah J Beecroft [0000-0002-3935-2279]; Kathie A Mihindukulasuriya [0000-0001-9372-3758]; Leran Wang [0009-0003-6928-9893]; Anne Paredes [0009-0009-1881-3855]; Luis Alberto Chica Cardenas [0009-0007-7408-9385]; Kara Henry-Cocks [0009-0004-5888-9372]; Lais Farias Oliveira Lima [0000-0002-7616-3637]; Elizabeth A Dinsdale [0000-0002-2177-203X]; Robert A Edwards [0000-0001-8383-8949];<br><br>-- DONE<br><br>5) Please check for minor typos etc. (I noticed "sankemake workflow" in the abstract, line 18.)<br><br>-- DONE
**Additional Information:**	
**Question**	**Response**
Are you submitting this manuscript to a special series or article collection?	No
**Experimental design and statistics**	Yes

Powered by Editorial Manager® and ProduXion Manager® from Aries Systems Corporation

Full details of the experimental design and statistical methods used should be given in the Methods section, as detailed in our Minimum Standards Reporting Checklist. Information essential to interpreting the data presented should be made available in the figure legends.<br><br>Have you included all the information requested in your manuscript?	
**Resources**<br><br>A description of all resources used, including antibodies, cell lines, animals and software tools, with enough information to allow them to be uniquely identified, should be included in the Methods section. Authors are strongly encouraged to cite Research Resource Identifiers (RRIDs) for antibodies, model organisms and tools, where possible.<br><br>Have you included the information requested as detailed in our Minimum Standards Reporting Checklist?	Yes
**Availability of data and materials**<br><br>All datasets and code on which the conclusions of the paper rely must be either included in your submission or deposited in publicly available repositories (where available and ethically appropriate), referencing such data using a unique identifier in the references and in the "Availability of Data and Materials" section of your manuscript.<br><br>Have you have met the above requirement as detailed in our Minimum Standards Reporting Checklist?	Yes

Powered by Editorial Manager® and ProduXion Manager® from Aries Systems Corporation

Manuscript

Click here to access/download;Manuscript;Hecatomb_for_production.docx

Hecatomb: An Integrated Software Platform for Viral Metagenomics

Michael J. Roach[1,2,3], Sarah J. Beecroft[4], Kathie A. Mihindukulasuriya[5,6], Leran Wang[5,6], Anne Paredes[5], Luis Alberto Chica Cárdenas[5,6], Kara Henry-Cocks[1], Lais Farias Oliveira Lima[7], Elizabeth A. Dinsdale[1], Robert A. Edwards[1], Scott A. Handley[5,6*]

1) Flinders Accelerator for Microbiome Exploration, Flinders University, Adelaide, SA, Australia

2) Adelaide Centre for Epigenetics, University of Adelaide, Adelaide, SA, Australia

3) South Australian Immunogenomics Cancer Institute, University of Adelaide, Adelaide, SA, Australia

4) Harry Perkins Institute of Medical Research, Perth, WA, Australia

5) Department of Pathology & Immunology, Washington University School of Medicine, St. Louis, MO, USA

6) The Edison Family Center for Genome Sciences & Systems Biology, Washington University School of Medicine, St. Louis, MO, USA

7) Biology Department, San Diego State University, San Diego, CA, USA

ORCID IDs: Michael J Roach [0000-0003-1488-5148]; Sarah J Beecroft [0000-0002-3935-2279]; Kathie A Mihindukulasuriya [0000-0001-9372-3758]; Leran Wang [0009-0003-6928-9893]; Anne Paredes [0009-0009-1881-3855]; Luis Alberto Chica Cardenas [0009-0007-7408-9385]; Kara Henry-Cocks [0009-0004-5888-9372]; Lais Farias Oliveira Lima [0000-0002-7616-3637]; Elizabeth A Dinsdale [0000-0002-2177-203X]; Robert A Edwards [0000-0001-8383-8949]; Scott Handley [0000-0002-2143-6570]

**Address correspondence to shandley@wustl.edu*

Keywords: virome; virus discovery; bioinformatic workflow; viral metagenomics

ABSTRACT

Background

Modern sequencing technologies offer extraordinary opportunities for virus discovery and virome analysis. Annotation of viral sequences from metagenomic data requires a complex series of steps to ensure accurate annotation of individual reads and assembled contigs. In addition, varying study designs will require project specific statistical analyses.

Findings

Here we introduce Hecatomb, a bioinformatic platform coordinating commonly used tasks required for virome analysis. Hecatomb means "a great sacrifice". In this setting, Hecatomb is "sacrificing" false-positive viral annotations using extensive quality control and tiered-database searches. Hecatomb processes metagenomic data obtained from both short and long read sequencing technologies, providing annotations to individual sequences and assembled contigs. Results are provided in commonly used data formats useful for downstream analysis. Here we demonstrate the functionality of Hecatomb through the reanalysis of a primate enteric and a novel coral reef virome.

Conclusion

Hecatomb provides an integrated platform to manage many commonly used steps for virome characterization including rigorous quality control, host removal and both read- and contig-based analysis. Each step is managed using the Snakemake workflow manager with dependency management using Conda. Hecatomb outputs several tables properly formatted for immediate use within popular data analysis and visualization tools enabling effective data interpretation for a variety of study designs. Hecatomb is hosted on GitHub (github.com/shandley/hecatomb) and is available for installation from Bioconda and PyPI.

BACKGROUND

Viruses are also the most dominant entity on the planet with current global estimates as high as 10^{31} viral particles [1], and they are omnipresent in all cellular life forms [2]. As such, they exert significant influence on their surroundings. Metagenomic sequencing offers a powerful tool for studying viral diversity in both

host-associated and environmental systems [3–13]. However, there are currently many challenges associated with viral metagenomics. While viruses are the most abundant and diverse biological entity on the planet, they represent a minority of reference genomes in GenBank, largely due to difficulties associated with studying them [14]. There is a vast amount of sequence information that remains taxonomically or functionally ill-defined. These sequences are regularly referred to as “viral dark matter” and pose a significant barrier to annotating viral sequences from metagenomic data (reviewed in [15]). In addition, researchers must contend with how to annotate viral sequences from viruses with either RNA or DNA-based genomes. Our ability to successfully annotate metagenomic data as “viral” is directly impacted by the size and diversity of the reference database and the sensitivity of our search algorithms. Larger, more diverse reference databases can improve viral sequence annotation but are less conducive to high-sensitivity search algorithms required to identify distant sequence similarity. This dichotomy can force researchers to choose between optimal databases and search algorithms.

Another challenge to the interpretation of reference-based sequence annotation is that viral metagenomes are often plagued with false positive classifications [16–18]. Viruses share regions of sequence similarity with all other domains of life, including ‘stolen’ genes incorporated from their hosts’ genomes, and repetitive or low-complexity insertion elements or transposons. These sequences are present in many reference databases and can result in false classifications due to shared sequence similarity across taxonomies. The presence of false-positive classifications may influence data interpretation. For instance, the misclassification of viral sequences in clinical samples could lead to incorrect hypotheses about virus-disease associations or patient diagnosis. Similarly, an increased false-positive rate in any environment could lead to overestimates of species diversity. Highly-curated databases may alleviate false positives, but they require tremendous resources and time. Likewise, they risk missing newly discovered viruses that have yet to make their way through the curation process. Thus, it is important for bioinformatic tools to provide a system to classify the quality of similarity-based annotations in light of imperfect databases.

Numerous bioinformatics tools exist for identifying viral sequences from metagenomic data [19–44]. However, many of these are lacking in features or fail to offer researchers an end-to-end solution to manage the myriad tasks required of virome analysis (e.g., quality control, host removal, assembly). For example,

few tools are designed for read-based annotation of viral sequences and none are currently maintained [45,46]. Individual read annotations are valuable as detecting a small number of viral reads could signify the presence of a virus. This is true for viral sequences both closely related to and divergent from reference viral sequences. Detection of divergent viral sequences requires sensitive alignment-based tools such as BLAST, DIAMOND or MMSeqs2 [47–49]. Alternative approaches, such as those that use k-mer distances have also been implemented [50–53]. K-mer based algorithms are fast but limited in their ability to annotate viral sequences divergent from those in reference databases. Therefore, sensitive alignment based approaches are preferred for detecting divergent or novel viruses.

Alignment-based approaches are computationally more expensive than k-mer based approaches, but can be effectively implemented using a tiered database query approach [45,54]. In the tiered approach initial queries are made against small virus-only sequence databases. Subsequent secondary cross-checking against reference databases representing sequences from all domains of life are required to remove false-positive viral annotations. In addition, queries against both amino acid and nucleotide databases may be of interest. Reference sequences from viral taxa may only be represented in one or the other database types and amino acid databases will not include non-coding viral sequences. Tiered alignment-based queries across multiple databases require a number of steps and produce a series of disconnected outputs. A robust system to monitor and manage these steps and coordinate the outputs into a tractable framework would permit researchers to focus on making biological insights instead of on job and file management.

While read based annotation can provide sensitive and specific detection of viral sequences within a metagenome, metagenome assembled contigs can provide additional layers of information such as gene content, gene order and metabolic pathway prediction. Many of the tools used to assemble viral contigs are the same ones used for assembling bacterial contigs [55–58]. More recently, metavirome-specific assembly tools have begun to emerge with promising results [59,60]. Virome metagenomics is evolving beyond binning with algorithms specifically designed to resolve complete genomes from metavirome assemblies [61]. Similar to the requirements of read based annotations, assembly requires multiple steps to ensure high-quality data for downstream analysis.

Once contigs are obtained they can be further analyzed using one of many established virome analysis tools [23,28,31,43]. Each of these tools provides distinct information about the viral content of a metagenome. For example, VirSorter2, Cenote-Taker 2 and geNomad use customized classifiers and curated Hidden Markov Models (HMM) to estimate the “viraleness” of metagenome assembled contigs. This is useful for separating viral from non-viral contigs, but additional steps are required to assign taxonomic lineages. vContact2 can assign genus-level taxonomy using gene-sharing networks to prokaryotic but not eukaryotic viruses. VIBRANT uses deep learning neural networks to classify prokaryotic viral contigs. Numerous additional tools are also available for researchers to mine taxonomic and functional information from a metagenome [62]. This complex array of tools for virome interrogation provides a number of opportunities for virome researchers. However, each of them is dependent on the generation of high-quality input assembly data from a wide range of experimental systems, and they vary greatly in terms of useability and support. A common workflow that takes inputs from both short and long reads and emphasizes rigorous quality control to remove non-biological contamination and host from a variety of library types and study designs would ensure researchers provide the highest quality of data to each of these tools regardless of their experimental system.

While several options are available for virome analysis using read or contig based approaches, integrating these results with study data is critical for making biological insights. Project specific statistical analyses are often required. For example, statistical models to test if a pathogenic virus is associated with a disease using sparse read-based results will differ wildly from a study analyzing bacteriophage ecology. Fortunately, the vast majority of these disparate statistical approaches are available as R software packages [63]. In addition, principles guided by the popular tidyverse and ggplot2 packages are familiar to many researchers [64,65]. These principles have been successfully applied to the analysis of bacterial microbiome data in software suites such as PhyloSeq and microViz, but have yet to be applied to the analysis of virome data [66,67].

Here we present Hecatomb, a bioinformatics platform designed to address the above issues. Hecatomb supports the analysis of both long- and short-read technologies and various library types. The pipeline performs rigorous quality control followed by tiered alignment based taxonomic assignment using MMseqs2

[45]. Hecatomb also performs metagenomic assembly and annotation. Each step is managed using the Sankemake workflow manager with dependency management using Conda [68,69]. Hecatomb outputs several tables properly formatted for immediate use within popular data analysis and visualization tools enabling effective data interpretation for a variety of study designs.

IMPLEMENTATION

Hecatomb (RRID: SCR_025002) serves as an end-to-end pipeline by processing raw sequencing reads (single- or paired-end, long- or short-reads from Illumina, MGI, PacBio or Oxford Nanopore platforms) through four key modules (**Fig. 1**). In this way, it was designed to address many of the useability and functionality issues that are present in other software that we summarize in **Fig. S1**.

Module 1: Sequence Quality Control and Host Removal

Module 1 (Preprocessing) removes non-biological contaminants (i.e. primers, adapters) as well as common laboratory contaminants cataloged in NCBI's UniVec database [70]. The user can select to use fastp for contaminant removal from standard library preps, or BBTools for more complicated library preparation strategies such as the round A/B library which converts RNA into cDNA using a tagged-randomized primer and second-strand DNA synthesis, the resulting dsDNA is subjected to 10-40 rounds of PCR amplification. This process generates DNA/cDNA libraries for single- and double-stranded RNA and DNA viruses [71–73]. Low-quality sequences are also trimmed or removed prior to host removal.

Module 1 has an option to remove host-sequences (e.g. mouse, human) using Minimap2 [74]. This is optional as it may not be applicable to all samples (e.g. water, air, soil). Hecatomb comes packaged with several commonly used host-references genomes which have been masked of potential viral sequence. This masking is designed to minimize the inadvertent removal of viral sequences that may have similarities to the host sequence. Masked reference genomes were generated as follows: 1) all viral genomes from the National Center for Biotechnology Information (NCBI) viral assembly database [75] were downloaded and computationally "shredded" into short fragments with an average length of 85 bases sharing a 30-base overlap using shred.sh from the BBTools suite [73]. Shredded viral sequences were then mapped (minimum identity of 90% and at most 2 insertions or deletions) and masked from host-reference genomes using

BBmap requiring a [73]. Pre-computed masked reference genomes for the following host genomes are available in Hecatomb: human, mouse, rat, camel, *Caenorhabditis elegans*, dog, cow, macaque, mosquito, pig, rat and tick are available within Hecatomb. A command is provided to generate new masked genomes for additional hosts not included with Hecatomb.

Sequences free of contamination and host are clustered using the nucleotide version of Linclust packaged with MMSeqs2 [49,76]. Clustering reduces the number of sequences requiring taxonomic classification to a single, representative sequence thus greatly reducing the computational requirements for read based annotation in Module 2. Sequences are clustered requiring a minimum sequence identity of 97% and 80% alignment coverage of target sequence to the representative sequence (--min-seq-id 0.97 -c 0.8 --cov-mode 1). The size of each cluster per sample is maintained in the final output table (seqtable.fasta). This information serves as a “count table” for each sequence and values are provided as both raw and counts normalized to library size.

Module 2: Read-Based Annotation

Taxonomic and functional annotation is provided to reads in seqtable.fasta using a tiered approach **(Fig. 2A)**. All queries are carried out using MMseqs2 [49]. Queries against amino acid databases are performed using MMSeqs2 6-frame translation. Each read in seqtable.fasta is first queried against all viral (taxonomy id: 10239) amino acid sequences in UniProtKB clustered at 99% identity using Linclust (Viral AA DB) [76,77]. Sequences annotated as virus are subsequently queried against UniClust50 (Multi-kingdom AA DB) to remove false-positive annotations [78]. UniProt functional annotations are also applied when available.

Reads not identified as viral using queries against amino acid databases are queried against nucleotide databases **(Fig. 2A)**. Similar to the translated queries, each read is first queried against a virus-only reference sequence database (Virus NT DB). This database consists of all viral sequences in GenBank (taxonomy id: 10239) clustered at 100% identity using Linclust [49,76]. Sequences annotated as virus are subsequently queried against a customized nucleotide database (Polymicrobial NT DB) containing the Virus NT DB and representative RefSeq genomes from bacteria (1 per genus, n = 14,933), archaea (n =

511), fungi (n = 423), protozoa (n = 90) and plant (n = 145) genomes. These reference genomes represent a genomic ‘polymicrobial’ community and cover a large amount of microbial sequence space. This allows for the removal of false-positive annotations from the first query using a relatively small reference database.

Taxonomic annotations are augmented using a modified version of the 2b lowest common ancestor (2b-LCA) algorithm described in [79]. The 2b-LCA algorithm provides conservative taxonomic assignments towards lower-nodes of the tree when similarity is found across a heterogeneous collection of taxonomies. However, the LCA algorithm fails when crossing higher taxonomic ranks. For example, sequences with similarity to both bacterial and viral taxa have a LCA of “root” in the NCBI tree, while viruses from distinct viral domains (e.g., bacteriophage and vertebrate viruses) are assigned to “virus root”. Hecatomb will identify these instances and augment the annotations by reverting to the top-hit annotation. Each instance of this is flagged in the final output table so researchers can choose to include or exclude these from downstream analysis. This approach provides additional information about sequences with ambiguous taxonomic assignments instead of just leaving them as ‘root’ or ‘virus root’ and simply discarding them.

Sequence annotations from queries against both amino acid and nucleotide databases are combined into one table and assigned updated taxonomies using the most recent version of NCBI’s Taxonomy Database using TaxonKit [80,81] **(Fig. 2A)**. This taxonomy table contains full Linnaean taxonomic lineages (Kingdom, Phylum, Class, Order, Family, Genus and Species), alignment type used for annotation (translated (aa, amino acid database) or untranslated (nt, nucleotide database)) and LCA augmentation information. Due to hecatomb keeping track of individual sequence IDs throughout this process, it is then possible to combine these read-based taxonomic assignments to other data generated by Hecatomb or external data resources **(Fig. 2B)**. By default, Hecatomb will combine MMSeqs2 alignment information (e.g. target/query ID, e-value, % identity, alignment length, etc.), and count table information gathered during the clustering process. As an example of combining data to external resources, Hecatomb will provide Baltimore virus type information (both Baltimore class and Group). Baltimore is a classification system that places viruses into one of seven groups depending on a combination of their nucleic acid (DNA or RNA), strandedness (single-stranded or double-stranded), Sense, and method of replication. This could easily be extended to a variety of external data resources. Together these disparate data tables are collected into Hecatombs’

bigtable.tsv. As Hecatomb tracks sample identifiers, it is then possible to combine the bigtable with sample data. All of these data are formatted to make them easily importable into commonly used data analysis tools for statistical and graphical analysis.

Module 3: Assembly

By default, Hecatomb performs an assembly for individual samples using MEGAHIT for short-reads or Canu for long-reads **(Fig. 3)** [82,83]. Individual sample assemblies are then merged into a population assembly using Flye [84].

Per sample contig abundances are calculated by mapping individual sample reads to the population assembly using BBMap [85]. Read counts are reported normalized to library size and contig length using a variety of measures (reads per kilobase million (RPKM), fragments per kilobase million (FPKM) and sequences per million (SPM)). SPM is the same calculation as used for transcripts per kilobase million (TPM) except that the sequences are not assumed to be transcripts [86,87]. Additional contig properties (e.g. length, GC-content, coverage %) are combined with taxonomic assignments and sample abundance estimates into a final table (contig_count_table.tsv).

Options to skip the assembly step and to perform a cross-assembly are available for the user. Cross-assembly assembles all reads from all samples simultaneously (skipping the individual sample assemblies). This can result in better quality assemblies but is computationally expensive for larger datasets and may not be an option for many users.

Module 4: Contig-Based Annotation

Module 4 provides taxonomic annotations to contigs. Taxonomy is assigned to all contigs in the population assembly using MMseqs2 [49]. Each contig is queried against the same polymicrobial nucleotide database (Polymicrobial NT DB) used for read-based annotation (contigAnnotations.tsv). Additionally, information obtained from both the read-based annotation and assembly modules **(Fig. 1)** are combined. Read mapping information (start, stop, mapping quality, etc.) is maintained during the sample abundance estimation (mapping with BBMap) performed as part of Module 3 [85]. This mapping information is combined with

read-based annotations (bigtable.tsv) to generate a new table combining read based taxonomic information across each contig (contigSeqTable.tsv).

Installation and Dependency Management

Hecatomb is hosted on GitHub [88] and is available for installation from Bioconda [89] and the Python Package Index [90] easing installation for individual users with a single command. Hecatomb makes liberal use of Conda environments to ensure portability, ease of installation and proper versioning of software dependencies **(Fig. S2)**. All required and optional software dependencies are summarized in **Table S1** [49,71,74,81–85,91–95]. Hecatomb and Conda handle the installation of all dependencies. Conda environments for jobs are created automatically by Snakemake. The use of isolated Conda environments for Hecatomb minimizes package version conflicts, minimizes overhead when rebuilding environments for updated dependencies, and allows maintenance and customization of different Hecatomb versions.

While Hecatomb is a Snakemake pipeline, it uses the Snaketool command line interface to make running the pipeline as simple as possible [96]. Snaketool populates required file paths and configuration files, allowing Hecatomb to be configured and run with a simple command, and offers a convenient way to modify parameters and customize options.

High-Performance Computing Deployment

Hecatomb can be deployed on a high-performance computing (HPC) cluster and can utilize Snakemake profiles for cluster job schedulers (e.g. Slurm, SGE, etc.). Snakemake uses profiles to submit pipeline jobs to the job scheduler and monitor their progress. Profiles can be created manually, but Hecatomb has been designed for compatibility with the official Cookiecutter [97] profiles for Snakemake [98], and comes with a pre-installed Slurm example profile.

Customization

Hecatomb comes precompiled with many predefined settings regarding individual process options. These settings are highly customizable through the inclusion of a Snakemake YAML file. This file provides a single-source solution to user customization. Settings such as the quality threshold used for read trimming in

Module 1 or the length of contig to maintain in Module 3 can easily be adjusted per an individual user or project needs.

APPLICATION

Hecatomb accelerates profiling of viral metagenomes. We reanalyzed a previously published data set of 95 stool samples collected from simian immunodeficiency virus (SIV)-infected rhesus macaques (*Macaca mulatta*) (NCBI BioProject accession: PRJEB9503) [5]. Sequences were generated using the Illumina MiSeq 2×250 paired-end protocol using round A/B libraries (DNA and cDNA to enable detection of both RNA and DNA viruses) from virus-like particle preparations. These data contain sequences from a variety of RNA and DNA vertebrate viruses. The original study also identified a statistically significant difference in the abundance of several enteric viruses in SIV infected animals compared to uninfected animals. These data included samples ranging from 768,268 to 4,229,134 raw input reads with a duplication rate at 97% identity ranging from 3.98% to 56.06% (**Table S2**).

We first assessed Hecatombs' overall ability to detect diverse viral sequences. Hecatomb was executed using the round A/B preprocessing module, and default parameters. Hecatomb classified sequences into phylogenetically diverse viral groups **(Fig. 4A)**. Of the 2,394,740 reads annotated by hecatomb as viral, 1,989,352 (83%) were annotated using queries against protein (aa) databases and 405,388 (17%) annotated using queries against nucleotide databases (nt). Bacteriophage from the family *Microviridae* and the order *Caudovirales* were highly abundant. Sequences belonging to a diverse set of viruses associated with infection of plants and protists were also detected **(Fig. S3)**. Similar to the original study, Hecatomb identified a large number of sequences belonging to the *Picornaviridae* and *Adenoviridiae*. Sequences from these viral families were found to be more abundant in SIV-infected macaques when compared to uninfected animals in the original study (**Fig. 4C**).

Hecatomb collects and organizes alignment statistics (e.g. e-values, percent identity, alignment length, etc.) generated for each sequence annotation in Module 2. These data can be useful for assessing the prevalence and quality of viral annotations within a study. As an example, we examined the percent identity and alignment lengths of every read assigned taxonomy to the four families of viral enteropathogens

identified in the original study (*Circoviridae*, *Picornaviridae*, *Adenoviridae* and *Parvoviridae*) (**Fig. 4B**). Hecatomb annotated sequences for these four viral families using both translated queries to amino acid (aa) databases and untranslated queries to nucleotide (nt) databases. Quadrants were applied to visualize low- and high identity and short- and long-alignment lengths for every annotated sequence. Sequences in the upper two quadrants are highly similar to sequences in the reference databases over short (upper left, quartile 1 (Q1)) or long (upper right, Q2) alignment lengths, while sequences in the lower two quadrants have low similarity over short (lower left, Q3) or long (lower right, Q4) alignment lengths. For this analysis we arbitrarily selected 70% identity to represent the cut-off between low and high-identity for translated (aa database) and 90% identity for untranslated (nt database) alignments. These values are adjustable and could be customized for each study and viral family of interest. Using this framework, it is clear that a majority of sequences are high-identity (both short and long alignments) to sequences in both the aa and nt reference databases for the four families of enteropathogenic viruses.

In contrast, there were also a large number of sequences classified due to similarity to reference sequences from viruses of protists (**Fig. 4B**). Viruses in the family *Mimiviridae* infect *Acanthamoeba*, and those in the family *Phycodnaviridae* infect algae, are both dsDNA viruses with large genomes [99]. While it is conceivable that these viruses may exist in the stool samples of rhesus macaques via water or food, using the quadrant framework there is little evidence of high-identity alignments to any sequence in either the aa or nt databases (**Fig. 4B, Fig. S4**). Hecatomb does not automatically remove sequences from these families due to their presence in environmental datasets. There is evidence for short and long low identity alignments (quadrant 4) to both *Phycodnaviridae* and *Mimiviridae* reference sequences. Thus, these sequences should be analyzed using additional metrics (i.e. E-values, abundance across samples, etc.) to determine if these represent potentially novel viral sequences. This would not have been possible using stringent E-value filtering prior to data analysis.

Reevaluation of existing environmental metagenomic datasets. We assessed Hecatomb's ability to analyze non-human associated viromes by processing a previously studied coral reef dataset (NCBI BioProject accession: PRJNA595374, SRA study number SRP237459) [100,101]. The dataset consists of whole genome shotgun (WGS) metagenomic sequencing of both seawater and coral mucus from inner and

outer sections of a Bermuda reef system. The original studies only considered bacterial metagenome assembled genomes which makes it an excellent candidate for generating new biological insights by characterizing the viruses of this previously published dataset. The original study identified statistically significant differences in bacterial compositions between the coral mucus and seawater microbiomes and the coral mucus microbiomes from the inner and outer reefs. All analysis was performed using output from the contig annotations provided by Hecatombs module 3 and 4.

We first tested for differences in viral species alpha diversity using both Shannon diversity and richness comparing both inner and outer reef samples and coral mucus and reef water samples (**Fig. 5**). Both Shannon diversity and richness were significantly higher for inner reef samples compared to outer reef samples (**Fig. 5A**). Shannon diversity and richness were not significantly different between coral mucus and reef water. This result is in contrast with the bacterial diversity and richness metrics being similar across all samples as reported in the original study [100].

Next, we compared the viral compositions of these coral reef samples using beta diversity. Principle coordinate analysis (PCoA) of Bray-Curtis dissimilarity of viral genera, and Permutational multivariate analysis of variance (PERMANOVA) showed non-homogeneous distributions between inner and outer reef samples (p = 0.001) as well as between coral mucus and reef water samples (p = 0.015) (**Fig. 5B**). There is a strong separation of inner and outer reef samples along the x-axis and a weaker separation of reef water and coral mucus samples along the y-axis; this is the same trend observed in the original study for the bacterial compositions.

Linear regression (LR) to characterize the viral taxa that are driving the differences between inner and outer reefs, and between coral mucus and reef water samples. The R package microViz will calculate LR models at all taxa and taxon levels for the various sample groups. We calculated LR models to the genus level and generated tree plots for all taxa with prevalence greater than 10% of samples, coloured by the LM estimate coefficients, and weighted by prevalence between the inner and outer reef samples (**Fig. 5C**) and between the coral mucus and reef water samples (**Fig. 5D**). These analyses indicate that inner reef samples have significantly higher relative abundances of many viral taxa, including several *Caudoviricetes* taxa and especially the *Kyanoviridae* family which consist of various *Synechococcus* phages and Cyanophages (**Fig.**

5C). Conversely, far fewer viral taxa were more abundant in outer reef samples. Reef water samples contained elevated abundances of many viral taxa compared to coral mucus samples, with the main exception of the family of giant viruses *Mimiviridae* (**Fig. 5D**).

Accelerated discovery of novel viruses. Hecatomb retains the assembly graph as well as the assembly itself which downstream tools can utilize to resolve metagenome-assembled genomes. There was strong evidence for the presence of novel bacteriophage within the SIV-macaque dataset in the form of many high-quality but low identity alignments to known reference viruses (**Fig. S5**). We therefore processed the assembly graph with Phables and identified 127 probable complete phage genomes [61]. Phables bins fragmented assemblies into complete genomes. These genomes were assessed with CheckV which determined that 121 of them were high-quality complete phage genomes [102]. We assigned taxonomy using MMSeqs2 with the Hecatomb primary nucleotide databases (**Table S3**) [49]. Lastly, the genomes were annotated using Pharokka [103]. Of the 121 genomes, 98 were *Microviridae* (**Table S3**). Of these *Microviridae*, 96 exhibited the hallmark replication initiation protein followed by a major capsid protein, and a further 55 have the hallmark minor tail or pilot tail spike protein (**Fig. 6A**) while the other 41 contain hypothetical proteins where the tail spike protein would be. There were 10 *Caudoviricetes*, two *Cressdnaviricota* genomes, and eight that had hits to known phages with no taxonomic information. There were 13 cases where two genomes were resolved from the same assembly graph element and likely represent quasi-species that can occur for instance through recombination events [104,105]. We also processed the coral dataset using the same method. Synteny is also conserved in these larger phages, for instance, *Caudoviricetes* phage 1112C1 arranges its capsid and tail proteins together and exhibits the conserved layout described in [106] (**Fig. 6B**). In the coral dataset we identified three complete *Caudoviricetes* phage genomes (**Table S3**). The number of samples in this study was much lower and likely impacted the number of recovered viral genomes. The recovered viral genomes across both studies are novel with only 18 aligning to a known phage with an identity higher than 90% (**Table S3**). This demonstrates Hecatomb's utility for data mining published environmental metagenome projects to generate novel viral genomes.

Analysis of an *in silico* set of viral genomes. To determine the ability of hecatomb to accurately identify viral sequences in a mixed metagenome we generated a mock *in silico* data set. Canonical viral genomes from each genomic type (single and double-stranded DNA and RNA genomes and one retrovirus) were downloaded from NCBI (**Table 1**). For each genome, we simulated 1 x 10^4 250 base pair sequences using the Illumina error-model model available in InSilicoSeq [107]. Next, we generated 1 x 10^6 sequences from a collection of 1,520 cultivated human gut bacterial genomes [103] using the same Illumina error model. Simulated sequences from viruses and bacteria were combined to simulate a mixed virus-bacteria environment and processed through Hecatomb using the default settings. Hecatomb assigned the correct taxonomy to 99.64-99.65% of the simulated viral reads with a sensitivity ranging from 0.98-1 and a false-positive rate ranging from 8.8 x 10^{-4} to 1.35 x 10^{-3} (**Table 1**).

DISCUSSION

Virome sequencing is the premier approach to evaluate the viral content of both host-derived and environmental samples. It is useful for determining what types of viruses are present in individual samples and how virome compositions compare between sample groups. This information forms the foundations for answering a wide array of interesting biological questions. For example, virome composition has recently been analyzed as an indicator of the microbial impacts of climate change [108,109]. Virome sequencing was also critical to the discovery and characterization of SARS CoV-2 in 2019 [110]. Effective characterization of virome sequencing data requires rigorous and integrated software platforms to facilitate and accelerate virus discovery and virome compositional analysis. Given these tools researchers will be better prepared to assess how viruses are associated with some of the most important challenges to human life today.

All virome studies are dependent on effective computational tools to identify and classify viral reads or assembled contigs within a metagenome. Viral metagenomics is often dependent on identifying sequence similarity against reference sequence databases, either directly via homology-based searchers, or using machine learning techniques that have been trained on reference databases to identify features unique to viral sequences (reviewed in [111]). Homology-based searches can take a ‘brute force’ approach, wherein all unclassified sequences are queried against a comprehensive, multi-kingdom reference sequence

database (e.g. NCBI nt or nr). This approach relies on the search algorithm (e.g. BLAST, DIAMOND [48]) to pick the best or lowest-common ancestor of a group of hits to provide a final taxonomic assignment to an unknown query sequence. This approach is slow and requires significant computational resources, which is why Hecatomb takes an alternate approach. First, by capturing all 'potentially viral' sequences initially querying a small viral sequence database. The 'potentially viral' sequences typically represent a fraction of the full metagenomic data making subsequent computation more tractable. To confirm viral taxonomic assignment, potentially viral sequences are cross-checked against a curated small transkingdom reference database containing genomic representatives from all kingdoms of life. Hecatomb completes this iterative search approach using translated searches against amino acid databases as well as untranslated searches against nucleotide databases, combining the results of each to ensure detection of viral sequences is database independent. This iterative search strategy uses databases orders of magnitude smaller than comprehensive, multi-kingdom databases (such as NCBI's nt and nr) increasing computational efficiency without limiting viral detection.

Hecatomb's design philosophy recognizes that there are no 'perfect' databases or search algorithms. Both the brute force and iterative search approaches against comprehensive or curated databases will result in different rates of true/false positives/negatives. Instead, Hecatomb relies on providing a compiled and rich set of data for search result evaluation. We used this strategy to reassess the virome composition of SIV-infected and uninfected rhesus macaques [5]. The original study used an iterative approach, but relied on comprehensive, transkingdom databases (NCBI nt and nr), and identified associations between four families of animal viruses (*Circoviridae*, *Picornaviridae*, *Adenoviridae* and *Parvoviridae*) and SIV-infection. The new Hecatomb trans-kingdom database is 6 orders of magnitude smaller than GenBank nt (5.0×10^6 versus 1.3×10^{12}) which results in a significant reduction in computational time and resources. Hecatomb identified the same four viral families and their relationship to SIV mediated disease. Similar to our analysis of these samples using Hecatomb, the original study also classified a number of sequences to the *Mimiviridae* and *Phycodnaviridae*. Statistical comparison of these sequences between groups (e.g. SIV-infected vs. uninfected) did not reveal any significant associations thus they were not discussed further. However, new evaluation of results from Hecatomb indicates that there were likely false-positive classifications reported in the original analysis. Lastly, we identified 121 novel complete phage genomes in

this dataset. The majority of these genomes were *Microviridae*, which was the most abundant family by read abundance in the dataset. Recovering these genomes with a provisional taxonomic classification using Hecatomb's annotations is simple, fast, and scalable. This method is not a replacement for culturing and characterizing viruses in a lab environment. However, these *in silico* methods are essential when considering the scale of the viral dark matter problem. This reanalysis highlights how coordinated data such as alignment statistics and taxonomy can be powerful tools for virome evaluation and novel virus discovery.

Hecatomb was able to effectively evaluate the viromes of environmental (non-host associated) viromes. Leveraging the R packages phyloseq and microViz allowed us to quickly and easily complete the analysis in approximately 200 lines of code [66,67]. This analysis was primarily designed to identify compositional changes in viromes between reef types (inner or outer), and within coral mucosa and the surrounding water from a previously published metagenomic data set [100,101]. The original study identified elevated levels of *Pelagibacter*, *Synechococcus*, and unclassified Rickettsiales in inner reef samples compared to outer reef samples. Indeed, we found *Synechococcus* phages and *Cyanophages* were important for distinguishing the highly fluctuating inner reef system from the thermally stable outer reef. The authors showed that the bacterial microbiomes were unique for inner and outer reefs for both coral and mucus samples. We applied the same method on the viral compositions and confirmed that the viromes for these four sample types are also all unique.

Interestingly, viral species richness and diversity were significantly elevated in inner reef samples, whereas the original study found these to be similar in terms of bacterial composition. Viral activity is an important vehicle for nutrient cycling which was thought to be much higher in the inner reef based on the metabolic profiles of these samples [100]. There were many viral taxa that were more abundant in the inner reef samples, and few that were elevated in the outer reef. Similarly, many viruses were more abundant in reef water samples than coral mucosa, with the main exception of the giant viruses from the family *Mimiviridae*. It would be interesting to elucidate whether the coral mucus is impacting viral infectivity directly, or if the phages are switching to a lysogenic rather than lytic life cycle in this environment.

Corals occasionally shed their mucosa. Shedding occurs far more frequently in the inner reef due to stressors such as thermal fluctuation and sedimentation from surface runoff. The increased flux of nutrients

and microbes from corals to the surrounding reef water may be contributing to increased microbial and viral activity in inner reef samples compared to the outer reef. Different concentrations of microbes might also be having an impact. The outer reef systems are subject to upwelling, resulting in greater exchange of water with the open ocean which is probably flushing microbes and viruses from the environment. Unfortunately, it is not possible to infer microbial concentrations from WGS sequencing. The reanalysis of this coral dataset has generated many new hypotheses about viral host interactions within a coral reef system.

Potential implications

Virome analysis is complex and requires efficient computational tools to generate analyst friendly results. Hecatomb provides a comprehensive and computationally efficient solution for both read- and assembly-based viral annotation, virome analysis, and novel virus discovery. The pipeline is delivered with a convenient and easy-to-use front end, and is compatible with different sequencing technologies. Hecatomb's comprehensive collection of data throughout the pipeline's execution, in particular the collection of alignment statistics, empowers the identification and interrogation of viral taxonomic assignments. We demonstrate Hecatomb's utility for rapid processing and analysis of viral metagenomes with a well-studied validation gut viral metagenome dataset. We also demonstrate its utility for mining regular metagenome samples for virome analysis by analyzing an existing environmental dataset. Virome analysis is an evolving field requiring novel approaches for comprehensive characterization. For example, none of the described workflows account for spliced coding sequences (CDS), hampering the automation of viral genome annotation. The modularity of Hecatombs approach should enable easy integration of novel methods as they emerge.

Methods

All commands used for analyzing the Hecatomb annotations are available as a gist on GitHub [112].

Reevaluation of SIV dataset. We reanalyzed a previously published data set of 95 samples obtained from stool samples collected from SIV-infected rhesus macaques (*Macaca mulatta*) (NCBI BioProject accession: PRJEB9503) [5]. Sequence data were generated using the Illumina MiSeq 2×250 paired-end protocol on

libraries of total nucleic acid (DNA and cDNA to enable detection of both RNA and DNA viruses). Hecatomb was executed using the round A/B preprocessing module, and otherwise default parameters. Data were analyzed in R with Tidyverse [64]; commands are available in the above GitHub Gist.

Reevaluation of Coral microbiomes. We reanalyzed a coral reef dataset (NCBI BioProject accession: PRJNA595374, SRA study number SRP237459) [100,101] of whole genome shotgun (WGS) metagenomic sequencing (Illumina MiSeq, paired 2x250) of both seawater and coral mucus from inner and outer sections of a Bermuda reef system. Hecatomb was run with fast search parameters, cross assembly, and otherwise default parameters. Data were analyzed in R with PhyloSeq [66] and MicroViz [67]; commands are available in the above GitHub Gist.

Identification of phage genomes from Hecatomb assemblies. To identify complete phage genomes, the assembly graphs created by Hecatomb were processed with Phables [61]. The predicted phage genomes were assessed with CheckV [102]. High-quality and complete phage genomes were assigned provisional Taxonomic annotations using MMSeqs2 with the Hecatomb viral amino acid database (easy-taxonomy) and viral nucleotide database (easy-search plus TaxonKit). Lastly, the genomes were annotated with Pharokka [103].

Availability of source code and requirements

Project name: Hecatomb
Project home page: github.com/shandley/hecatomb [88]
Project documentation: hecatomb.readthedocs.io [113]
Operating system: Linux
Programming language: Python
Other requirements: Conda or pip
License: MIT
RRID: SCR_025002
Bio.tools ID: hecatomb
Restrictions to use by non-academics: None

Data availability

The reanalysis with Hecatomb utilized pre-existing datasets which are available under the NCBI BioProject accessions PRJEB9503 for the macaque SIV dataset [5] and PRJNA595374 (SRA: SRA study number SRP237459) for the coral reef dataset [100,101]. Accessions for novel phage genomes identified in this study are available in **Table S3**. An archival copy of the code and supporting data is also available via the GigaScience repository, GigaDB [114]. Hecatomb is registered on WorkflowHub [115].

List of abbreviations

AIDS: acquired immunodeficiency syndrome
ANOVA: analysis of variance
CPM: counts per million
FPKM: fragments per kilobase million
HPC: high-performance computing
ICTV: International Committee on Taxonomy of Viruses
LCA: lowest common ancestor
NCBI: National Center for Biotechnology Information
PERMANOVA: permutational analysis of variance
PCoA: principal coordinate analysis
RPKM: reads per kilobase million
SIMPER: similarity percentage
SIV: simian immunodeficiency virus
SPM: sequences per million
WGS: whole genome shotgun

Funding

Research reported in this publication was supported by grants from the NIH (RC2 DK116713 and U01 AI151810) awarded to RAE and SAH. MJR was supported by Flinders University under an Impact Seed Funding for Early Career Researchers grant.

Author's contributions

Conceptualization & Methodology: MJR, RAE, SAH; Software & Validation: MJR, SJB, KH-C, RAE, LACC, SAH; Formal Analysis: MJR, LFOL, RAE, EAD, LACC, SAH; Investigation: MJR, SAH; Visualization: MJR, KAM, LW, AP, SAH; Writing–Original Draft: MJR, RAE, SAH; Supervision, Project Administration & Funding Acquisition: RAE, SAH.

Acknowledgments

The authors thank Chandni Desai and Barry Hykes for their thoughtful commentary regarding the design philosophy of Hecatomb, and Sarah Giles, Susie Grigson, Bhavya Papudeshi, Vijini Mallawaarachchi, and Laura Inglis for feedback on the manuscript. The support provided by Flinders University for HPC research resources is acknowledged.

References

1. Hendrix RW, Smith MC, Burns RN, Ford ME, Hatfull GF. Evolutionary relationships among diverse bacteriophages and prophages: all the world's a phage. *Proc Natl Acad Sci U S A*. Elsevier; 96:2192–71999;

2. Koonin EV, Dolja VV, Krupovic M, Varsani A, Wolf YI, Yutin N, et al.. Global organization and proposed megataxonomy of the virus world. *Microbiol Mol Biol Rev*. American Society for Microbiology; 2020; doi: 10.1128/MMBR.00061-19.

3. Kim AH, Armah G, Dennis F, Wang L, Rodgers R, Droit L, et al.. Enteric virome negatively affects seroconversion following oral rotavirus vaccination in a longitudinally sampled cohort of Ghanaian infants. *Cell Host Microbe*. 30:110-123.e52022;

4. Maqsood R, Rodgers R, Rodriguez C, Handley SA, Ndao IM, Tarr PI, et al.. Discordant transmission of bacteria and viruses from mothers to babies at birth. *Microbiome*. 7:1562019;

5. Handley SA, Desai C, Zhao G, Droit L, Monaco CL, Schroeder AC, et al.. SIV Infection-Mediated Changes in Gastrointestinal Bacterial Microbiome and Virome Are Associated with Immunodeficiency and Prevented by Vaccination. *Cell Host Microbe*. 19:323–352016;

6. Norman JM, Handley SA, Baldridge MT, Droit L, Liu CY, Keller BC, et al.. Disease-specific alterations in the enteric virome in inflammatory bowel disease. *Cell*. 160:447–602015;

7. Neri U, Wolf YI, Roux S, Camargo AP, Lee B, Kazlauskas D, et al.. Expansion of the global RNA virome reveals diverse clades of bacteriophages. *Cell*. 185:4023-4037.e182022;

8. Zayed AA, Wainaina JM, Dominguez-Huerta G, Pelletier E, Guo J, Mohssen M, et al.. Cryptic and abundant marine viruses at the evolutionary origins of Earth's RNA virome. *Science*. 376:156–622022;

9. Williamson SJ, Allen LZ, Lorenzi HA, Fadrosh DW, Brami D, Thiagarajan M, et al.. Metagenomic exploration of viruses throughout the Indian Ocean. *PLoS One*. 7:e420472012;

10. Yang K, Wang X, Hou R, Lu C, Fan Z, Li J, et al.. Rhizosphere phage communities drive soil suppressiveness to bacterial wilt disease. *Microbiome*. 11:162023;

11. Pastrana DV, Peretti A, Welch NL, Borgogna C, Olivero C, Badolato R, et al.. Metagenomic Discovery of 83 New Human Papillomavirus Types in Patients with Immunodeficiency. *mSphere*. 2018; doi: 10.1128/mSphereDirect.00645-18.

12. Dutilh BE, Cassman N, McNair K, Sanchez SE, Silva GGZ, Boling L, et al.. A highly abundant bacteriophage discovered in the unknown sequences of human faecal metagenomes. *Nat Commun*. Nature Publishing Group; 5:1–112014;

13. Dai Z, Wang H, Wu H, Zhang Q, Ji L, Wang X, et al.. Parvovirus dark matter in the cloaca of wild birds. *Gigascience*. 2022; doi: 10.1093/gigascience/giad001.

14. Krishnamurthy SR, Wang D. Origins and challenges of viral dark matter. *Virus Res*. 239:136–422017;

15. Pargin E, Roach MJ, Skye A, Papudeshi B, Inglis LK, Mallawaarachchi V, et al.. The human gut virome: composition, colonization, interactions, and impacts on human health. *Front Microbiol*. 14:9631732023;

16. Rosseel T, Pardon B, De Clercq K, Ozhelvaci O, Van Borm S. False-positive results in metagenomic virus discovery: a strong case for follow-up diagnosis. *Transbound Emerg Dis*. 61:293–92014;

17. Skewes-Cox P, Sharpton TJ, Pollard KS, DeRisi JL. Profile hidden Markov models for the detection of viruses within metagenomic sequence data. *PLoS One*. 9:e1050672014;

18. Ponsero AJ, Hurwitz BL. The Promises and Pitfalls of Machine Learning for Detecting Viruses in Aquatic Metagenomes. *Front Microbiol*. 10:8062019;

19. Bai Z, Zhang Y-Z, Miyano S, Yamaguchi R, Fujimoto K, Uematsu S, et al.. Identification of bacteriophage genome sequences with representation learning. *Bioinformatics*. 38:4264–702022;

20. Pandolfo M, Telatin A, Lazzari G, Adriaenssens EM, Vitulo N. MetaPhage: an Automated Pipeline for Analyzing, Annotating, and Classifying Bacteriophages in Metagenomics Sequencing Data. *mSystems*. 7:e00741222022;

21. Miao Y, Liu F, Hou T, Liu Y. Virtifier: a deep learning-based identifier for viral sequences from metagenomes. *Bioinformatics*. 38:1216–222022;

22. Marquet M, Hölzer M, Pletz MW, Viehweger A, Makarewicz O, Ehricht R, et al.. What the Phage: a scalable workflow for the identification and analysis of phage sequences. *Gigascience*. 2022; doi: 10.1093/gigascience/giac110.

23. Guo J, Bolduc B, Zayed AA, Varsani A, Dominguez-Huerta G, Delmont TO, et al.. VirSorter2: a multi-classifier, expert-guided approach to detect diverse DNA and RNA viruses. *Microbiome*. 9:372021;

24. Tisza MJ, Pastrana DV, Welch NL, Stewart B, Peretti A, Starrett GJ, et al.. Discovery of several thousand highly diverse circular DNA viruses. *Elife*. elifesciences.org; 2020; doi: 10.7554/eLife.51971.

25. Ren J, Song K, Deng C, Ahlgren NA, Fuhrman JA, Li Y, et al.. Identifying viruses from metagenomic data using deep learning. *Quant Biol*. 8:64–772020;

26. Plyusnin I, Kant R, Jääskeläinen AJ, Sironen T, Holm L, Vapalahti O, et al.. Novel NGS pipeline for virus discovery from a wide spectrum of hosts and sample types. *Virus Evol*. 6:veaa0912020;

27. Auslander N, Gussow AB, Benler S, Wolf YI, Koonin EV. Seeker: alignment-free identification of bacteriophage genomes by deep learning. *Nucleic Acids Res*. academic.oup.com; 48:e1212020;

28. Kieft K, Zhou Z, Anantharaman K. VIBRANT: automated recovery, annotation and curation of microbial viruses, and evaluation of viral community function from genomic sequences. *Microbiome*. 8:902020;

29. Deaton J, Yu FB, Quake SR. Mini-Metagenomics and Nucleotide Composition Aid the Identification and Host Association of Novel Bacteriophage Sequences. *Adv Biosyst*. 3:e19001082019;

30. Fang Z, Tan J, Wu S, Li M, Xu C, Xie Z, et al.. PPR-Meta: a tool for identifying phages and plasmids from metagenomic fragments using deep learning. *Gigascience*. 2019; doi: 10.1093/gigascience/giz066.

31. Bin Jang H, Bolduc B, Zablocki O, Kuhn JH, Roux S, Adriaenssens EM, et al.. Taxonomic assignment of uncultivated prokaryotic virus genomes is enabled by gene-sharing networks. *Nat Biotechnol*. 37:632–92019;

32. Liu Q, Liu F, He J, Zhou M, Hou T, Liu Y. VFM: Identification of bacteriophages from metagenomic bins and contigs based on features related to gene and genome composition. *IEEE Access*. Institute of Electrical and Electronics Engineers (IEEE); 7:177529–382019;

33. Tampuu A, Bzhalava Z, Dillner J, Vicente R. ViraMiner: Deep learning on raw DNA sequences for identifying viral genomes in human samples. *PLoS One*. 14:e02222712019;

34. Garretto A, Hatzopoulos T, Putonti C. virMine: automated detection of viral sequences from complex metagenomic samples. *PeerJ*. 7:e66952019;

35. Zheng T, Li J, Ni Y, Kang K, Misiakou M-A, Imamovic L, et al.. Mining, analyzing, and integrating viral signals from metagenomic data. *Microbiome*. 7:422019;

36. Tithi SS, Aylward FO, Jensen RV, Zhang L. FastViromeExplorer: a pipeline for virus and phage identification and abundance profiling in metagenomics data. *PeerJ*. 6:e42272018;

37. Abdelkareem AO, Khalil MI, Elaraby M, Abbas H, Elbehery AHA. VirNet: Deep attention model for viral reads identification. *2018 13th International Conference on Computer Engineering and Systems (ICCES)*. p. 623–6.

38. Ren J, Ahlgren NA, Lu YY, Fuhrman JA, Sun F. VirFinder: a novel k-mer based tool for identifying viral sequences from assembled metagenomic data. *Microbiome*. 5:692017;

39. Laffy PW, Wood-Charlson EM, Turaev D, Weynberg KD, Botté ES, van Oppen MJH, et al.. HoloVir: A Workflow for Investigating the Diversity and Function of Viruses in Invertebrate Holobionts. *Front Microbiol*. 7:8222016;

40. Jurtz VI, Villarroel J, Lund O, Voldby Larsen M, Nielsen M. MetaPhinder-Identifying Bacteriophage Sequences in Metagenomic Data Sets. *PLoS One*. 11:e01631112016;

41. Li Y, Wang H, Nie K, Zhang C, Zhang Y, Wang J, et al.. VIP: an integrated pipeline for metagenomics of virus identification and discovery. *Sci Rep*. 6:237742016;

42. Roux S, Enault F, Hurwitz BL, Sullivan MB. VirSorter: mining viral signal from microbial genomic data. *PeerJ*. 3:e9852015;

43. Tisza MJ, Belford AK, Domínguez-Huerta G, Bolduc B, Buck CB. Cenote-Taker 2 democratizes virus discovery and sequence annotation. *Virus Evol*. 7:veaa1002021;

44. Camargo AP, Roux S, Schulz F, Babinski M, Xu Y, Hu B, et al.. Identification of mobile genetic elements with geNomad. *Nat Biotechnol*. 2023; doi: 10.1038/s41587-023-01953-y.

45. Zhao G, Wu G, Lim ES, Droit L, Krishnamurthy S, Barouch DH, et al.. VirusSeeker, a computational pipeline for virus discovery and virome composition analysis. *Virology*. 503:21–302017;

46. Kalantar KL, Carvalho T, de Bourcy CFA, Dimitrov B, Dingle G, Egger R, et al.. IDseq-An open source cloud-based pipeline and analysis service for metagenomic pathogen detection and monitoring. *Gigascience*. 2020; doi: 10.1093/gigascience/giaa111.

47. Altschul SF, Gish W, Miller W, Myers EW, Lipman DJ. Basic local alignment search tool. *J Mol Biol*. 215:403–101990;

48. Buchfink B, Xie C, Huson DH. Fast and sensitive protein alignment using DIAMOND. *Nat Methods*. 12:59–602015;

49. Steinegger M, Söding J. MMseqs2 enables sensitive protein sequence searching for the analysis of massive data sets. *Nat Biotechnol*. nature.com; 35:1026–82017;

50. Shen W, Xiang H, Huang T, Tang H, Peng M, Cai D, et al.. KMCP: accurate metagenomic profiling of both prokaryotic and viral populations by pseudo-mapping. *Bioinformatics*. 2023; doi: 10.1093/bioinformatics/btac845.

51. Wood DE, Lu J, Langmead B. Improved metagenomic analysis with Kraken 2. *Genome Biol*. 20:2572019;

52. Breitwieser FP, Baker DN, Salzberg SL. KrakenUniq: confident and fast metagenomics classification using unique k-mer counts. *Genome Biol*. 19:1982018;

53. Kim D, Song L, Breitwieser FP, Salzberg SL. Centrifuge: rapid and sensitive classification of metagenomic sequences. *Genome Res*. 26:1721–92016;

54. Monaco CL, Gootenberg DB, Zhao G, Handley SA, Ghebremichael MS, Lim ES, et al.. Altered Virome and Bacterial Microbiome in Human Immunodeficiency Virus-Associated Acquired Immunodeficiency Syndrome. *Cell Host Microbe*. 19:311–222016;

55. Li D, Luo R, Liu C-M, Leung C-M, Ting H-F, Sadakane K, et al.. MEGAHIT v1.0: A fast and scalable metagenome assembler driven by advanced methodologies and community practices. *Methods*. 102:3–112016;

56. Roux S, Emerson JB, Eloe-Fadrosh EA, Sullivan MB. Benchmarking viromics: an evaluation of metagenome-enabled estimates of viral community composition and diversity. *PeerJ*. 5:e38172017;

57. Nurk S, Meleshko D, Korobeynikov A, Pevzner PA. metaSPAdes: a new versatile metagenomic assembler. *Genome Res*. 27:824–342017;

58. Peng Y, Leung HCM, Yiu SM, Chin FYL. IDBA-UD: a de novo assembler for single-cell and metagenomic sequencing data with highly uneven depth. *Bioinformatics*. 28:1420–82012;

59. Antipov D, Raiko M, Lapidus A, Pevzner PA. Metaviral SPAdes: assembly of viruses from metagenomic data. *Bioinformatics*. 36:4126–92020;

60. Antipov D, Rayko M, Kolmogorov M, Pevzner PA. viralFlye: assembling viruses and identifying their hosts from long-read metagenomics data. *Genome Biol*. 23:572022;

61. Mallawaarachchi V, Roach MJ, Papudeshi B, Giles SK, Grigson SR, Decewicz P, et al.. Phables: from fragmented assemblies to high-quality bacteriophage genomes. bioRxiv.

62. Ho SFS, Wheeler NE, Millard AD, van Schaik W. Gauge your phage: benchmarking of bacteriophage identification tools in metagenomic sequencing data. *Microbiome*. 11:842023;

63. R Core Team. R: A Language and Environment for Statistical Computing. Vienna, Austria;

64. Wickham H, Averick M, Bryan J, Chang W, McGowan L, François R, et al.. Welcome to the tidyverse. *J Open Source Softw*. The Open Journal; 4:16862019;

65. Wickham H. Ggplot2: Elegant graphics for data analysis. 2nd ed. Cham, Switzerland: Springer International Publishing;

66. McMurdie PJ, Holmes S. phyloseq: an R package for reproducible interactive analysis and graphics of microbiome census data. *PLoS One*. 8:e612172013;

67. Barnett D, Arts I, Penders J. microViz: an R package for microbiome data visualization and statistics. *J Open Source Softw*. The Open Journal; 6:32012021;

68. Mölder F, Jablonski KP, Letcher B, Hall MB, Tomkins-Tinch CH, Sochat V, et al.. Sustainable data analysis with Snakemake. *F1000Res*. 10:332021;

69. . Anaconda Software Distribution. Anaconda Inc.;

70. Cochrane GR, Galperin MY. The 2010 Nucleic Acids Research Database Issue and online Database Collection: a community of data resources. *Nucleic Acids Res*. 38:D1-42010;

71. Chen S, Zhou Y, Chen Y, Gu J. fastp: an ultra-fast all-in-one FASTQ preprocessor. *Bioinformatics*. academic.oup.com; 34:i884–902018;

72. Finkbeiner SR, Holtz LR, Jiang Y, Rajendran P, Franz CJ, Zhao G, et al.. Human stool contains a previously unrecognized diversity of novel astroviruses. *Virol J*. Springer; 6:1612009;

73. Bushnell B. BBTools.

74. Li H. Minimap2: pairwise alignment for nucleotide sequences. *Bioinformatics*. academic.oup.com; 34:3094–1002018;

75. NCBI: Viral assembly database. ncbi.nlm.nih.gov/assembly/?term=viruses (2024). Accessed 2023.

76. Steinegger M, Söding J. Clustering huge protein sequence sets in linear time. *Nat Commun*. 9:25422018;

77. UniProt Consortium. UniProt: the universal protein knowledgebase in 2021. *Nucleic Acids Res*. 49:D480–92021;

78. Mirdita M, von den Driesch L, Galiez C, Martin MJ, Söding J, Steinegger M. Uniclust databases of clustered and deeply annotated protein sequences and alignments. *Nucleic Acids Res*. 45:D170–62017;

79. Hingamp P, Grimsley N, Acinas SG, Clerissi C, Subirana L, Poulain J, et al.. Exploring nucleo-cytoplasmic large DNA viruses in Tara Oceans microbial metagenomes. *ISME J*. 7:1678–952013;

80. Schoch CL, Ciufo S, Domrachev M, Hotton CL, Kannan S, Khovanskaya R, et al.. NCBI Taxonomy: a comprehensive update on curation, resources and tools. *Database* . 2020; doi: 10.1093/database/baaa062.

81. Shen W, Ren H. TaxonKit: A practical and efficient NCBI taxonomy toolkit. *J Genet Genomics*. Elsevier; 48:844–502021;

82. Li D, Liu C-M, Luo R, Sadakane K, Lam T-W. MEGAHIT: an ultra-fast single-node solution for large and complex metagenomics assembly via succinct de Bruijn graph. *Bioinformatics*. academic.oup.com; 31:1674–62015;

83. Koren S, Walenz BP, Berlin K, Miller JR, Bergman NH, Phillippy AM. Canu: scalable and accurate long-read assembly via adaptive k-mer weighting and repeat separation. *Genome Res*. genome.cshlp.org; 27:722–362017;

84. Kolmogorov M, Yuan J, Lin Y, Pevzner PA. Assembly of long, error-prone reads using repeat graphs. *Nat Biotechnol*. nature.com; 37:540–62019;

85. Bushnell B. BBMap: A fast, accurate, splice-aware aligner. Lawrence Berkeley National Lab. (LBNL), Berkeley, CA (United States); 2014 Mar. Report No.: LBNL-7065E.

86. Li B, Dewey CN. RSEM: accurate transcript quantification from RNA-Seq data with or without a reference genome. *BMC Bioinformatics*. 12:3232011;

87. Wagner GP, Kin K, Lynch VJ. Measurement of mRNA abundance using RNA-seq data: RPKM measure is inconsistent among samples. *Theory Biosci*. 131:281–52012;

88. Roach MJ, Beecroft SJ, Mihindukulasuriya KA, Wang L, Paredes A, Cárdenas LAC, et al.: Hecatomb @ GitHub. https://github.com/shandley/hecatomb (2020). Accessed 2024.

89. Roach M: Hecatomb @ Bioconda. https://anaconda.org/bioconda/hecatomb (2021). Accessed 2024.

90. Roach M: Hecatomb @ PyPI. https://pypi.org/project/hecatomb/ (2023). Accessed 2024.

91. Roach MJ, Pierce-Ward NT, Suchecki R, Mallawaarachchi V, Papudeshi B, Handley SA, et al.. Ten simple rules and a template for creating workflows-as-applications. OSF Preprints.

92. Köster J, Rahmann S. Snakemake—a scalable bioinformatics workflow engine. *Bioinformatics*. academic.oup.com; 2012;

93. Li H, Handsaker B, Wysoker A, Fennell T, Ruan J, Homer N, et al.. The Sequence Alignment/Map format and SAMtools. *Bioinformatics*. academic.oup.com; 25:2078–92009;

94. Shen W, Le S, Li Y, Hu F. SeqKit: A Cross-Platform and Ultrafast Toolkit for FASTA/Q File Manipulation. *PLoS One*. journals.plos.org; 11:e01639622016;

95. Roach MJ, Hart BJ, Beecroft SJ, Papudeshi B, Inglis LK, Grigson SR, et al.. Koverage: Read-coverage analysis for massive (meta)genomics datasets. *J Open Source Softw*. The Open Journal; 9:62352024;

96. Roach MJ, Tessa Pierce-Ward N, Suchecki R, Mallawaarachchi V, Papudeshi B, Handley SA, et al.. Ten simple rules and a template for creating workflows-as-applications. PLOS Computational Biology.

97. Greenfeld AR, Greenfeld DR, Pierzina R, Joseph M, Moore P, Shpak A, et al.: Cookiecutter: A command-line utility that creates projects from cookiecutter project templates. https://github.com/cookiecutter/cookiecutter (2013). Accessed 2024.

98. Köster J: Snakemake profiles. https://github.com/Snakemake-Profiles/doc (2017). Accessed 2024.

99. Sun T-W, Yang C-L, Kao T-T, Wang T-H, Lai M-W, Ku C. Host Range and Coding Potential of Eukaryotic Giant Viruses. *Viruses*. 2020; doi: 10.3390/v12111337.

100. Lima LFO, Alker A, Papudeshi B, Morris M, Edwards R, de Putron S, et al.. Coral and Seawater Metagenomes Reveal Key Microbial Functions to Coral Health and Ecosystem Functioning Shaped at Reef Scale. 2021;

101. Lima LFO, Weissman M, Reed M, Papudeshi B, Alker AT, Morris MM, et al.. Modeling of the Coral Microbiome: the Influence of Temperature and Microbial Network. *MBio*. 2020; doi: 10.1128/mBio.02691-19.

102. Nayfach S, Camargo AP, Schulz F, Eloe-Fadrosh E, Roux S, Kyrpides NC. CheckV assesses the quality and completeness of metagenome-assembled viral genomes. *Nat Biotechnol*. 39:578–852021;

103. Bouras G, Nepal R, Houtak G, Psaltis AJ, Wormald P-J, Vreugde S. Pharokka: a fast scalable bacteriophage annotation tool. *Bioinformatics*. 2023; doi: 10.1093/bioinformatics/btac776.

104. Routh A, Ordoukhanian P, Johnson JE. Nucleotide-resolution profiling of RNA recombination in the encapsidated genome of a eukaryotic RNA virus by next-generation sequencing. *J Mol Biol*. 424:257–692012;

105. Silva JM, Pratas D, Caetano T, Matos S. The complexity landscape of viral genomes. *Gigascience*. 2022; doi: 10.1093/gigascience/giac079.

106. Kang HS, McNair K, Cuevas DA, Bailey BA, Segall AM, Edwards RA. Prophage genomics reveals patterns in phage genome organization and replication. bioRxiv.

107. Gourlé H, Karlsson-Lindsjö O, Hayer J, Bongcam-Rudloff E. Simulating Illumina metagenomic data with InSilicoSeq. *Bioinformatics*. 35:521–22019;

108. Zhong Z-P, Vik D, Rapp J, Zablocki O, Maughan H, Temperton B, et al.. Lower viral evolutionary pressure under stable versus fluctuating conditions in subzero Arctic brines. 2023;

109. Han L-L, Yu D-T, Bi L, Du S, Silveira C, Cobián Güemes AG, et al.. Distribution of soil viruses across China and their potential role in phosphorous metabolism. *Environ Microbiome*. 17:62022;

110. Zhu N, Zhang D, Wang W, Li X, Yang B, Song J, et al.. A Novel Coronavirus from Patients with Pneumonia in China, 2019. *N Engl J Med*. 382:727–332020;

111. Kieft K, Anantharaman K. Virus genomics: what is being overlooked? *Curr Opin Virol*. 53:1012002022;

112. Roach M, Handley S: Reanalysis of Hecatomb datasets. https://gist.github.com/beardymcjohnface/3d3245b2bf6d9544c524f412037d5065 (2022). Accessed 2024.

113. Roach M, Handley S, Henry-Cocks K: Hecatomb @ ReadTheDocs. https://hecatomb.readthedocs.io/en/latest/ (2022). Accessed 2024.

114. Michael RJ, Sarah BJ, Kathie MA, Leran W, Anne P, Luis CCA, et al.. Supporting data for “Hecatomb: An integrated software platform for viral metagenomics.” GigaScience Database. 2024 http://dx.doi.org/10.5524/102506

115. Roach M: Hecatomb @ Workflow Hub. https://workflowhub.eu/workflows/235 (2022). Accessed 2024.

Figure Legends

Figure 1: Hecatomb pipeline and implementation

The Hecatomb pipeline is divided into four modules. (1) Reads for each sample undergo preprocessing and clustering (*orange*); (2) clustered reads undergo annotation using viral and multi-kingdom protein databases and clustered reads not annotated by the protein search are annotated using viral and multi-kingdom nucleotide databases (*blue*); (3) quality trimmed reads for each sample undergo assembly and assemblies for each sample are coalesced into a single assembly (*green*); (4) read-based annotations are combined with the assembly to provide contig annotations (*pink*). The assembly stages–*green* and *pink*–can optionally be skipped.

Figure 2: Read-based annotation

(a) Tiered annotation strategy. All alignments are completed using MMSeqs2. **(1)** High-quality representative sequences are queried against a viral amino acid (aa, *green*) sequence database. **(2)** Potentially viral sequences are subjected to a secondary, confirmatory query against a multi-kingdom amino acid sequence database. **(3)** Representative sequences that do not match a known viral amino acid sequence are subjected to an untranslated query to a viral nucleic acid sequence database (nt, *purple*) **(4)** followed by a secondary, confirmatory query against a polymicrobial nucleotide database **(5)**. Sequences that have been classified as either viral (*blue*) or nonviral (*pink*) in either the translated (aa database) or untranslated (nt database) queries are combined into a final taxonomy table. **(b)** Read annotation data structure. **(1)** Read Annotations are generated using the clustered sequences (seqtable.fasta). **(2)** The clustered sequence IDs are unpacked to yield the sample ID, the number of reads that sequence represents, and the percent of host-removed reads that sequence represents. **(3)** The alignment metrics from the annotation module are joined into the read annotations using the sequence ID as the primary key. **(4)** Taxonomic annotations are calculated and joined into the read annotations again using the sequence ID. **(5)** ICTV viral classifications are joined into the read annotations by the Taxonomic Family annotation. **(6)** Sample metadata can be joined into the read annotation table using the sample ID as the primary key. **(7)** The read annotation table with sample metadata can be quickly and easily analyzed.

Figure 3: Viral metagenome Merged-Assembly

(1) High-quality kmer-normalized sequences from individual samples are assembled using either MEGAHIT or Canu. **(2)** The sequences for each sample are mapped to their respective assemblies. **(3)** The unmapped reads from all samples are pooled together. **(4)** The pooled unmapped reads are assembled using either MEGAHIT or Canu. **(5)** The contigs from all sample assemblies and the unmapped reads assembly are combined together. **(6)** Overlapping contigs are joined together using Flye using the subassemblies algorithm.

Figure 4: Reanalysis of rhesus macaque stool viromes

(A) Abundance of reads classified by viral Phylum (color) and Type (shape) from 2,394,740 input sequences (all annotated sequences from the entire study). Phyla represented by fewer than 1,000 reads were excluded. **(B)** Percent identity and alignment lengths of all sequences classified for the 4 animal viruses identified in the previous study and two viruses of protists. Horizontal (70% identity) and vertical (150 base alignment length) dashed lines indicate a user-defined quadrant space. Each point represents an individual sequence colored by classification method (aa = translated search to an amino acid database, nt = classified via an untranslated search to a nucleotide database). Panels A and B represent data obtained from all 95 samples in the study. **(C)** Comparison of the number of sequences in SIV-infected and uninfected samples 5-weeks post-infection with SIV and at the time of necropsy. Significance determined by the Wilcoxon signed-rank test. * = $P \leq 0.05$, ** $P \leq 0.01$, *** $P \leq 0.001$, **** $P \leq 0.0001$. CPM = Counts per million.

Figure 5: Reanalysis of Coral Reef Metagenomes

(A) Viral species richness and Shannon diversity boxplots of inner and outer reef samples, colored by sample type. Significance (p-value < 0.05) is indicated. **(B)** Principal coordinates analysis (PCoA) of viral genera abundance. Inner and outer reef water samples are colored light blue and dark blue respectively. Inner and outer coral mucus samples are colored light and dark green respectively. Permutational multivariate analysis of variance (PERMANOVA) identified non-homogeneous distributions of inner versus outer reef samples (p = 0.001) and coral mucus versus reef water samples (p = 0.015). Ellipses for sample

groups are drawn at 85% confidence levels for multivariate t-distribution. **(C)** Dendrogram of most prevalent viral taxa (> 10% of samples). Linear regression (LR) models were generated for all taxa for the outer reef samples compared with the inner reef samples. Nodes are weighted by prevalence, and nodes and edges are colored by the LR model estimate coefficients. Significant LR models ($p < 0.05$) are indicated, and taxa with absolute coefficients greater than three are labeled. Nodes colored red are elevated in outer reef samples whereas nodes colored blue are elevated in inner reef samples. **(D)** Same as for Fig. 5.C except LR models are calculated for coral mucus samples compared with reef water samples. Nodes colored red are elevated in outer reef samples whereas nodes colored blue are elevated in inner reef samples.

Figure 6. Circos plots of complete bacteriophage genomes.

Circos plots were generated for all novel bacteriophage genomes using Phrokka's pharokka_plotter.py script (circos plots available at 10.5281/zenodo.6388251). **(A)** Circos plot for uncultured *Microviridae* phage 977C1. **(B)** Circos plot for uncultured *Caudoviricetes* phage 1112C1.

Figure S1. Features and useability of popular viral metagenomics software as of March 2023.

'Packaged install' indicates that the software is installable from any package manager or Apptainer. Software is considered 'recently updated' if it has been updated within the last 12 months. Software has been selected from a community-driven compilation of viral bioinformatics tools (an archived version is available at 10.5281/zenodo.6388251).

Figure S2: Implementation of Hecatomb using Snaketool, Snakemake, and Conda

Hecatomb takes in command line arguments, data, configuration parameters and outputs both results for analysis and run information. Hecatomb interacts with the job scheduler in high-performance computing (HPC) environments. Hecatomb distributes individual tasks to the job queue. Command-line arguments, *grey*; files, *yellow*; Conda environments, *blue*; scripts/programs, *green*; workload manager, *pink*.

Figure S3: Taxonomic subsets of virus types

Viral families present in the 95-sample SIV reanalysis study **(A)** Plant viruses, and **(C)** Protist viruses

Figure S4: Sequence per Quadrant Evaluation

Percentage of reads per quadrant in Figure 5. **(A)** translated (aa reference database) and **(B)** untranslated (nt reference database)

Figure S5: Alignments of bacteriophage sequences from rhesus macaque stool viromes

Alignment lengths and percent identities are shown in separate plots for the bacteriophage orders *Caudovirales*, *Levivirales*, *Petitvirales*, and *Tubulavirales*. Alignments are colored by viral family.

 Click here to access/download;Table;table_01.pdf

Table 1. Analysis of an *in silico* generated mock viral-bacterial community

Virus	NCBI Accession	Genome Type	Genome Length (bp)	True Positive	False Positive	Sensitivity	False Positive Rate
**Human gammaherpesvirus 4**	NC_007605.1	dsDNA	171823	9910	1345	0.99	1.35×10^{-3}
**Human Parvovirus B19**	NC_000883.2	ssDNA	5596	9964	873	1.00	8.8×10^{-4}
**Rotavirus A**	NC_011507:0-10	dsRNA	18562	9856	134	0.99	1.35×10^{-4}
**Rabies Virus**	NC_001542.1	ssRNA	11932	9932	654	0.99	6.6×10^{-4}
**Human Immunodeficiency Virus 1**	NC_001802.1	retrovirus	9181	9876	564	0.99	5.69×10^{-4}
**Crassphage**	NC_055760	dsDNA	94878	9765	4578	0.98	4.58×10^{-3}

Figure_01

Click here to access/download;Figure;Fig1.pdf

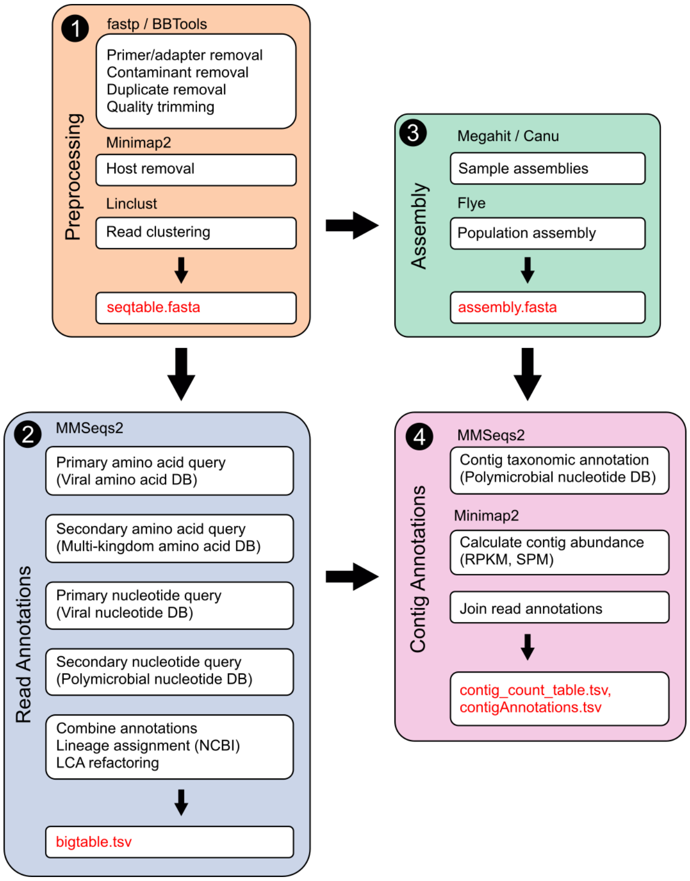

Figure_02 Click here to access/download;Figure;Fig2.pdf

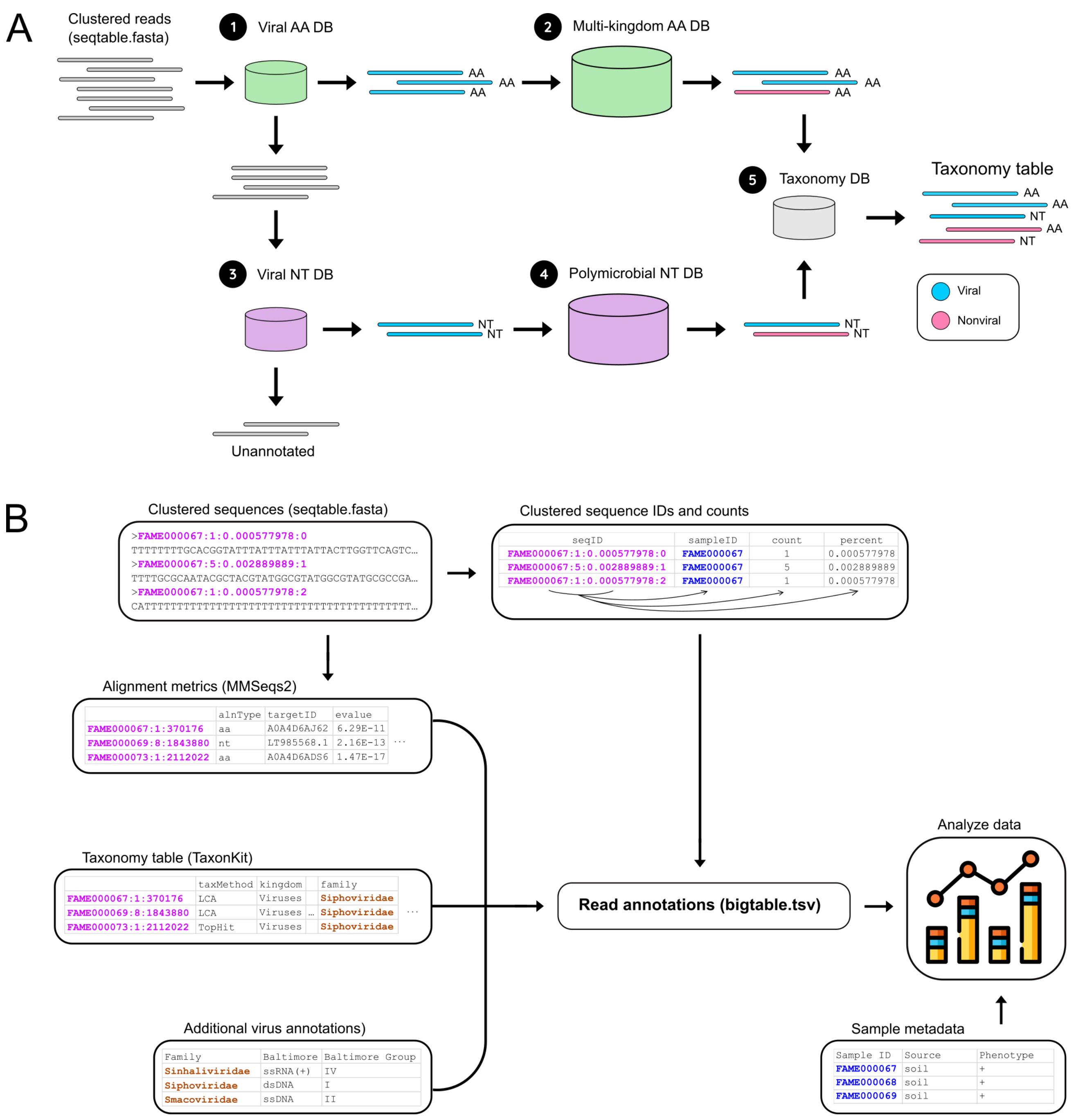

Click here to access/download;Figure;Fig3.pdf

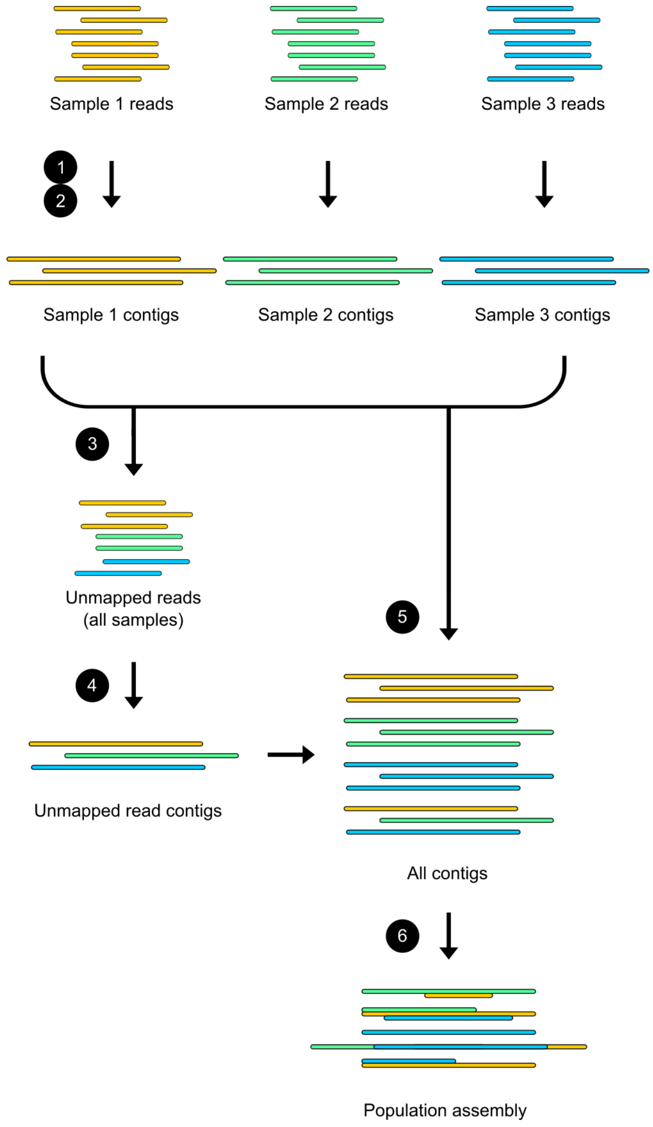

Figure_04

Click here to access/download;Figure;Fig4_updated.pdf

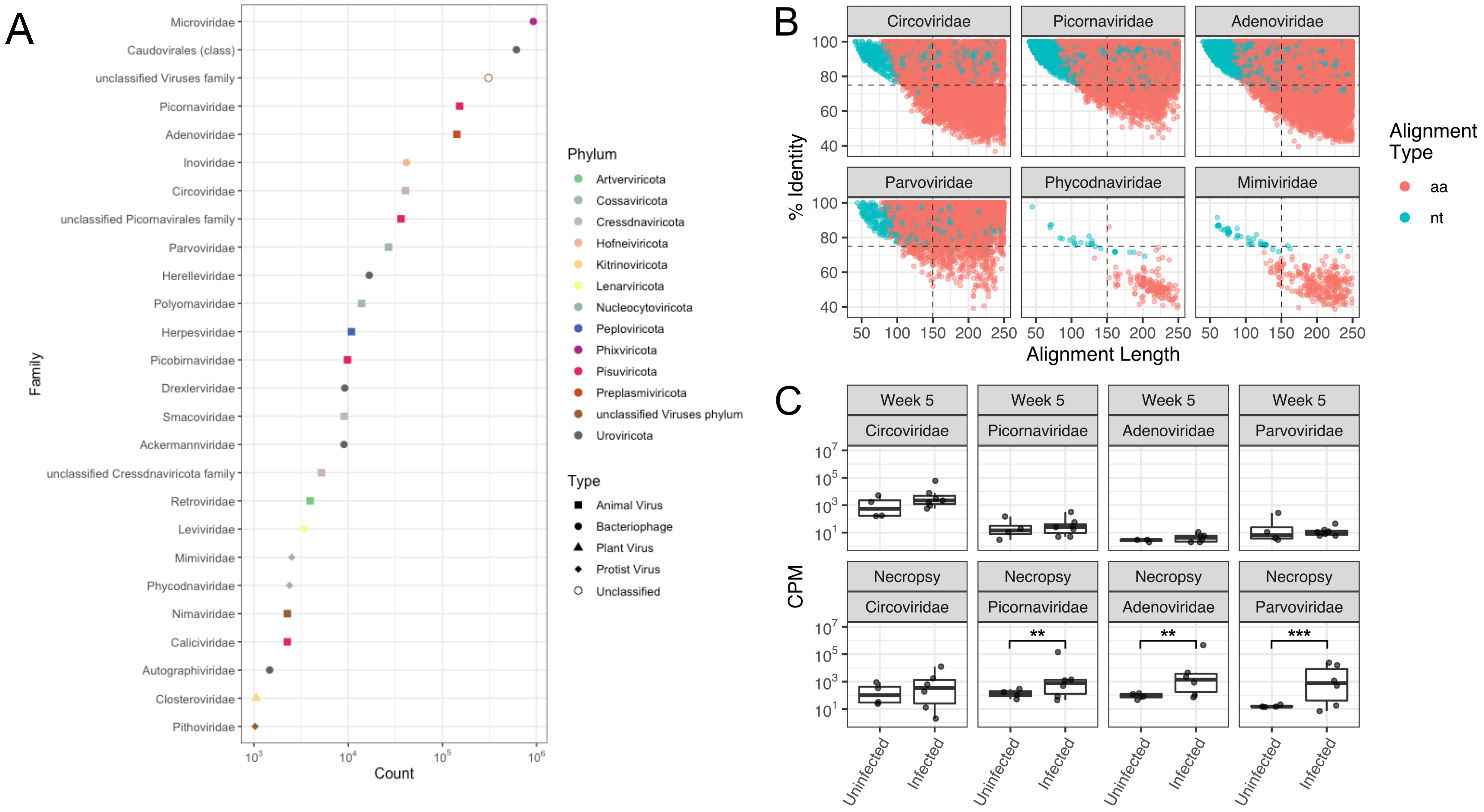

Figure_05

Click here to access/download;Figure;Fig5.pdf

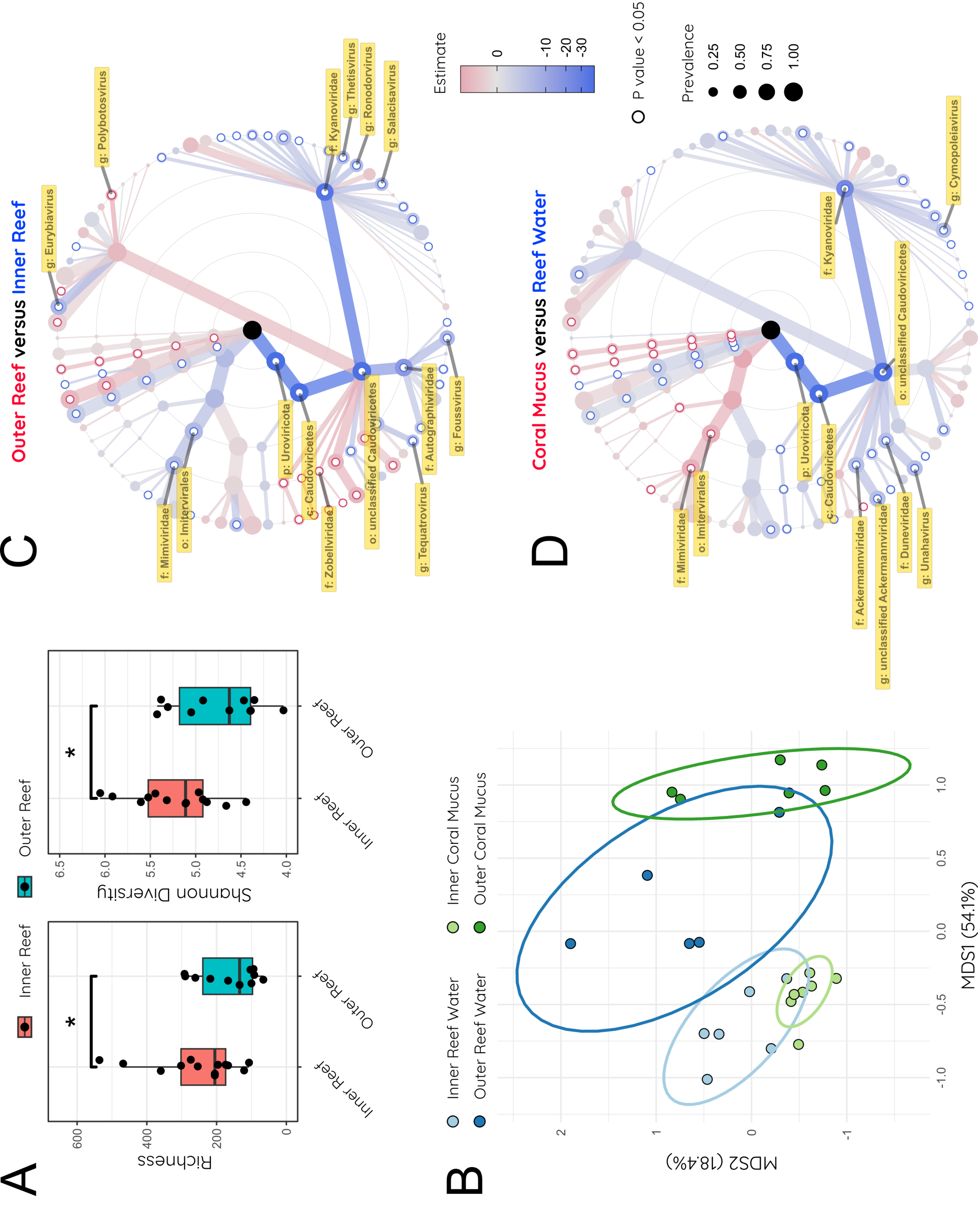

Figure_06

Click here to access/download;Figure;Fig6_revised.png

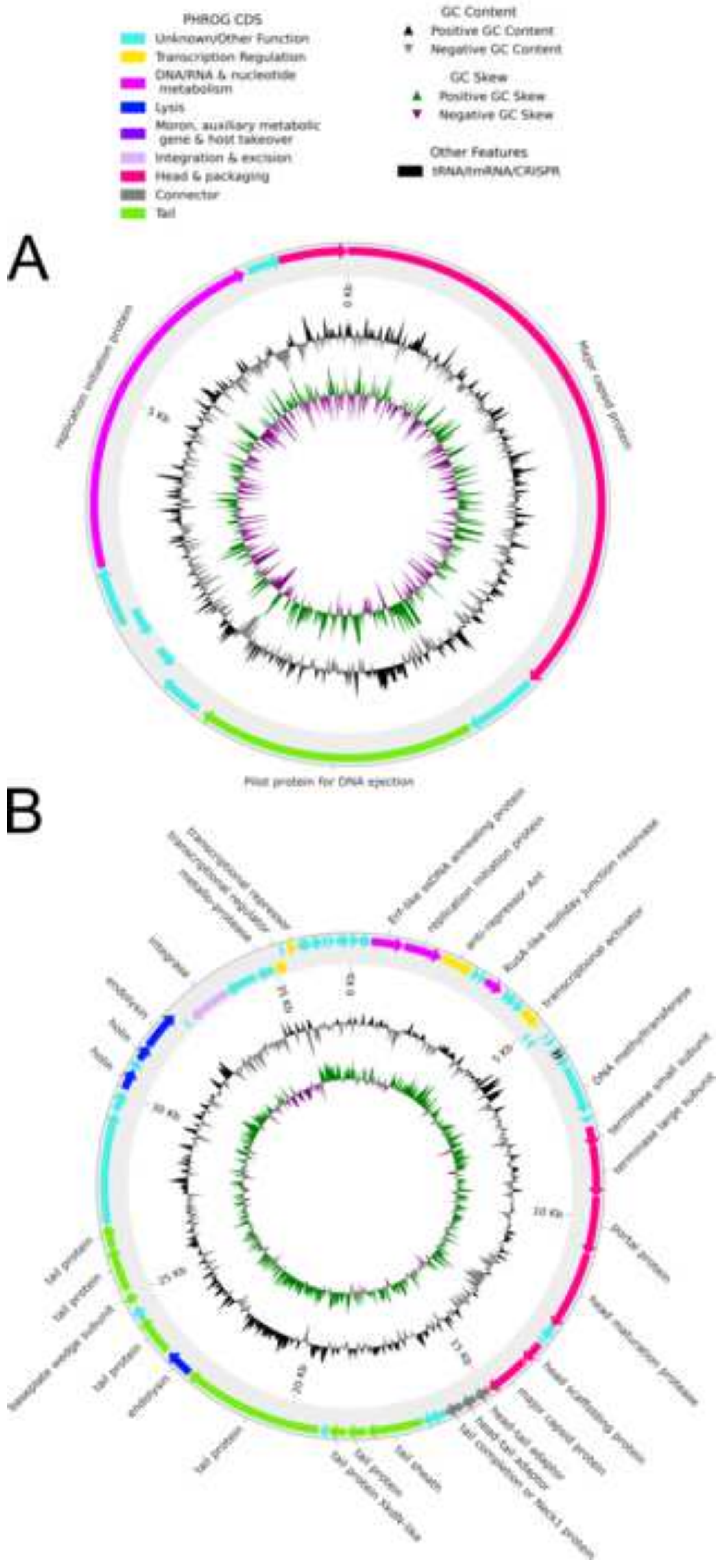

Figure_S1 Click here to access/download;Figure;FigS1.pdf

	Released	Packaged install	Recently updated	Short reads	Longreads	Round A/B	Human host remove	Custom host remove	Read QC	Read annotations	Assembly	Contig annotations	Eukaryotic viruses	Phages
**Hecatomb**	2022	✓	✓	✓	✓	✓	✓	✓	✓	✓	✓	✓	✓	✓
**INHERIT**	2022	X	X	X	✓	X	X	X	X	X	X	✓	X	✓
**MetaPhage**	2022	X	✓	✓	X	X	X	X	X	X	✓	✓	X	✓
**Seq2Vec**	2022	X	✓	X	X	X	X	X	X	X	X	X	✓	✓
**What the Phage**	2022	X	✓	X	X	X	X	X	X	X	X	✓	X	✓
**VirSorter2**	2021	✓	✓	X	✓	X	X	X	X	✓	X	✓	✓	✓
**Cenote-Taker 2**	2020	X	✓	✓	X	X	X	X	✓	X	✓	✓	✓	✓
**DeepVirFinder**	2020	X	X	X	✓	X	X	X	X	X	X	✓	✓	✓
**LAZYPIPE**	2020	X	✓	✓	X	X	✓	✓	✓	X	✓	✓	✓	✓
**Seeker**	2020	✓	X	X	✓	X	X	X	X	X	X	✓	X	✓
**VIBRANT**	2020	X	X	X	✓	X	X	X	X	X	X	✓	✓	✓
**PhaMers**	2019	X	X	X	X	X	X	X	X	X	X	✓	X	✓
**PPR-Meta**	2019	X	X	X	X	X	X	X	X	X	X	✓	X	✓
**vContact2**	2019	✓	✓	X	X	X	X	X	X	X	X	✓	X	✓
**VFM**	2019	X	X	X	X	X	X	X	X	X	X	✓	X	✓
**ViraMiner**	2019	X	X	X	X	X	X	X	X	X	X	X	✓	✓
**virMine**	2019	X	✓	✓	X	X	X	X	✓	X	✓	✓	✓	✓
**virMiner**	2019	X	X	✓	X	X	X	X	✓	X	X	✓	X	✓
**FastViromeExplorer**	2018	X	X	✓	X	X	X	X	X	✓	X	X	✓	✓
**VirNet**	2018	X	X	✓	✓	X	X	X	X	X	X	X	X	✓
**VirFinder**	2017	✓	X	X	✓	X	X	X	X	✓	X	✓	✓	✓
**HoloVir**	2016	X	X	✓	X	X	X	X	✓	✓	✓	✓	✓	✓
**MetaPhinder**	2016	X	X	X	X	X	X	X	X	X	X	✓	X	✓
**VIP**	2016	X	X	✓	X	X	X	X	✓	✓	X	X	✓	✓
**VirSorter**	2015	X	X	X	X	X	X	X	X	X	X	✓	X	✓

Click here to access/download;Figure;FigS2.pdf

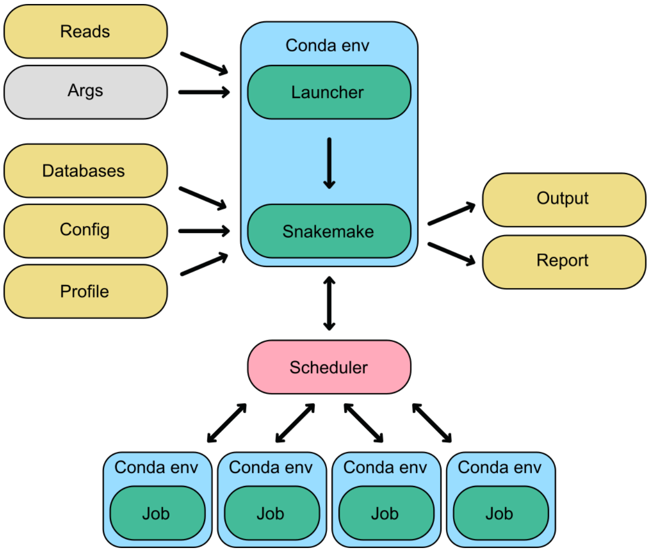

Click here to access/download;Figure;FigS3.pdf

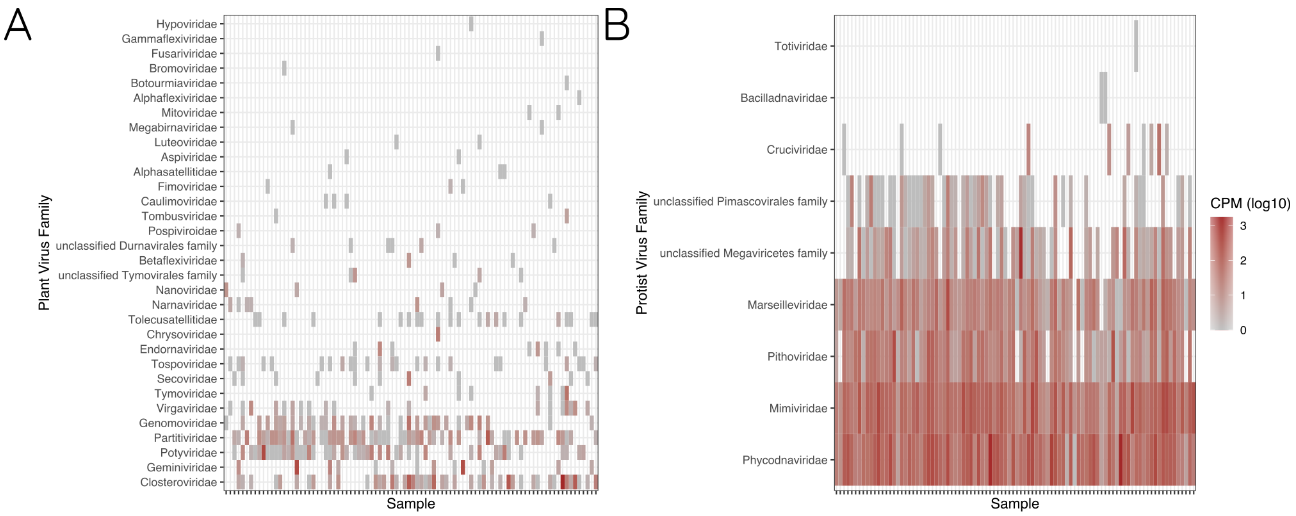

 Click here to access/download;Figure;FigS4.pdf

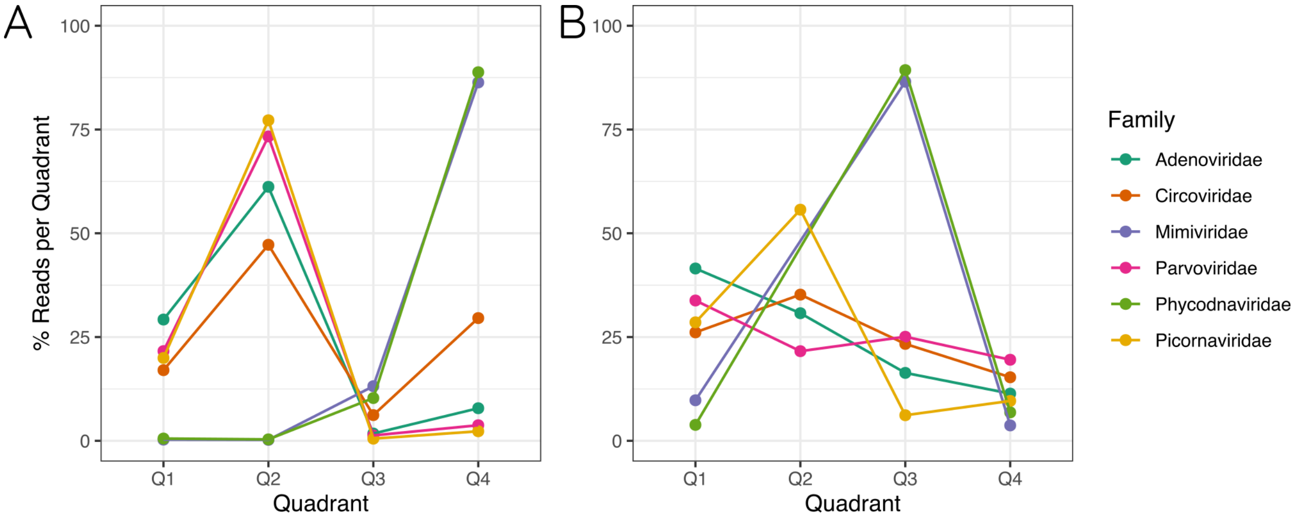

Click here to access/download;Figure;FigS5_revised.pdf

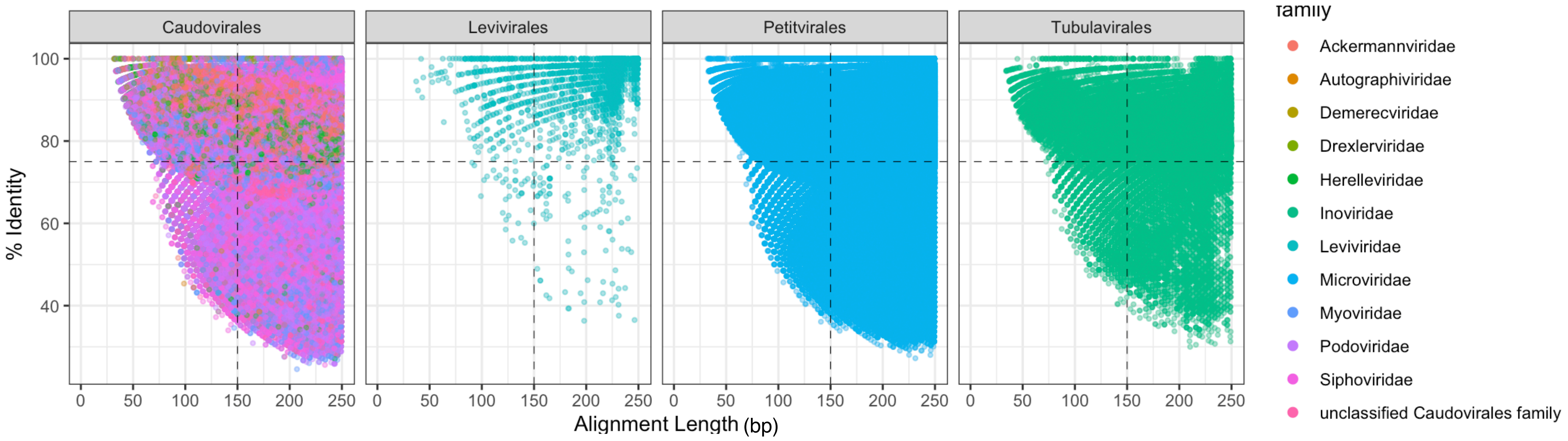

Click here to access/download
Supplementary Material
hecatomb_supplementary_methods.docx

Table_S2

Click here to access/download
Supplementary Material
table_S2.xlsx
