# Supplementary material for: Hecatomb: an integrated software platform for viral metagenomics
Source: Gigascience. 2024 Jun 4;13:giae020. doi: 10.1093/gigascience/giae020 (PMC11148595; doi:10.1093/gigascience/giae020)
Supplement: giae020_Supplemental_Files [file giae020_supplemental_files.zip › hecatomb_supplementary_methods.docx]

# Supplementary Methods

**Hecatomb databases.** Hecatomb databases are hosted in the cloud with Amazon Web Services (AWS). Downloading and installing them is managed by the Hecatomb launcher. The most up to date descriptions of the databases can be obtained from Hecatomb’s documentation at [hecatomb.readthedocs.io](https://hecatomb.readthedocs.io/en/latest/#databases).

1. **Contaminants**. This database consists of a collection of NEBNext and TruSeq sequencing adapters, primers, and vector contaminants from UniVec [[106]](https://paperpile.com/c/9GO7Dg/rcUml). The contaminants database is used exclusively during sequence preprocessing.
2. **Hosts**. A collection of host genomes that have been preprocessed to mask viral-like and low-entropy sequences. Host genomes are used for host read removal during preprocessing. Hecatomb comes with several common host genomes ready to use, and users can add their own host genomes to the database via the Hecatomb launcher.
3. **AA and NT**. These are the amino acid (AA) and nucleotide (NT) databases used for sequence annotation of both reads and contigs. For each of the AA and NT databases, there is a primary viral database used for classifying reads that match a known virus, and a secondary multi-kingdom database which is used for assigning taxonomy to either reads and contigs. The primary AA database includes all UniProt viral protein entries clustered at 99% identity. The secondary AA database consists of the Uniclust50 database [[77]](https://paperpile.com/c/9GO7Dg/i7gxY) ([doi.org/10.1093/nar/gkw1081](https://doi.org/10.1093/nar/gkw1081)) supplemented with the primary AA database. The primary NT database consists of all viral sequences in GenBank clustered at 100% identity to remove redundancy. The secondary NT database consists of a customized polymicrobial nucleotide database containing representative RefSeq genomes from Bacteria (n = 14,933), Archaea (n = 511), Fungi (n = 423), Protozoa (n = 90) and plant (n = 145) genomes.
4. **Tax**. This is NCBI’s taxonomy database used for translating taxonomy IDs into full taxonomic lineages [[79]](https://paperpile.com/c/9GO7Dg/2mpRi).
5. **Tables**. These are additional classification tables such as the ICTV 2019 Baltimore groups for viruses, which are used to supplement the output annotation tables.

**Hecatomb outputs.** The most up-to-date descriptions of all the pipeline output files can be obtained from Hecatomb’s documentation at [hecatomb.readthedocs.io](https://hecatomb.readthedocs.io/en/latest/#file-outputs). A tutorial is included to demonstrate typical analyses based on these output files using both R or Python. The main output files are:

1. **Report**. Hecatomb utilizes a Snakemake feature to generate a report (*report.html*) of the pipeline run. The report includes the pipeline rule graph, details about resource usage and runtime for each rule, and the used configuration file. The report also includes read counts generated following every preprocessing and annotation step rendered as a Sankey diagram.
2. **Seqtable**. The sequence table (*seqtable.fasta*) contains all representative sequences for the clustered reads for all samples. The sample names and cluster counts are incorporated into the sequence IDs whilst remaining in a standard sequential multi-fasta format.
3. **Read annotations**. The read annotations table (*bigtable.tsv*) is used for the majority of analyses from Hecatomb. This table combines the sequence and sample IDs, cluster counts, alignment metrics, taxonomic and available functional annotations into one table. This table is designed to expedite downstream analysis of the read annotations using commonly used software platforms (Python, R, BASH, Excel).
4. **Assembly**. The population assembly (*assembly.fasta*) is the output of contigs obtained by Flye. Contigs are annotated using MMseqs2 queries against viral and other genomes obtained from the secondary nucleotide database [[107]](https://paperpile.com/c/9GO7Dg/xmoiH).
5. **Contig annotations**. Contig taxonomic annotations (*contigAnnotations.tsv*) are calculated using MMSeqs2 [[48]](https://paperpile.com/c/9GO7Dg/m1UZM) querying each contig against Hecatombs’ multi-kingdom NT database (described above). Read-based contig annotations (*contigSeqTable.tsv*) are also generated by mapping individual read annotations positionally to each contig.
6. **Contig coverage**. Coverage statistics of each population assembled contig (*contig_count_table.tsv*) are calculated by mapping reads from each sample to all contigs using BBMap (<https://sourceforge.net/projects/bbmap/>). RPKM, FPKM, and SPM are subsequently calculated (see above Supplemental Methods for calculations). A read-based contig annotations file (*contigSeqTable.tsv*) can also be used to retrieve mapped read counts of contigs for each sample.

###

### Table S1. Software packages used by Hecatomb.

| **Package name** | **Version** | **URL** | **Reference** |
| --- | --- | --- | --- |
| Conda | 4.10.3 | conda.io |  |
| Mamba | 0.17.0 | github.com/mamba-org/mamba |  |
| Python3 | 3.10.0 | python.org |  |
| Snaketool | 9d81e2b | github.com/beardymcjohnface/Snaketool | [[89]](https://paperpile.com/c/9GO7Dg/P1X1J) |
| Snakemake | 6.4.1 | snakemake.github.io | [[90]](https://paperpile.com/c/9GO7Dg/6O0cB) |
| fastp | 0.23.2 | github.com/OpenGene/fastp | [[71]](https://paperpile.com/c/9GO7Dg/BGl2y) |
| PySam | 0.17.0 | github.com/pysam-developers/pysam |  |
| BBTools | 38.90 | jgi.doe.gov/data-and-tools/bbtools | [[84]](https://paperpile.com/c/9GO7Dg/y7nBR) |
| MEGAHIT | 1.2.9 | github.com/voutcn/megahit | [[81]](https://paperpile.com/c/9GO7Dg/6GB3U) |
| Canu | 2.2 | github.com/marbl/canu | [[82]](https://paperpile.com/c/9GO7Dg/H4wzH) |
| Flye | 2.7.1 | github.com/fenderglass/Flye | [[83]](https://paperpile.com/c/9GO7Dg/Ff6tf) |
| Minimap2 | 2.20 | github.com/lh3/minimap2 | [[74]](https://paperpile.com/c/9GO7Dg/GOtMp) |
| Samtools | 1.13 | github.com/samtools/samtools | [[91]](https://paperpile.com/c/9GO7Dg/L9bVX) |
| MMseqs2 | 12.113e3 | github.com/soedinglab/MMseqs2 | [[48]](https://paperpile.com/c/9GO7Dg/m1UZM) |
| TaxonKit | 0.8.0 | github.com/shenwei356/taxonkit | [[80]](https://paperpile.com/c/9GO7Dg/UsTYm) |
| Plotly | 5.3.1 | plotly.com |  |
| Kaleido | 0.2.1 | pypi.org/project/kaleido |  |
| SeqKit | 0.16.1 | github.com/shenwei356/seqkit | [[92]](https://paperpile.com/c/9GO7Dg/IcNKP) |

###

### Table S2. Complete, high-quality phage genomes recovered using Hecatomb and Phables.

| **Accession** | **Assembly name** | **Best hit identity (%)** | **Sequences from** |
| --- | --- | --- | --- |
| GCA_951573495 | uncultured Microviridae phage 1018C1 | 90.1 | Handley et al. (2016) |
| GCA_951571905 | uncultured Microviridae phage 1024C1 | 71.8 | Handley et al. (2016) |
| GCA_951571845 | uncultured Microviridae phage 1038C1 | 91.2 | Handley et al. (2016) |
| GCA_951571955 | uncultured Microviridae phage 1052C1 | 75.4 | Handley et al. (2016) |
| GCA_951571815 | uncultured Microviridae phage 1070C1 | 79.3 | Handley et al. (2016) |
| GCA_951572865 | uncultured Microviridae phage 1092C1 | 76.2 | Handley et al. (2016) |
| GCA_951571655 | uncultured Microviridae phage 1092C2 | 76.2 | Handley et al. (2016) |
| GCA_951573235 | uncultured Caudoviricetes phage 1112C1 | 88.8 | Handley et al. (2016) |
| GCA_951571515 | uncultured Microviridae phage 1113C1 | 78.9 | Handley et al. (2016) |
| GCA_951572005 | uncultured Microviridae phage 1138C1 | 72.3 | Handley et al. (2016) |
| GCA_951572065 | uncultured Microviridae phage 1145C1 | 88.9 | Handley et al. (2016) |
| GCA_951572755 | uncultured Microviridae phage 1197C1 | 77.0 | Handley et al. (2016) |
| GCA_951571045 | uncultured Microviridae phage 1197C2 | 77.0 | Handley et al. (2016) |
| GCA_951572745 | uncultured Microviridae phage 1198C1 | 89.2 | Handley et al. (2016) |
| GCA_951572475 | uncultured Microviridae phage 1220C1 | 74.0 | Handley et al. (2016) |
| GCA_951570655 | uncultured Microviridae phage 1221C1 | 69.6 | Handley et al. (2016) |
| GCA_951571025 | uncultured Microviridae phage 1229C1 | 80.2 | Handley et al. (2016) |
| GCA_951573305 | uncultured Microviridae phage 1235C1 | 82.6 | Handley et al. (2016) |
| GCA_951573075 | uncultured Caudoviricetes phage 123C1 | 71.6 | Handley et al. (2016) |
| GCA_951573255 | uncultured Microviridae phage 1244C1 | 76.0 | Handley et al. (2016) |
| GCA_951572305 | uncultured Microviridae phage 1245C1 | 68.1 | Handley et al. (2016) |
| GCA_951573485 | uncultured Microviridae phage 1245C2 | 68.5 | Handley et al. (2016) |
| GCA_951570695 | uncultured Microviridae phage 1252C1 | 67.5 | Handley et al. (2016) |
| GCA_951573205 | uncultured Microviridae phage 1254C1 | 71.1 | Handley et al. (2016) |
| GCA_951573195 | uncultured Microviridae phage 1255C1 | 72.0 | Handley et al. (2016) |
| GCA_951573275 | uncultured Microviridae phage 1261C1 | 82.5 | Handley et al. (2016) |
| GCA_951572215 | uncultured Microviridae phage 1269C1 | 87.2 | Handley et al. (2016) |
| GCA_951572705 | uncultured Microviridae phage 126C1 | 27.1* | Handley et al. (2016) |
| GCA_951572585 | uncultured Gokushovirinae phage 1274C1 | 91.6 | Handley et al. (2016) |
| GCA_951571365 | uncultured Microviridae phage 1295C1 | 84.4 | Handley et al. (2016) |
| GCA_951573635 | uncultured Microviridae phage 1296C1 | 76.0 | Handley et al. (2016) |
| GCA_951572785 | uncultured Microviridae phage 1297C1 | 73.1 | Handley et al. (2016) |
| GCA_951573435 | uncultured Microviridae phage 1298C1 | 74.4 | Handley et al. (2016) |
| GCA_951572835 | uncultured Microviridae phage 1299C1 | 73.3 | Handley et al. (2016) |
| GCA_951573165 | uncultured Microviridae phage 1300C1 | 72.2 | Handley et al. (2016) |
| GCA_951573315 | uncultured Microviridae phage 1301C1 | 88.0 | Handley et al. (2016) |
| GCA_951573555 | uncultured Microviridae phage 1302C1 | 77.9 | Handley et al. (2016) |
| GCA_951573565 | uncultured Microviridae phage 1303C1 | 80.9 | Handley et al. (2016) |
| GCA_951571285 | uncultured Microviridae phage 1304C1 | 70.4 | Handley et al. (2016) |
| GCA_951572245 | uncultured Microviridae phage 1305C1 | 79.0 | Handley et al. (2016) |
| GCA_951573015 | uncultured phage 1306C1 | 92.8* | Handley et al. (2016) |
| GCA_951572935 | uncultured Microviridae phage 1307C1 | 71.6 | Handley et al. (2016) |
| GCA_951573085 | uncultured Cressdnaviricota virus 1308C1 | 76.8 | Handley et al. (2016) |
| GCA_951572185 | uncultured Microviridae phage 1309C1 | 68.6 | Handley et al. (2016) |
| GCA_951572295 | uncultured Microviridae phage 1310C1 | 67.9 | Handley et al. (2016) |
| GCA_951572925 | uncultured Microviridae phage 1311C1 | 92.4 | Handley et al. (2016) |
| GCA_951573355 | uncultured Microviridae phage 1312C1 | 70.0 | Handley et al. (2016) |
| GCA_951571465 | uncultured Microviridae phage 1313C1 | 74.6 | Handley et al. (2016) |
| GCA_951572825 | uncultured Microviridae phage 1314C1 | 80.5 | Handley et al. (2016) |
| GCA_951572915 | uncultured Microviridae phage 1315C1 | 85.7 | Handley et al. (2016) |
| GCA_951570725 | uncultured Microviridae phage 1316C1 | 81.6 | Handley et al. (2016) |
| GCA_951572875 | uncultured Microviridae phage 1317C1 | 72.4 | Handley et al. (2016) |
| GCA_951571075 | uncultured phage 1319C1 | 69.7 | Handley et al. (2016) |
| GCA_951572375 | uncultured phage 1320C1 | 75.2 | Handley et al. (2016) |
| GCA_951573725 | uncultured Microviridae phage 1322C1 | 77.9 | Handley et al. (2016) |
| GCA_951572905 | uncultured Gokushovirinae phage 1332C1 | 91.2 | Handley et al. (2016) |
| GCA_951572365 | uncultured Microviridae phage 1335C1 | 72.3 | Handley et al. (2016) |
| GCA_951572105 | uncultured Microviridae phage 1336C1 | 77.3 | Handley et al. (2016) |
| GCA_951572425 | uncultured Microviridae phage 1337C1 | 73.8 | Handley et al. (2016) |
| GCA_951572385 | uncultured Gokushovirinae phage 1338C1 | 90.5 | Handley et al. (2016) |
| GCA_951572395 | uncultured Microviridae phage 1339C1 | 92.1 | Handley et al. (2016) |
| GCA_951572335 | uncultured Microviridae phage 1340C1 | 92.6* | Handley et al. (2016) |
| GCA_951572405 | uncultured Microviridae phage 1342C1 | 70.3 | Handley et al. (2016) |
| GCA_951573765 | uncultured Microviridae phage 1343C1 | 71.1 | Handley et al. (2016) |
| GCA_951571085 | uncultured Microviridae phage 1344C1 | 70.4 | Handley et al. (2016) |
| GCA_951572345 | uncultured Microviridae phage 1345C1 | 71.6 | Handley et al. (2016) |
| GCA_951573695 | uncultured Microviridae phage 1346C1 | 77.0 | Handley et al. (2016) |
| GCA_951573645 | uncultured Microviridae phage 1347C1 | 92.8 | Handley et al. (2016) |
| GCA_951571135 | uncultured Microviridae phage 1348C1 | 99.7* | Handley et al. (2016) |
| GCA_951572545 | uncultured Studiervirinae phage 1349C1 | 69.6 | Handley et al. (2016) |
| GCA_951571935 | uncultured Microviridae phage 1351C1 | 87.8 | Handley et al. (2016) |
| GCA_951572645 | uncultured Cressdnaviricota virus 191C1 | 78.3 | Handley et al. (2016) |
| GCA_951571595 | uncultured Microviridae phage 210C1 | 75.1 | Handley et al. (2016) |
| GCA_951572015 | uncultured phage 236C1 | 94.8* | Handley et al. (2016) |
| GCA_951571885 | uncultured phage 236C2 | 94.8* | Handley et al. (2016) |
| GCA_951571965 | uncultured Microviridae phage 260C1 | 91.1 | Handley et al. (2016) |
| GCA_951575695 | uncultured Microviridae phage 260C2 | 91.1 | Handley et al. (2016) |
| GCA_951575175 | uncultured Twortvirinae phage 27C1 | 75.7 | Handley et al. (2016) |
| GCA_951575205 | uncultured phage 27C2 | 97.7* | Handley et al. (2016) |
| GCA_951576275 | uncultured Microviridae phage 325C1 | 72.1 | Handley et al. (2016) |
| GCA_951574955 | uncultured Microviridae phage 346C1 | 70.4 | Handley et al. (2016) |
| GCA_951576375 | uncultured Microviridae phage 348C1 | 77.5 | Handley et al. (2016) |
| GCA_951575165 | uncultured Microviridae phage 386C1 | 78.9 | Handley et al. (2016) |
| GCA_951575085 | uncultured phage 400C1 | 71.0 | Handley et al. (2016) |
| GCA_951575275 | uncultured phage 400C2 | 71.0 | Handley et al. (2016) |
| GCA_951574225 | uncultured Caudoviricetes phage 417C1 | 74.5 | Handley et al. (2016) |
| GCA_951574215 | uncultured Caudoviricetes phage 417C2 | 74.5 | Handley et al. (2016) |
| GCA_951574575 | uncultured Microviridae phage 425C1 | 87.5 | Handley et al. (2016) |
| GCA_951574235 | uncultured Caudoviricetes phage 443C1 | 68.7 | Handley et al. (2016) |
| GCA_951575015 | uncultured Microviridae phage 462C1 | 70.8 | Handley et al. (2016) |
| GCA_951574965 | uncultured Microviridae phage 462C2 | 68.6 | Handley et al. (2016) |
| GCA_951574605 | uncultured Microviridae phage 511C1 | 88.0 | Handley et al. (2016) |
| GCA_951576195 | uncultured Microviridae phage 527C1 | 74.1 | Handley et al. (2016) |
| GCA_951574005 | uncultured Microviridae phage 534C1 | 71.5 | Handley et al. (2016) |
| GCA_951576365 | uncultured Microviridae phage 601C1 | 75.1 | Handley et al. (2016) |
| GCA_951574665 | uncultured Microviridae phage 609C1 | 79.7 | Handley et al. (2016) |
| GCA_951575005 | uncultured Microviridae phage 630C1 | 87.1 | Handley et al. (2016) |
| GCA_951574985 | uncultured Microviridae phage 660C1 | 93.6 | Handley et al. (2016) |
| GCA_951573825 | uncultured Microviridae phage 68C1 | 69.0 | Handley et al. (2016) |
| GCA_951574995 | uncultured phage 696C1 | 71.8 | Handley et al. (2016) |
| GCA_951575985 | uncultured phage 696C2 | 72.4 | Handley et al. (2016) |
| GCA_951575025 | uncultured Firehammervirus phage 722C1 | 72.9 | Handley et al. (2016) |
| GCA_951573855 | uncultured Microviridae phage 726C1 | 75.2 | Handley et al. (2016) |
| GCA_951575345 | uncultured phage 72C1 | 68.5 | Handley et al. (2016) |
| GCA_951574435 | uncultured phage 72C2 | 68.5 | Handley et al. (2016) |
| GCA_951574905 | uncultured Caudoviricetes phage 745C1 | 69.2 | Handley et al. (2016) |
| GCA_951574505 | uncultured Caudoviricetes phage 745C2 | 69.2 | Handley et al. (2016) |
| GCA_951574945 | uncultured Microviridae phage 769C1 | 77.7 | Handley et al. (2016) |
| GCA_951574735 | uncultured Microviridae phage 784C1 | 67.0 | Handley et al. (2016) |
| GCA_951574315 | uncultured Microviridae phage 812C1 | 71.5 | Handley et al. (2016) |
| GCA_951574625 | uncultured Microviridae phage 822C1 | 88.0 | Handley et al. (2016) |
| GCA_951575795 | uncultured Microviridae phage 849C1 | 78.0 | Handley et al. (2016) |
| GCA_951575855 | uncultured Microviridae phage 869C1 | 75.6 | Handley et al. (2016) |
| GCA_951574085 | uncultured Microviridae phage 941C1 | 73.3 | Handley et al. (2016) |
| GCA_951575815 | uncultured Microviridae phage 941C2 | 70.3 | Handley et al. (2016) |
| GCA_951575875 | uncultured Microviridae phage 950C1 | 73.3 | Handley et al. (2016) |
| GCA_951573885 | uncultured Microviridae phage 952C1 | 73.0 | Handley et al. (2016) |
| GCA_951576095 | uncultured Microviridae phage 977C1 | 93.2 | Handley et al. (2016) |
| GCA_951573975 | uncultured Microviridae phage 992C1 | 85.1 | Handley et al. (2016) |
| GCA_951574175 | uncultured Microviridae phage 998C1 | 74.0 | Handley et al. (2016) |
| GCA_951576125 | uncultured Microviridae phage 998C2 | 72.0 | Handley et al. (2016) |
| OQ984149 | uncultured Caudoviricetes phage C48C1 | 73.9 | Lima et al. (2021) |
| OQ984150 | uncultured Caudoviricetes phage C72C1 | 67.7 | Lima et al. (2021) |
| OQ984151 | uncultured Caudoviricetes phage C75C1 | 71.5 | Lima et al. (2021) |

* No nucleotide hits, translated amino acid alignment identity instead
