# Supplementary material for: Hecatomb: an integrated software platform for viral metagenomics
Source: Gigascience. 2024 Jun 4;13:giae020. doi: 10.1093/gigascience/giae020 (PMC11148595; doi:10.1093/gigascience/giae020)
Supplement: giae020_Response_to_Reviewer_Comments_Original_Submission [file giae020_response_to_reviewer_comments_original_submission.pdf]

Author's Response To Reviewer Comments

Responses to review of “Hecatomb: An Integrated Software Platform for Viral Metagenomics”

Reviewer 1:

The MS titled "Hecatomb: An Integrated Software Platform for Viral Metagenomics" addresses the developed of a toolkit for viral meatgenomics analysis that assembles a variety of tools into a workflow. Overall, I do not have any issue with this MS or the toolkit. I have some minor points to help improve the MS and make it as current as possible.

1. Line 40: I would include Cenote-take 2 PMID: 33505708, geNomad https://www.biorxiv.org/content/10.1101/2023.03.05.531206v1

These citations have been included.

2. Line 40: I would probably not cite the preprint of this current paper - see ref 21.

This was an oversight, and the reference has been removed.

3. Line 80: Actually Cenote-take (both version 1 and 2) both use HHMs and as far as I know so does geNomad.

We have updated the text to indicate the Cenote-taker2 and geNomad also use HMM’s.

4. Line 248: Please note that Siphoviridae, Podoviridae and Myoviridae are not currently family names. See PMID: 36683075

We have removed reference to the Myo-, Podo- and Siphoviridae and have collapsed these taxonomies into the Caudovirales.

5. This means you will likely need to edit you figure to collapse these to Caudovirales

The Myo-, Podo- and Siphoviridae have been collapsed in Figure 4A.

6. Line 250-251: Picornaviridae and Adenoviridiae should be in italics

This has been updated to italics.

7. Line 270: Here and elsewhere, please note that a taxa do not infect a host, it is a virus that infects a host. "Mimiviridae, that infect Acanthamoeba, and Phycodnaviridae, that infect algae, are both dsDNA viruses with large genomes" should ideally be written as "Viruses in the family Mimiviridae infect Acanthamoeba and those in the family Phycodnavirida infect algae, are dsDNA viruses with large genomes."

The language has been updated appropriately.

8. Figure 6: the name tags of the CDS/ ORFS are truncated e.g. replication initiate…, heat maturation prot…

The name tags have been updated appropriately.

9. Figure 6: Major head protein should be major capsid protein.

The language has been updated appropriately.

10. One thing that I would highlight is that none of the workflows / tool kits developed account for spliced CDS. This is a major issue in automation of virus genome annotation at the moment and with this there will be some degree of misidentification for taxa assignment.

We agree, and in addition, believe that there are several future advancements that will facilitate a more comprehensive virome analysis and viral gene/genome annotaitons. We noted this in the final section "Potential Implications" with the following language:

"Virome analysis is an evolving field requiring novel approaches for comprehensive characterization. For example, none of the described workflows account for spliced coding sequences (CDS), hampering the automation of viral genome annotation. The modularity of Hecatombs approach should enable easy integration of novel methods as they emerge."

Reviewer 2:

In this manuscript, the authors developed a novel pipeline, Hecatomb, for viral genome analysis using metagenome and virome data that accepted both short- and long-read sequencing data. Using the pipeline, the authors performed the analysis using one virome and one metagenome dataset from different environments (stool and coral reef, respectively). The analyses showed reasonable results according to the original studies and rather they discovered candidate novel phages and new findings that possibly have great insight into the microbial ecology. The manuscript is overall informative and well-written. The Hecatomb incorporates famous bioinformatics tools that are frequently used in viral genome analyses today, allowing many researchers including beginners to examine virome datasets easily and effectively. Thus the pipeline is likely valuable and would contribute to wide studies of viruses, most of which are not cultured and its characteristics are unknown. Noteworthy, there is an informative document page (https://hecatomb.readthedocs.io/en/latest/) including tutorials, which are very helpful for many users. I think this point could be more emphasized in the manuscript. However, unfortunately, lacking the analysis of the mock dataset makes it hard to estimate the accuracy of the pipeline. I think adding such kinds of analysis for evaluating the performance would greatly improve the study. I have some suggestions that would increase the clarity and impact of this manuscript if addressed.

Major: In general, to clearly evaluate the efficiency of the novel bioinformatic tools and pipelines, benchmarking using ground-truth datasets is important in advance to the application using real datasets. To reach this, in this case, some artificial datasets that are composed of known viral and prokaryotic genomes with defined composition and library types (single and paired-end) and sequenced read length (current short- and long-reads) could be designed as mock metagenome data. Via the analysis using the mock datasets, the accuracy of the pipeline can be evaluated. It would be appreciated if the author performed such benchmarking tests as well as the real data applications. According to the GitHub page, the Hecatomb is designed to generate results that reduce false-positive and enrich for true-positive viral read detection. This point is important for understanding the purpose of developing the pipeline and differentiating the pipeline tool from other ones. The efficiency of the false-positive reduction using this pipeline would be better clearly shown in this manuscript. Therefore the mock dataset analyses are expected.

To address this concern we generated a mock, in silico data set as follows:

Canonical viral genomes from each genomic type (single and double-stranded DNA and RNA genomes and one retrovirus) were downloaded from NCBI. For each of these genomes we simulated 1 x 104 250 base pair sequences using the Illumina error-model model available in InSilicoSeq (Gourlé et al. 2019). We next similar generated 1 x 106 sequences from a collection of 1,520 cultivated human gut bacterial genomes (Zou et al. 2019) using the same Illumina error-model. Simulated sequences from viruses and bacteria were combined to simulate a mixed virus-bacteria environment common to many studies and processed through Hecatomb using the default settings. Hecatomb assigned the correct taxonomy to 99.64-99.65% of the simulated viral reads with a sensitivity ranging from 0.98-1 and a false-positive rate ranging from 8.8 x 10-4 to 1.35 x 10-3 (Table 1).

When I read the manuscript, I was confused about what the targeted dataset the pipeline aiming for. Is the Hecatomb designed to analyze common prokaryotic shotgun metagenomic data to detect viruses? In other words, is the pipeline not limited to analyzing viral metagenomes (viromes), which specifically enriched viral particles from the samples for sequencing (e.g., density centrifugation to condense viral particles)? The stool samples were likely virome datasets (viral particles were enriched via 0.45-μm-pore-size membrane filtration according to the article), whereas the coral reef data are metagenome datasets. I would suggest that the terms "viral metagenome" (or virome, specifically targeting only viruses) and common "metagenome" (mainly focusing on prokaryotes) should be clearly distinguished throughout the manuscript including the title.

Hecatomb can annotate viral reads from both viral preparations and metagenomes. As the reviewer has noted, this is part of the rationale for using both types of data sets in the analysis presented in the manuscript. We noted that the re-analysis of the SIV Macaque data was generated from virus-like particle (VLP) preparations and that the coral reef data set was generated from non-VLP-prepped metagenomic data to clarify this.

I'm wondering about the sequence clustering step in Module 1. In my understanding, from the metagenomic settings, genomic regions are randomly sequenced, and thus most of the sequenced reads will not be clustered together using the criteria as described in the manuscript, and not so many sequences are reduced in this step. Is this step truly needed?

Please add more explanation and importance about this step. For example, how many ratios of the reads were reduced in the test of the two real datasets (stool and coral reef) in this step?

Clustering is a useful pre-processing procedure for processing low-diversity viral libraries, such as those that would be generated from virus-like particle (VLP) preparations. These preparations generate a lot of redundant sequence information due to their low complexity and small genomes and the many rounds of PCR that are typically used before library prep due to the limited amounts of available nucleic acid. It can therefore be valuable to 1) remove exact duplicates that are likely PCR-derived and 2) remove highly redundant sequences before the more computationally expensive procedures like mmseqs2 searches against large databases.

However, this is not fundamentally necessary and would be a lower value given the high diversity libraries. To better demonstrate the levels of sequence redundancy following VLP preparation we have added a table (Table S2) for the SIV re-analysis data set showing dereplication ranges from 4-56% across samples. Of note, as mentioned in the manuscript, this process is easily tunable by users using the YAML file and can be completely turned off if deemed unnecessary.

Minor: The introduction section is informative but a bit long. The section could be shortened.

Some viruses were newly found using the pipeline (e.g., Fig1A). Which one is which virus types (dsDNA, ssDNA, dsRNA, ssRNA)? This information would be better to show clearly in the figure.

(Note: We assume the reviewer is referring to Figure 4A not 1A): One of Hecatombs main utilities is in how it combines data types to provide rich plotting options for the end user. One possible annotation is virus genome type as the reviewer suggests. However, we believe that Figure 4A is the best representation on how Hecatomb can take advantage of these rich data and would prefer not to ‘overplot’ in addition to Phylum, Family and virus-type (phage, plant, etc.). Therefore while we agree it would be valuable to show any number of data-overlays but prefer to leave it as is to not overwhelm the reader.

We believe that the addition of the in silico analysis suggested by the reviewer which demonstrates results examining ss/ds DNA and RNA viruses now highlights that this is an option to the end user.

I think the sequences derived from RNA viruses are generally not abundantly included in typical metagenomics datasets except if with specific techniques in the experiment. I think the potential for detecting RNA viruses from typical metagenomic DNA sequencing reads will be discussed in the Introduction section.

We added the following comment to the introuction:

“In addition, researchers must contend with how to annotate viral sequences from viruses with either RNA or DNA based genomes.”

We prefer not to elaborate on this much further as the major factor influencing ones ability to detect RNA viruses occurs in the wet lab, not during computational annotation. So it is the study design step that precedes analysis by Hecatomb (the conversion of RNA to cDNA).

L103. Please describe where the name "Hecatomb" is derived from in this article, though this is shown on the GitHub page.

We have added the meaning as suggested.

L119. " round A/B libraries" here, but I have not heard or could not find this term in the articles cited here. Please add more explanation of what is "round A/B libraries".

We added the additional information about the round A/B procedure:

“… the round A/B library which converts RNA into cDNA using a tagged-randomized primer and second-strand DNA synthesis, the resulting dsDNA is subjected to 10-40 rounds of PCR amplification. This process generates DNA/cDNA libraries for single- and double-stranded RNA and DNA viruses [71–73].”

L130 up to 2 insertions and deletions?

Corrected to indicate insertions OR deletions.

L131. BBmap included in BBtools [73]?

Correct

L181. A brief explanation of the "Baltimore classification" here would improve the readability for readers who are not familiar with this.

Added the following:

“Baltimore is a classification system that places viruses into one of seven groups depending on a combination of their nucleic acid (DNA or RNA), strandedness (single-stranded or double-stranded), Sense, and method of replication.”

L239. There is no explanation of what "SIV" means before.

Added simian immunodeficiency virus.

L253-L268 & Figure 4B. According to Figure 2A, there are two paths (1,2,5: aa and 1,3,4,5: nt) for detecting viral reads. I'm interested in which path is major and which is minor. Could the authors provide the ratio of the reads that predicted using aa or nt in each dataset examination (each stool and coral)?

We have added a sentence in the SIV results as follows:

“Of the 2,394,740 reads annotated by hecatomb as viral, 1,989,352 (83%) were annotated using queries against protein (aa) databases and 405,388 (17%) annotated using queries against nucleotide databases (nt).”

L431, L436. Not only BioProject but SRA accession ID should be provided.

SRA Study Number SRP237459 was added for the coral reef data set. The SIV dataset does not have a single SRA number, In lieu of listing 95 individual SRA numbers we prefer to just use the BIoProject number. The SRA files are found when searching for the BioProject number.

L479. There is no LACC here. What is his main contribution? Just reviewing and editing the manuscript is insufficient for citing as an author: see https://www.icmje.org/recommendations/browse/roles-and-responsibilities/defining-the-role-of-authors-and-contributors.html#two

We added LACC where appropriate (software & validation, formal analysis).

Figure 1. There are some DBs newly created and used in the pipeline (e.g., Viral AA DB, Multi-kingdom AA DB, Virus NT DB, and Polymicrobial MT DB). I think it would be better to add how to make the DBs in this or other figures. This must contribute to understanding how to construct the DBs and why to use them in this pipeline.

The construction of these databases is supplied in text (see Module 2: Read-Based Annotation). Their construction is relatively straightforward (download and clustering) so we do not envision them fitting into Figure 1, or even their own figure and would prefer to leave them as is in the text.

Figure 1. specified (1)-(4) in the legend, not just color.

The numbers have been added to the figure legend.

Figure 4A. Please provide the total number of sequencing reads in addition to the read count assigned to each virus.

Figure 4A represents viral sequence annotated across the entire study (all 95 samples). In response to an earlier request from the reviewer, we have now included Table S2 which details the number of sequences (and dereplicated) sequences per sample. This amounts to a total of 2,394,740 sequences, as now noted in the legend for Figure 4A.

Figure 4C. CPM was not explained in the manuscript and not listed in L460.

A definition for counts per million (CPM) has been listed in L460 and in the legend to Figure 4.

L490. Some references are incomplete. e.g., lack of article ID or page number (49, 79, 90, 94, 95, 96, 100, 101, 102), remaining unnecessary words ("academic.oup.com" in 90, 91), etc. Please check the reference list carefully.

These have been reviewed and corrected.

Figure S5. Alignment length (bp)

This (bp) has been added to the x-axis label.

Table S2. For calculating the best hit identify, what database was used?

Added the following to clarify: (now table S3)

“We assigned taxonomy using MMSeqs2 with the Hecatomb primary nucleotide databases (Table S3)”
