# Supplementary material for: Hecatomb: an integrated software platform for viral metagenomics
Source: Gigascience. 2024 Jun 4;13:giae020. doi: 10.1093/gigascience/giae020 (PMC11148595; doi:10.1093/gigascience/giae020)
Supplement: giae020_Response_to_Reviewer_Comments_Revision_1 [file giae020_response_to_reviewer_comments_revision_1.pdf]

Author's Response To Reviewer Comments

Please address the following points:

1) Please include a citation to your new GigaDB dataset (including the DOI link) to your reference list, and cite this in the data availability section.

The citation is:

[xx] Roach MJ; Beecroft SJ; Mihindukulasuriya KA; Wang L; Paredes A; Chica Cardenas LA et al. Supporting data for "Hecatomb: An Integrated Software Platform for Viral Metagenomics" GigaScience Database. 2024 http://dx.doi.org/10.5524/102506

In the data availability section, please write something along the lines, "An archival copy of the code and supporting data is also available via the GigaScience repository, GigaDB [xx]".

-- DONE

2) Please register your new software application in the bio.tools and SciCrunch.org databases to receive RRID (Research Resource Identification Initiative ID) and biotoolsID identifiers, and include these in your manuscript (in the "availability" section, below the "licence " line). This will facilitate tracking, reproducibility and re-use of your tool.

-- DONE

3) Please move in-text URLs of code and data sources to the bibliography and cite them by number in the text (similar to the GigaDB reference) - please refer to our citation guidelines. - e.g. lines 131, 216, 217, 233, 234

-- DONE

4) please add ORCIDs to the title page. We have the following on our files:
ORCID iDs: Scott Handley [0000-0002-2143-6570]; Michael J Roach [0000-0003-1488-5148]; Sarah J Beecroft [0000-0002-3935-2279]; Kathie A Mihindukulasuriya [0000-0001-9372-3758]; Leran Wang [0009-0003-6928-9893]; Anne Paredes [0009-0009-1881-3855]; Luis Alberto Chica Cardenas [0009-0007-7408-9385]; Kara Henry-Cocks [0009-0004-5888-9372]; Lais Farias Oliveira Lima [0000-0002-7616-3637]; Elizabeth A Dinsdale [0000-0002-2177-203X]; Robert A Edwards [0000-0001-8383-8949];

-- DONE

5) Please check for minor typos etc. (I noticed "sankemake workflow" in the abstract, line 18.)

-- DONE
