# Supplementary material for: Hecatomb: an integrated software platform for viral metagenomics
Source: Gigascience. 2024 Jun 4;13:giae020. doi: 10.1093/gigascience/giae020 (PMC11148595; doi:10.1093/gigascience/giae020)
Supplement: giae020_Reviewer_1_Report_Original_Submission — Arvind Varsani -- 9/8/2023 Reviewed [file giae020_reviewer_1_report_original_submission.pdf]

Reviewer Report

Title: Hecatomb: An Integrated Software Platform for Viral Metagenomics

Version: Original Submission **Date:** 9/8/2023

Reviewer name: Arvind Varsani

Reviewer Comments to Author:

The MS titled "Hecatomb: An Integrated Software Platform for Viral Metagenomics" addresses the developed of a toolkit for viral meatgenomics analysis that assembles a variety of tools into a workflow.Overall, I do not have any issue with this MS or the toolkit.I have some minor points to help improve the MS and make it as current as possible.1. Line 40: I would include Cenote-take 2 PMID: 33505708, geNomad https://www.biorxiv.org/content/10.1101/2023.03.05.531206v12. Line 40: I would probably not cite the preprint of this current paper - see ref 21.3. Line 80: Actually Cenote-take (both version 1 and 2) both use HHMs and as far as I know so does geNomad.4. Line 248: Please note that Siphoviridae, Podoviridae and Myoviridae are not currently family names. See PMID: 366830755. This means you will likely need to edit you figure to collapse these to Caudovirales6. Line 250-251: Picornaviridae and Adenoviridiae should be in italics7. Line 270: Here and elsewhere, please note that a taxa do not infect a host, it is a virus that infects a host. "Mimiviridae, that infect Acanthamoeba, and Phycodnaviridae, that infect algae, are both dsDNA viruses with large genomes" should ideally be written as "Viruses in the family Mimiviridae infect Acanthamoeba and those in the family Phycodnavirida infect algae, are dsDNA viruses with large genomes."8. Figure 6: the name tags of the CDS/ ORFS are truncated e.g. replication initiate…, heat maturation prot…9. Figure 6: Major head protein should be major capsid protein.10. One thing that I would highlight is that none of the workflows / tool kits developed account for spliced CDS. This is a major issue in automation of virus genome annotation at the moment and with this there will be some degree of misidentification for taxa assignment.

Level of Interest

Please indicate how interesting you found the manuscript: Choose an item.

Quality of Written English

Please indicate the quality of language in the manuscript: Choose an item.

Declaration of Competing Interests

Please complete a declaration of competing interests, considering the following questions:

- Have you in the past five years received reimbursements, fees, funding, or salary from an organisation that may in any way gain or lose financially from the publication of this manuscript, either now or in the future?
- Do you hold any stocks or shares in an organisation that may in any way gain or lose financially from the publication of this manuscript, either now or in the future?
- Do you hold or are you currently applying for any patents relating to the content of the manuscript?
- Have you received reimbursements, fees, funding, or salary from an organization that holds or has applied for patents relating to the content of the manuscript?
- Do you have any other financial competing interests?
- Do you have any non-financial competing interests in relation to this paper?

If you can answer no to all of the above, write 'I declare that I have no competing interests' below. If your reply is yes to any, please give details below.

None of the above.

I agree to the open peer review policy of the journal. I understand that my name will be included on my report to the authors and, if the manuscript is accepted for publication, my named report including any attachments I upload will be posted on the website along with the authors' responses. I agree for my report to be made available under an Open Access Creative Commons CC-BY license (http://creativecommons.org/licenses/by/4.0/). I understand that any comments which I do not wish to be included in my named report can be included as confidential comments to the editors, which will not be published.

Choose an item.

To further support our reviewers, we have joined with Publons, where you can gain additional credit to further highlight your hard work (see: https://publons.com/journal/530/gigascience). On publication of this paper, your review will be automatically added to Publons, you can then choose whether or not to claim your Publons credit. I understand this statement.

Yes Choose an item.
