# Supplementary material for: Hecatomb: an integrated software platform for viral metagenomics
Source: Gigascience. 2024 Jun 4;13:giae020. doi: 10.1093/gigascience/giae020 (PMC11148595; doi:10.1093/gigascience/giae020)
Supplement: giae020_Reviewer_2_Report_Original_Submission — Satoshi Hiraoka -- 9/10/2023 Reviewed [file giae020_reviewer_2_report_original_submission.pdf]

Reviewer Report

Title: Hecatomb: An Integrated Software Platform for Viral Metagenomics

Version: Original Submission **Date:** 9/10/2023

Reviewer name: Satoshi Hiraoka

Reviewer Comments to Author:

In this manuscript, the authors developed a novel pipeline, Hecatomb, for viral genome analysis using metagenome and virome data that accepted both short- and long-read sequencing data. Using the pipeline, the authors performed the analysis using one virome and one metagenome dataset from different environments (stool and coral reef, respectively). The analyses showed reasonable results according to the original studies and rather they discovered candidate novel phages and new findings that possibly have great insight into the microbial ecology. The manuscript is overall informative and well-written. The Hecatomb incorporates famous bioinformatics tools that are frequently used in viral genome analyses today, allowing many researchers including beginners to examine virome datasets easily and effectively. Thus the pipeline is likely valuable and would contribute to wide studies of viruses, most of which are not cultured and its characteristics are unknown. Noteworthy, there is an informative document page (https://hecatomb.readthedocs.io/en/latest/) including tutorials, which are very helpful for many users. I think this point could be more emphasized in the manuscript. However, unfortunately, lacking the analysis of the mock dataset makes it hard to estimate the accuracy of the pipeline. I think adding such kinds of analysis for evaluating the performance would greatly improve the study.I have some suggestions that would increase the clarity and impact of this manuscript if addressed.Major:In general, to clearly evaluate the efficiency of the novel bioinformatic tools and pipelines, benchmarking using ground-truth datasets is important in advance to the application using real datasets. To reach this, in this case, some artificial datasets that are composed of known viral and prokaryotic genomes with defined composition and library types (single and paired-end) and sequenced read length (current short- and long-reads) could be designed as mock metagenome data. Via the analysis using the mock datasets, the accuracy of the pipeline can be evaluated. It would be appreciated if the author performed such benchmarking tests as well as the real data applications.According to the GitHub page, the Hecatomb is designed to generate results that reduce false-positive and enrich for true-positive viral read detection. This point is important for understanding the purpose of developing the pipeline and differentiating the pipeline tool from other ones. The efficiency of the false-positive reduction using this pipeline would be better clearly shown in this manuscript. Therefore the mock dataset analyses are expected.When I read the manuscript, I was confused about what the targeted dataset the pipeline aiming for. Is the Hecatomb designed to analyze common prokaryotic shotgun metagenomic data to detect viruses? In other words, is the pipeline not limited to analyzing viral metagenomes (viromes), which specifically enriched viral particles from the samples for sequencing (e.g., density centrifugation to condense viral particles)? The stool samples were likely virome datasets (viral particles were enriched via 0.45-Î¼m-pore-size membrane filtration according to the article), whereas the coral reef data are metagenome datasets. I would suggest that the terms "viral

metagenome" (or virome, specifically targeting only viruses) and common "metagenome" (mainly focusing on prokaryotes) should be clearly distinguished throughout the manuscript including the title.I'm wondering about the sequence clustering step in Module 1. In my understanding, from the metagenomic settings, genomic regions are randomly sequenced, and thus most of the sequenced reads will not be clustered together using the criteria as described in the manuscript, and not so many sequences are reduced in this step. Is this step truly needed? Please add more explanation and importance about this step. For example, how many ratios of the reads were reduced in the test of the two real datasets (stool and coral reef) in this step?Minor:The introduction section is informative but a bit long. The section could be shortened.Some viruses were newly found using the pipeline (e.g., Fig1A). Which one is which virus types (dsDNA, ssDNA, dsRNA, ssRNA)? This information would be better to show clearly in the figure.I think the sequences derived from RNA viruses are generally not abundantly included in typical metagenomics datasets except if with specific techniques in the experiment. I think the potential for detecting RNA viruses from typical metagenomic DNA sequencing reads will be discussed in the Introduction section.L103. Please describe where the name "Hecatomb" is derived from in this article, though this is shown on the GitHub page.L119. " round A/B libraries" here, but I have not heard or could not find this term in the articles cited here. Please add more explanation of what is "round A/B libraries".L130 up to 2 insertions and deletions?L131. BBmap included in BBtools [73]?L181. A brief explanation of the "Baltimore classification" here would improve the readability for readers who are not familiar with this.L239. There is no explanation of what "SIV" means before.L253-L268 & Figure 4B. According to Figure 2A, there are two paths (1,2,5: aa and 1,3,4,5: nt) for detecting viral reads. I'm interested in which path is major and which is minor. Could the authors provide the ratio of the reads that predicted using aa or nt in each dataset examination (each stool and coral)?L431, L436. Not only BioProject but SRA accession ID should be provided.L479. There is no LACC here. What is his main contribution? Just reviewing and editing the manuscript is insufficient for citing as an author: see https://www.icmje.org/recommendations/browse/roles-and-responsibilities/defining-the-role-of-authors-and-contributors.html#twoFigure 1. There are some DBs newly created and used in the pipeline (e.g., Viral AA DB, Multi-kingdom AA DB, Virus NT DB, and Polymicrobial MT DB). I think it would be better to add how to make the DBs in this or other figures. This must contribute to understanding how to construct the DBs and why to use them in this pipeline.Figure 1. specified (1)-(4) in the legend, not just color.Figure 4A. Please provide the total number of sequencing reads in addition to the read count assigned to each virus.Figure 4C. CPM was not explained in the manuscript and not listed in L460.L490. Some references are incomplete. e.g., lack of article ID or page number (49, 79, 90, 94, 95, 96, 100, 101, 102), remaining unnecessary words ("academic.oup.com" in 90, 91), etc. Please check the reference list carefully.Figure S5. Alignment length (bp)Table S2. For calculating the best hit identify, what database was used?

Level of Interest

Please indicate how interesting you found the manuscript: Choose an item.

Quality of Written English

Please indicate the quality of language in the manuscript: Choose an item.

Declaration of Competing Interests

Please complete a declaration of competing interests, considering the following questions:

- Have you in the past five years received reimbursements, fees, funding, or salary from an organisation that may in any way gain or lose financially from the publication of this manuscript, either now or in the future?
- Do you hold any stocks or shares in an organisation that may in any way gain or lose financially from the publication of this manuscript, either now or in the future?
- Do you hold or are you currently applying for any patents relating to the content of the manuscript?
- Have you received reimbursements, fees, funding, or salary from an organization that holds or has applied for patents relating to the content of the manuscript?
- Do you have any other financial competing interests?
- Do you have any non-financial competing interests in relation to this paper?

If you can answer no to all of the above, write 'I declare that I have no competing interests' below. If your reply is yes to any, please give details below.

I declare that I have no competing interests.

I agree to the open peer review policy of the journal. I understand that my name will be included on my report to the authors and, if the manuscript is accepted for publication, my named report including any attachments I upload will be posted on the website along with the authors' responses. I agree for my report to be made available under an Open Access Creative Commons CC-BY license (http://creativecommons.org/licenses/by/4.0/). I understand that any comments which I do not wish to be included in my named report can be included as confidential comments to the editors, which will not be published.

Choose an item.

To further support our reviewers, we have joined with Publons, where you can gain additional credit to further highlight your hard work (see: https://publons.com/journal/530/gigascience). On publication of this paper, your review will be automatically added to Publons, you can then choose whether or not to claim your Publons credit. I understand this statement.

Yes Choose an item.
